# Impact of finish line designs on the adaptation of ceramic fixed dental prostheses: a systematic review and network meta-analysis

**DOI:** 10.1186/s12903-025-06433-0

**Published:** 2025-07-03

**Authors:** Adrienn Pál, Petra Papócsi, Kata Kelemen, Bianca Golzio Navarro Cavalcante, Péter Hegyi, Beáta Kerémi, Noémi Gede, Péter Hermann, Zoltán Géczi, Ivett Róth

**Affiliations:** 1 Centre for Translational Medicine, Semmelweis University, Budapest, Hungary; 2 Department of Prosthodontics, Semmelweis University, Budapest, Hungary; 3 Department of Oro-Maxillofacial Surgery and Stomatology, Semmelweis University, Budapest, Hungary; 4 Institute of Pancreatic Diseases, Semmelweis University, Budapest, Hungary; 5 Translational Pancreatology Research Group, Interdisciplinary Centre of Excellence for Research Development and Innovation, University of Szeged, Szeged, Hungary; 6 Department of Restorative Dentistry and Endodontics, Semmelweis University, Budapest, Hungary; 7 Institute for Translational Medicine, Medical School, University of Pécs, Pécs, Hungary

**Keywords:** Chamfer, Shoulder, Rounded shoulder, Vertical tooth preparation, Ceramics, Dental marginal adaptation*, Dental prosthesis design, Dental porcelain

## Abstract

**Background:**

Insufficient marginal and internal adaptation can lead to secondary caries, pulp infection, periodontal disease, and tooth loss. This network meta-analysis (NMA) assessed the impact of various finish line designs on the marginal and internal adaptation of ceramic fixed dental prostheses.

**Methods:**

In vitro studies were conducted comparing the adaptation of full-ceramic fixed dental prostheses with various preparation designs (chamfer, shoulder, rounded shoulder, and vertical). An electronic search of four major databases (Cochrane Library, EMBASE, PubMed, Web of Science) on April 1, 2025, identified 4,714 studies. After screening, review authors found 50 studies eligible for qualitative analysis and 34 for quantitative analysis. NMA and pairwise comparisons assessed marginal gap (MG), internal gap (IG), and absolute marginal discrepancy (AMD). The subset analysis considered the presence of cementation, manufacturing techniques, restoration types and evaluation techniques. The QUIN tool evaluated the risk of bias, while GRADE PRO and CINEMA assessed certainty of result.

**Results:**

This NMA identified the chamfer preparation as the most favorable for smaller internal gap, the vertical preparation as the most effective for smaller marginal gap, and the rounded shoulder as the top performer for smaller absolute marginal discrepancy (AMD). Although no statistically significant differences in marginal fit (MG and AMD) were found among the tested designs (vertical, chamfer, rounded shoulder, and shoulder), the significantly superior internal adaptation (MD: 26.65 μm) associated with the chamfer finish line supports its recommendation for clinical use.

**Conclusions:**

The findings suggest that although marginal fit differences (MG, AMD) among finish line designs may not be statistically significant, the internal adaptation advantage of the chamfer finish line supports its clinical recommendation. The discrepancies observed between internal and marginal fit emphasize the critical role of cement space optimization to achieve balanced adaptation, which is key to enhancing the longevity and clinical success of ceramic restorations.

**Supplementary Information:**

The online version contains supplementary material available at 10.1186/s12903-025-06433-0.

## Background

Ceramic fixed dental prostheses (FDPs) are widely used in dentistry due to their natural esthetics, durability, and biocompatibility [1]. Advances in CAD/CAM technology have allowed the use of high-strength ceramics for posterior restorations, while materials with modest mechanical strength, such as ultra-translucent zirconia and glass ceramics remain preferred for anterior regions [2, 3].

Ensuring proper marginal and internal adaptation is crucial for the longevity and clinical success of FDPs. Poor adaptation can lead to plaque accumulation, secondary caries, pulp infections, gingival inflammation, and even tooth loss [4, 5].

Marginal adaptation is typically assessed by measuring the marginal gap (the perpendicular distance between the internal surface of the prosthesis and the prepared tooth at the margin) or the absolute marginal discrepancy, which accounts for both the marginal gap and any extension errors [6]. The internal gap refers to the perpendicular distance between the internal surface of the prosthesis and the axial walls of the preparation [6].

Various 2D and 3D techniques are used to evaluate marginal and internal fit. Non-destructive methods include direct microscopic examination and the silicone replica technique, while destructive techniques such as cross-sectional analysis provide highly accurate assessments but require sectioning of the specimen [5, 7]. More advanced methods, such as X-ray microtomography, allow digital, non-destructive 3D evaluations with high precision [5, 7].

Several factors influence the marginal and internal fit of FDPs, including the type of tooth preparation, measurement techniques, manufacturing precision (conventional vs. CAD/CAM), setting of the cement space, and cementation protocols [5, 8, 9].

Tooth preparation design is crucial for adaptation, stress distribution, and long-term stability [5]. Horizontal (e.g., chamfer, shoulder, and rounded shoulder) and vertical (e.g., feather edge, knife edge, and biologically oriented preparation technique [BOPT]) techniques are used for fixed dental prostheses. Horizontal preparations offer clear, well-designed margins, while vertical are more conservative [10–12]. Although chamfer and rounded shoulder preparations have been extensively investigated [13], there is limited evidence on the impact of vertical preparation designs, which are increasingly used due to their minimally invasive approach [14, 15].

Understanding how different preparation designs affect the marginal and internal fit of ceramic FDPs is essential for optimizing clinical outcomes.

**Fig. 1:**
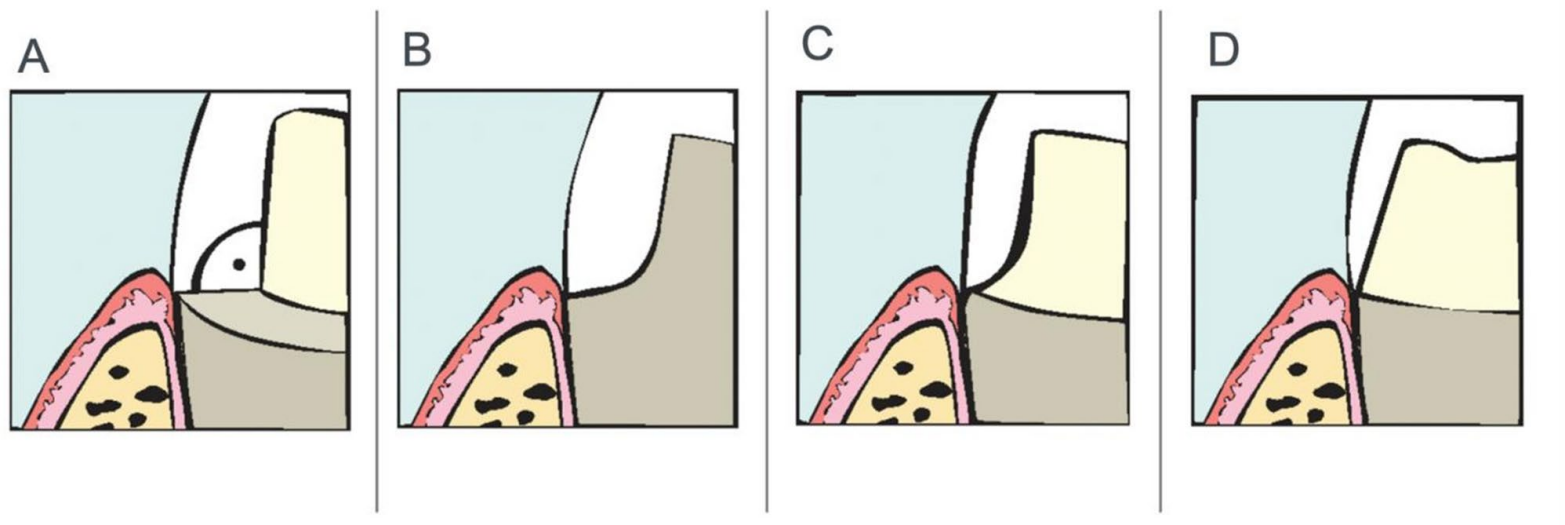
Preparation designs **A**: Shoulder, **B**: Rounded shoulder, **C**: Chamfer and **D**: Vertical preparations [16]

This systematic review and network meta-analysis aimed at comparing chamfer, shoulder, rounded shoulder, and vertical preparations (Fig. 1 [16]), in terms of marginal and internal adaptation. The null hypothesis proposed no significant difference in marginal and internal fit among these preparation designs.

## **Methods**

### Protocol and registration

The present systematic review and meta-analysis was reported based on the recommendation of the PRISMA 2020 guideline [17], Supplementary Material 1. The study protocol was registered in PROSPERO (registration number CRD42023471601) and was fully adhered to.

### Study eligibility criteria

The Population, Intervention, Comparator, and Outcome (PICO) framework was applied to the review question. The population consisted of ceramic fixed dental prostheses, where the interventions and comparators were rounded shoulder, chamfer, shoulder, and vertical preparation, and the outcome was marginal and internal fit in µm (Table 1). This systematic review included articles that met specific criteria: studies assessing the marginal and/or internal adaptations of ceramic fixed dental prostheses; two- or multi-armed in vitro studies that evaluated fixed dental prostheses with rounded shoulder, chamfer, shoulder or vertical preparation; and investigations with well-documented measurements of marginal and/or internal adaptation. Articles that met any of the following criteria were excluded: articles focusing on materials other than ceramics, protocols, clinical guidelines, reviews, meta-analyses, or letters to the editor. Studies that did not report the sample size, mean, and standard deviation (SD) for each group were excluded from the meta-analysis, but were still included in the systematic review. There were no language or date restrictions.

**Table 1:** PICO table

Population	Intervention/Comparator	Outcome
Fixed Dental Prostheses	Chamfer, Shoulder, Rounded shoulder, Vertical preparation	Marginal fit Internal fit (µm)

### Information sources and search strategy

A systematic search was conducted in four databases (MEDLINE via PubMed, CENTRAL [The Cochrane Central Register of Controlled Trials], Embase, and Web of Science) on April 1, 2025. The detailed search keys can be found in Supplementary Material 2.

### Selection process

Rayyan [18] reference management tool was used for the selection process. After removing duplicates, two review authors (AP and PP) performed the selection independently by title and abstract. Afterward, the two review authors carefully read full texts to determine eligibility. Cohen’s Kappa (κ) coefficient was computed at each level. Discrepancies between the review authors were resolved through discussion facilitated by a third review author (KK).

### Data collection process and data items

Two review authors independently extracted data from the included studies using a data sheet prepared in advance in Microsoft Excel 365 (Microsoft Corporation, Redmond, Washington). These data included first author, year of publication, study design, preparation design, total occlusal convergence, height of abutment, shoulder width, ceramic material tested, stage of FDPs, number of subjects involved, number of measurement points, means and standard deviations of internal gap/marginal gap/absolute marginal discrepancy, impression technique, occurrence of cementation, evaluation method and magnification, and cement space and whether any adjustment was made. Additionally, information was extracted to evaluate the risk of bias and the certainty of the evidence.

### Study risk of bias assessment

Two review authors (PA, PP) independently performed the risk of bias assessment using the Quality Assessment Tool For In Vitro Studies (QUIN Tool) [19]. If necessary, a third review author (KK) was also involved in the decision making. All articles were assessed based on 12 specific criteria [19]. A score was assigned to each criterion according to the level of specification: 2 points if the criterion was well specified, 1 point if it was not well specified, 0 points if it was not specified at all, and marked as not applicable if the criterion did not pertain to the article, in which case it was excluded from scoring [19]. Individual scores were summed to generate a total score, which was then converted into a percentage for each in vitro study. Using a defined formula, the review authors classified the risk level of the study as high, medium, or low. Scientists employed the QUIN Tool to evaluate the likelihood of bias in particular in vitro studies [19]. (Supplementary Material 3)

### Certainty assessment

Quality and certainty assessments of the studies included were performed according to the GRADE handbook (Supplementary Material 4, Tables S25, S26 and S27), using the GRADE-PRO website [20].

The GRADEpro tool assessed the strengths and limitations of the results and the certainty of evidence. This evaluation considered key domains, including study design, risk of bias, inconsistency, indirectness, and imprecision. All outcomes were classified into one of four levels of certainty: high, moderate, low, or very low [20].

The CINEMA (Confidence in Network Meta-Analysis) assessment was used to evaluate the certainty of evidence in network meta-analyses (NMAs) [21]. It is based on the GRADE approach and assesses six key domains: Within-study bias, Reporting bias, Indirectness, Imprecision, Heterogeneity and Incoherence [21]. (Supplementary Material 4, Tables S1-S24.)

The assessment was performed independently by two authors (AP, PP). In cases of disagreement, a third review author was involved (KK).

### Effect measures and syntheses methods

The main outcome measures of this systematic review and network meta-analysis were the marginal gap, internal gap and absolute marginal discrepancy. Where sufficient data were available, subset analyses were conducted to identify potential sources of heterogeneity. Subset analyses were performed based on cementation (with and without cementation), manufacturing techniques (CAD/CAM and conventional manufacturing), restoration types (crowns, endocrowns, copings, veneers, occlusal veneers and small span bridges) and evaluation techniques (direct view technique, cross-sectioning method, micro-CT, silicone replica technique).

Pairwise meta-analyses and network meta-analyses (NMAs) were conducted using Bayesian methods with the random-effects model. The examination of the consistency was ruled out by visual inspection of plots. The mean difference was used for continuous data with 95% credible intervals (95% CrI). To calculate the study MDs, we extracted the sample size, mean, and corresponding standard deviation (SD) from each study (in each group separately). The model was optimized and posterior samples were generated using the Monte-Carlo methods running in four chains. A minimum of 10,000 adaptation iterations was set to obtain convergence, along with 20,000 simulation iterations. Interventions were ranked according to the posterior probability by calculating the surface under cumulative ranking (SUCRA) curve values. SUCRA values range from 0 to 100%. The higher the SUCRA value, and the closer it is to 100%, the higher the probability that a therapy is ranked in the top rank or one of the top ranks; the closer the SUCRA value is to 0, the more likely it is that a therapy is ranked in the bottom rank, or one of the bottom ranks. All calculations were performed with R (V. 4.1.1) package BUGSnet (V. 1.1.0) along with the Markov Chain Monte Carlo engine JAGS (V. 4–12).

## Results

### Study selection

**Fig. 2:**
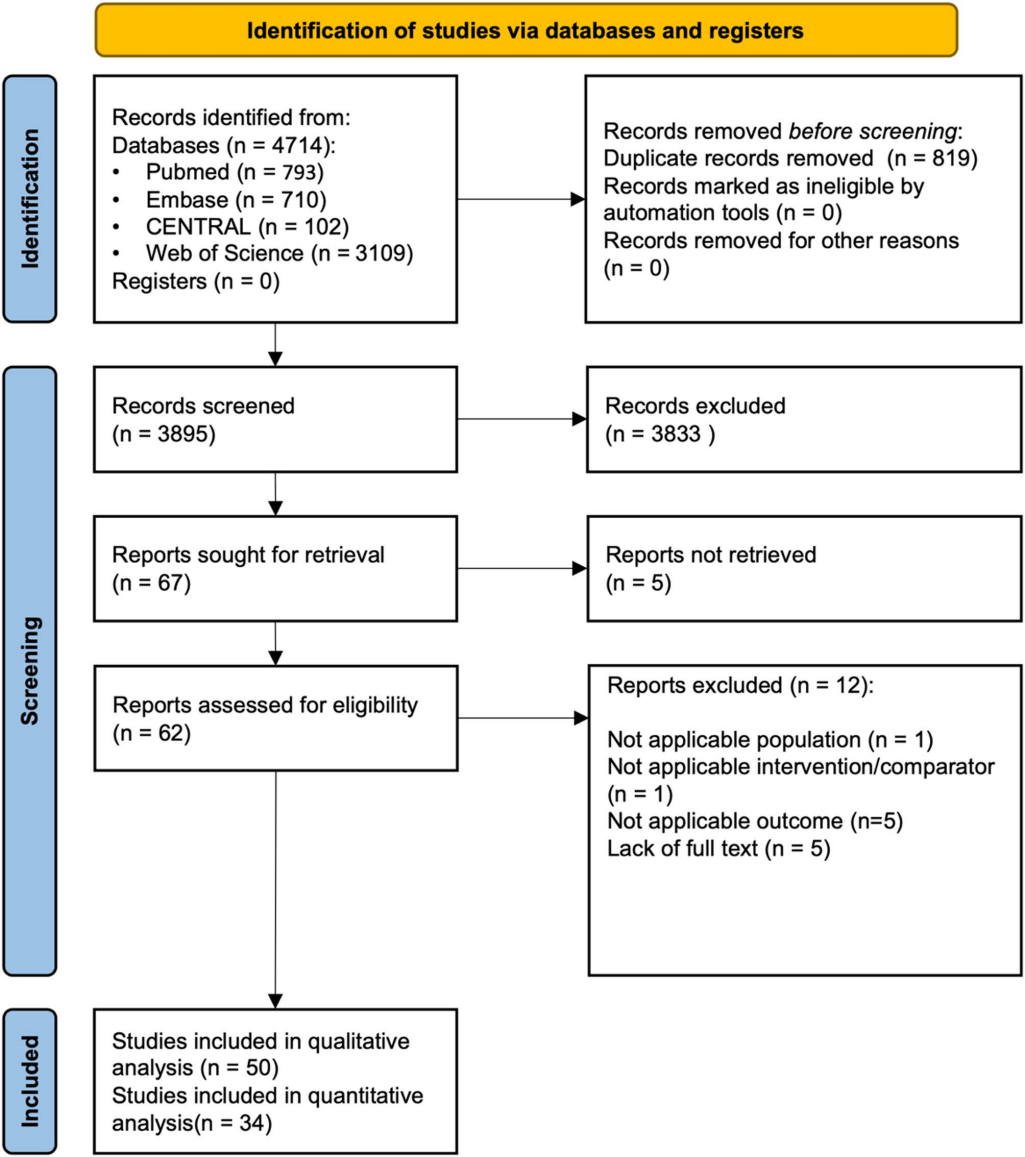
PRISMA 2020 flowchart representing the study selection process

A total of 4,714 records were identified, of which 3,895 studies remained after duplicate removal. After selection by title and abstract, 67 articles were selected for full-text reading (κ = 0.94). Five articles could not be retrieved for full-text screening. The remaining 62 full texts were carefully read by the review authors (κ = 0.95). One article was excluded because it did not investigate ceramic fixed dental prostheses, and another one because it investigated different kinds of interventions. Five studies were excluded because outcomes were not applicable. Fifty articles proceeded for qualitative synthesis, of which 34 were selected for quantitative analysis. A manual search of reference lists yielded no further results. Figure 2 shows the PRISMA flowchart of study selection.

### Study characteristics

The studies included were published in English, German, or Chinese between 1994 and 2025. All 50 eligible studies [22–71] were in vitro studies investigating ceramic fixed dental prostheses. Six measurement methods were used to examine the marginal adaptation of ceramic fixed dental prostheses with different preparation designs, of which the direct-view technique was most commonly used (29 studies) [22, 23, 25–29, 32, 35, 37, 38, 41, 43–47, 49, 52, 53, 55–57, 62, 64–66, 69, 71], followed by the cross-sectioning method (9 studies) [30, 31, 33, 40, 42, 54, 61, 63, 68], microcomputed X-ray tomography (micro-CT) (5 studies) [34, 36, 48, 59, 70], replica technique (2 studies) [50, 67], superimposition (1 study) [39], and profilometry (1 study) [51]. For internal adaptation, 9 studies [24, 30, 31, 49, 58, 60, 61, 63, 68] measured the internal gap of fixed dental prostheses using the cross-sectioning method, 6 studies [27, 47, 52, 57, 62, 67] measured FDPs using the replica technique, and 3 studies [36, 59, 70] utilized microcomputed X-ray tomography (micro-CT). The baseline characteristics of the studies included are presented in Supplementary Material 5.

### Quantitative analysis

#### Marginal gap analysis

**Fig. 3:**
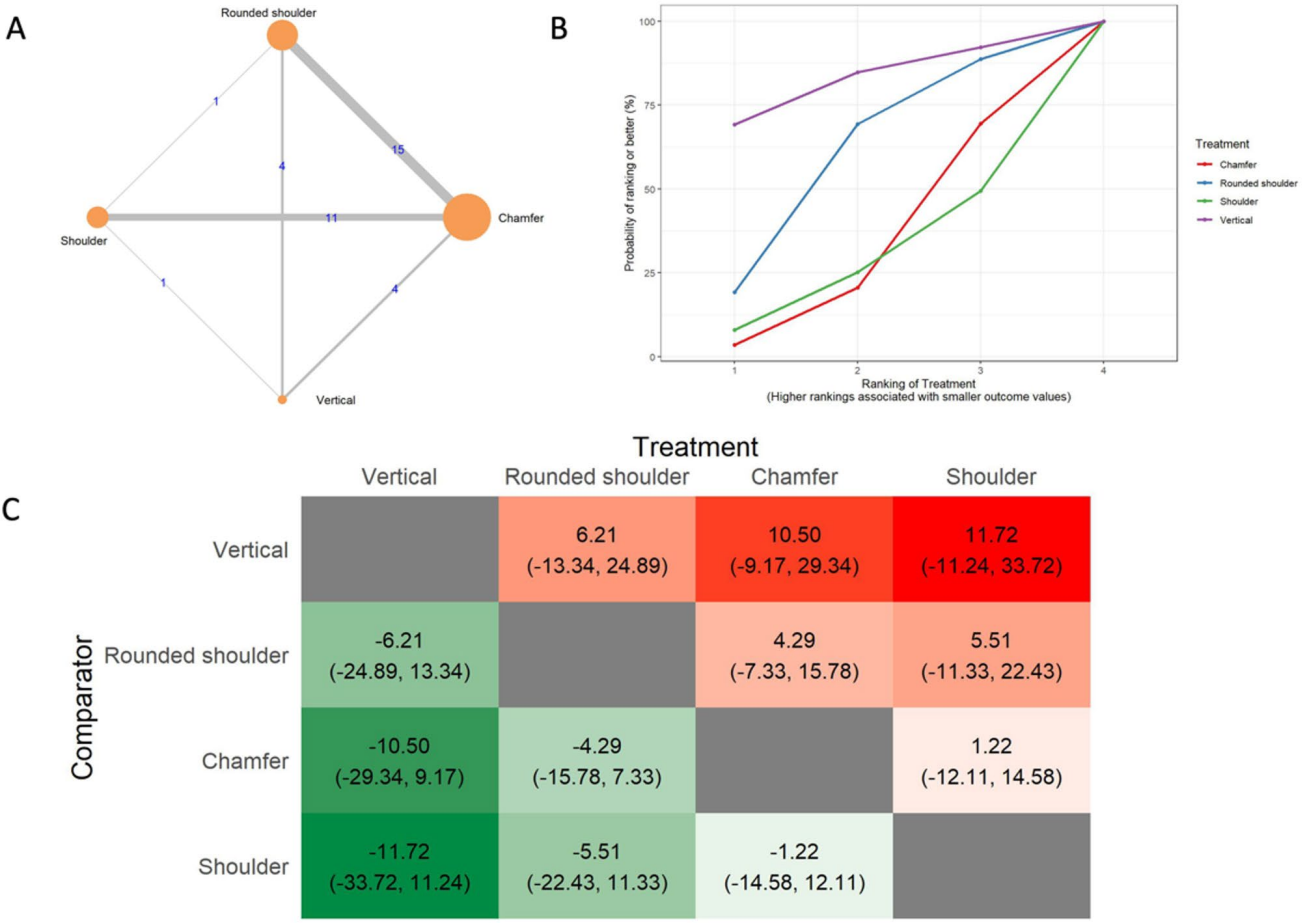
Marginal gap **A**: Network geometry of the eligible comparisons of the marginal gap for rounded shoulder, shoulder, chamfer, and vertical preparation designs, **B**: Surface under the cumulative ranking curves (SUCRA%) values of marginal gap values, **C**: The league heat diagram shows the mean difference (MD) and 95% credible interval in µm for all possible treatment pairs, **D**: The forest plot shows the mean differences and 95% credible interval relative to the rounded shoulder

The results of the marginal gap (MG) analysis include a network of 28 in vitro studies, 24 two-arm studies, and 4 multi-arm studies (Fig. 3A). The total number of examined ceramic fixed dental prostheses in the network was 1,232. SUCRA values (Fig. 3B) indicated that vertical preparation is likely to have the smallest marginal gap (SUCRA: 82.11%), followed by rounded shoulder (SUCRA: 59.11%), chamfer (SUCRA: 31.2%), and shoulder preparation (SUCRA: 27.57%). The league heat plot for the marginal gap (Fig. 3C) shows the pairwise comparisons of different preparation techniques. When the vertical preparation was compared to the rounded shoulder (MD: 6.21 μm, CrI: -13.34, 24.89), chamfer (MD: 10.5 μm, CrI: -9.17, 29.34), and shoulder preparation techniques (MD: 11.72 μm, CrI: -11.24, 33.72), the vertical preparation was favored. No statistically significant differences were detected between the different preparation designs. The consistency analysis (Supplementary Material 6, Fig. 1) showed that the comparisons in the network were consistent.

#### Absolute marginal discrepancy analysis

**Fig. 4:**
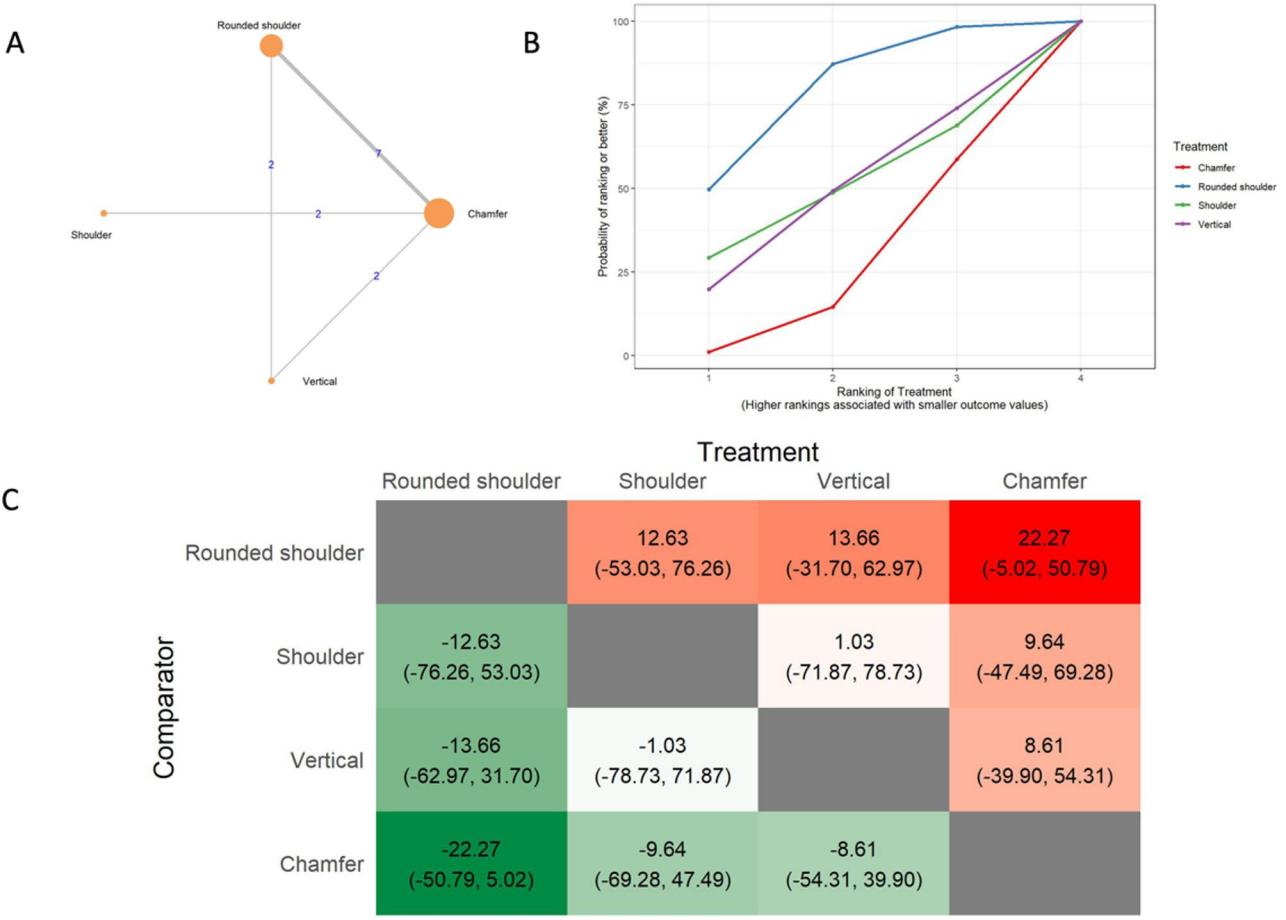
Absolute marginal discrepancy **A**: Network geometry of the eligible comparisons of absolute marginal discrepancy for rounded shoulder, shoulder, chamfer and vertical preparation designs, **B**: Surface under the cumulative ranking curves (SUCRA%) values of absolute marginal discrepancy values, **C**: The league heat diagram shows the mean difference (MD) and 95% credible interval in µm for all possible treatment pairs, **D**: The forest plot shows the mean differences and 95% credible interval relative to the rounded shoulder

The results of the absolute marginal discrepancy (AMD) analysis include a network of 9 in vitro studies, 7 two-arm studies, and 2 multi-arm studies. (Fig. 4A) The total number of examined ceramic fixed dental prostheses in the network was 366. SUCRA values (Fig. 4B) indicated that rounded shoulders were likely to have the smallest absolute marginal discrepancy (SUCRA: 78.42%), followed by the shoulder (SUCRA: 49.01%), vertical (SUCRA: 47.76%), and chamfer preparations (SUCRA: 24.83%). The league heat plot for the absolute marginal discrepancy (Fig. 4C) shows pairwise comparisons of different preparation techniques. When the rounded shoulder was compared to the shoulder (MD: 12.63 μm CrI: -53.03, 76.26), vertical (MD: 13.66 μm CrI: -36.70, 62.97), and chamfer preparation technique (MD: 22.27 μm CrI: -5.02, 50.79), the rounded shoulder was favored. The mean differences were not statistically significant. The consistency analysis (Supplementary Material 6, Fig. 2) showed that the comparisons in the network were consistent.

#### Internal fit analysis

**Fig. 5:**
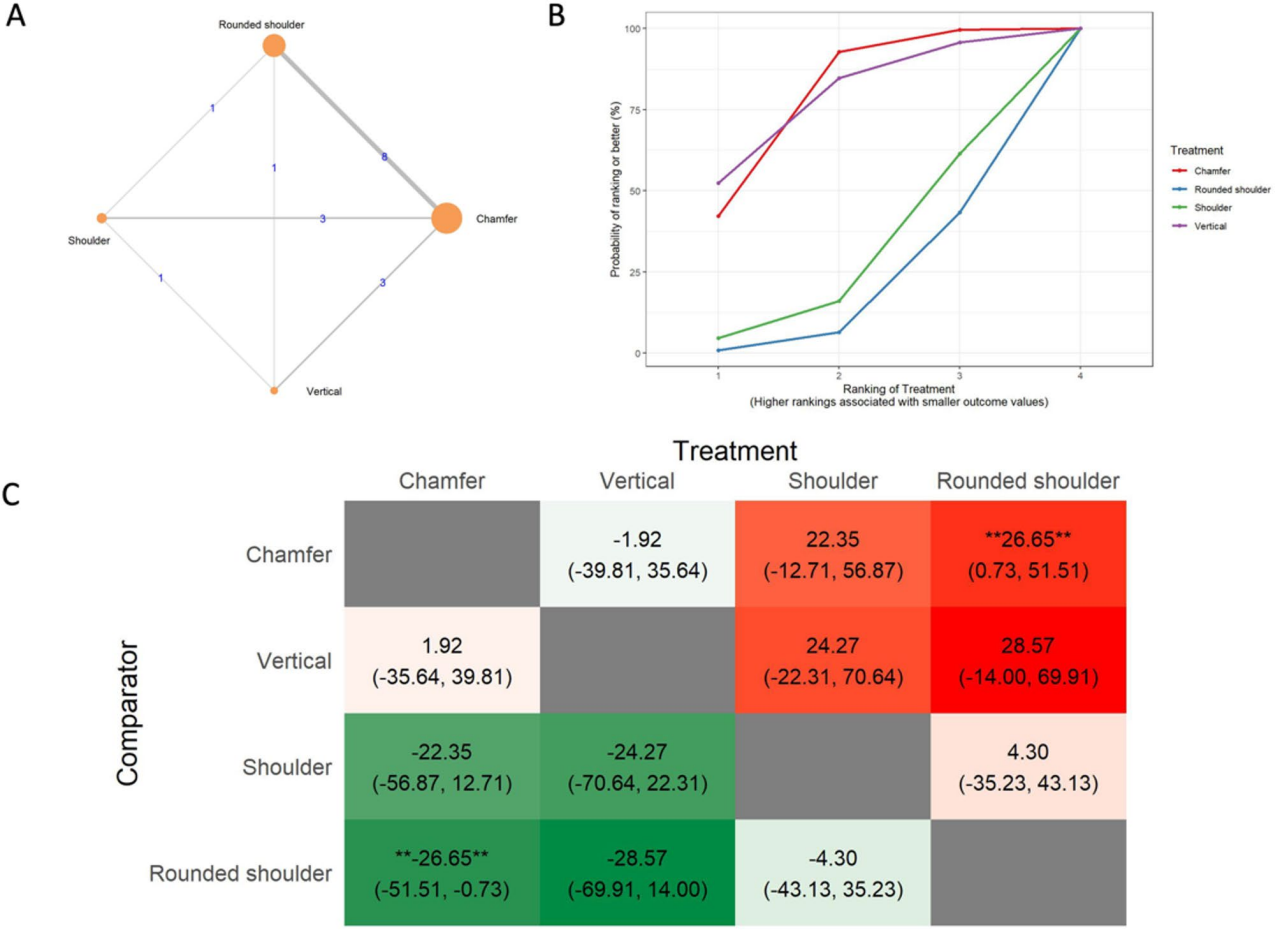
Internal gap **A**: Network geometry of the eligible comparisons of the internal gap for rounded shoulder, shoulder, chamfer, and vertical preparation designs, **B**: Surface under the cumulative ranking curves (SUCRA%) values of internal gap values, **C**: The league heat diagram shows the mean difference (MD) and 95% credible interval in µm for all possible treatment pairs, **D**: The forest plot shows the mean differences and 95% credible interval relative to the rounded shoulder

The results of the internal gap (IG) analysis included the network of 13 in vitro studies, 11 two-arm studies, and 2 multi-arm studies. (Fig. 5A) The total number of ceramic fixed dental prostheses examined in the network was 422. SUCRA values (Fig. 5B) indicated that chamfer preparation designs were likely to have the smallest internal gap values (SUCRA: 78.17%), followed by vertical (SUCRA: 77.59%), shoulder (SUCRA: 27.41%), and the rounded shoulder preparations (SUCRA: 16.85%). The league heat plot for the internal gap (Fig. 5C) shows pairwise comparisons of different preparation techniques. When the chamfer preparations were compared with the vertical preparations (MD: -1.92 μm CrI: -39.81, 35.64) and the shoulder preparations (MD: 22.35 μm CrI: -12.71, 56.87), no statistically significant differences were observed. However, when the chamfer preparation was compared with the rounded shoulder (MD: 26.65 μm CrI: 0.73, 51.51), statistically significant differences favoring the chamfer were observed. The consistency analysis (Supplementary Material 6, Fig. 3) showed that the comparisons in the network were consistent.

#### Subset analysis

Subset analyses were conducted to identify potential sources of heterogeneity. Subset analyses were performed based on cementation (with and without cementation) and manufacturing techniques (CAD/CAM and conventional manufacturing), restoration types (crowns, endocrowns, copings, veneers, occlusal veneers and small-span bridges) and evaluation techniques (direct view technique, cross-sectioning method, micro-CT, and silicone replica technique).

The mean differences between the four preparation designs were not statistically significant for cemented and uncemented groups and for ceramic single-unit restorations (crowns, endocrowns, copings, veneers, and occlusal veneers).

**Fig. 6:**
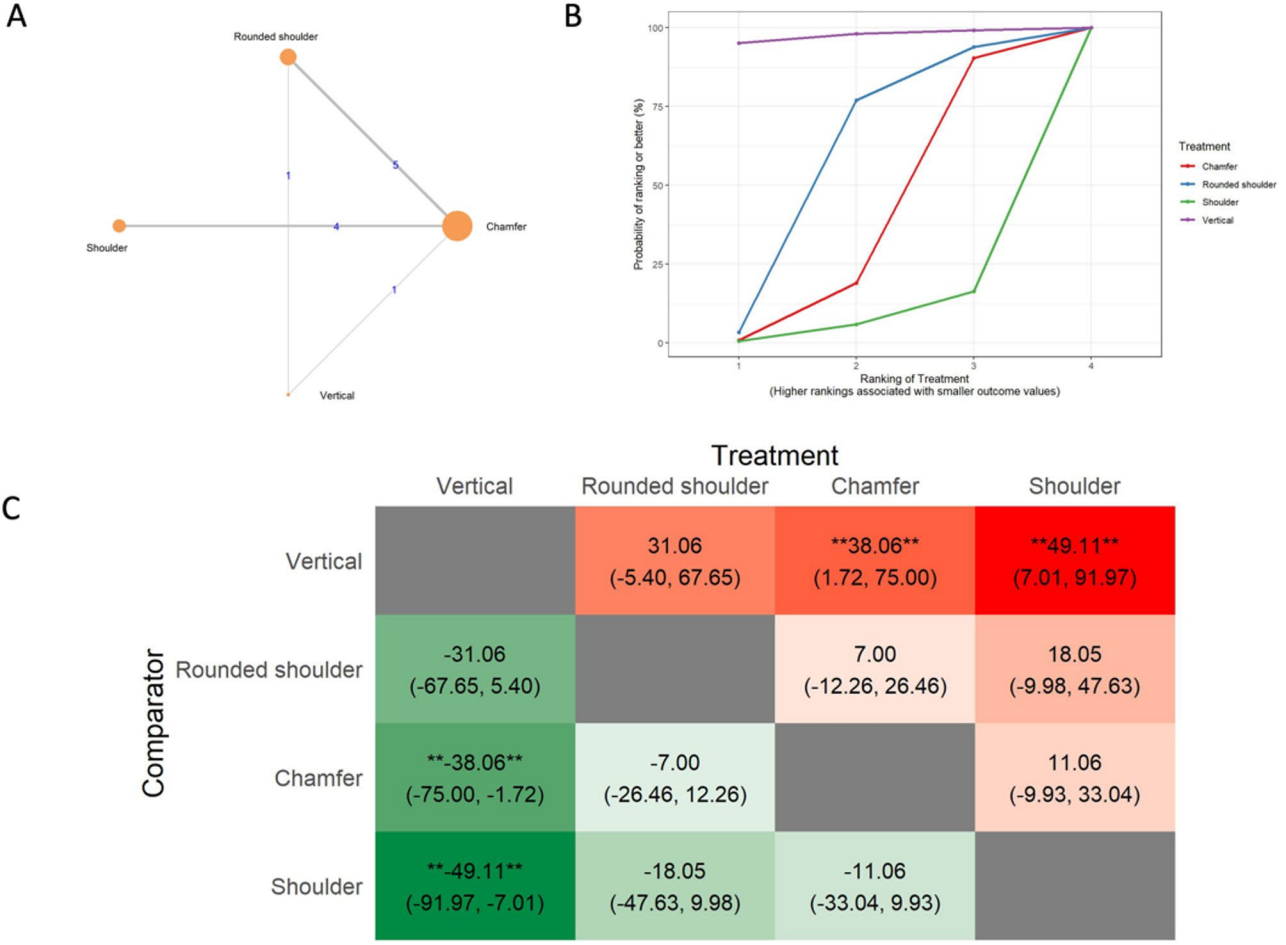
Marginal gap of the conventional all-ceramic system **A**: Network geometry of the eligible comparisons of the internal gap of the marginal gap of the conventional all-ceramic system for rounded shoulder, shoulder, chamfer, and vertical preparation designs, **B**: Surface under the cumulative ranking curves (SUCRA%) values of marginal gap values, **C**: The league heat diagram shows the mean difference and 95% credible interval for all possible treatment pairs in µm

For the marginal gap of conventional all-ceramic systems (Fig. 6), the vertical preparation showed statistically better results than chamfer (MD: 38.06 μm CrI: 1.72, 75.00) and shoulder (MD: 49.11 μm CrI: 7.01, 91.97).

**Fig. 7:**
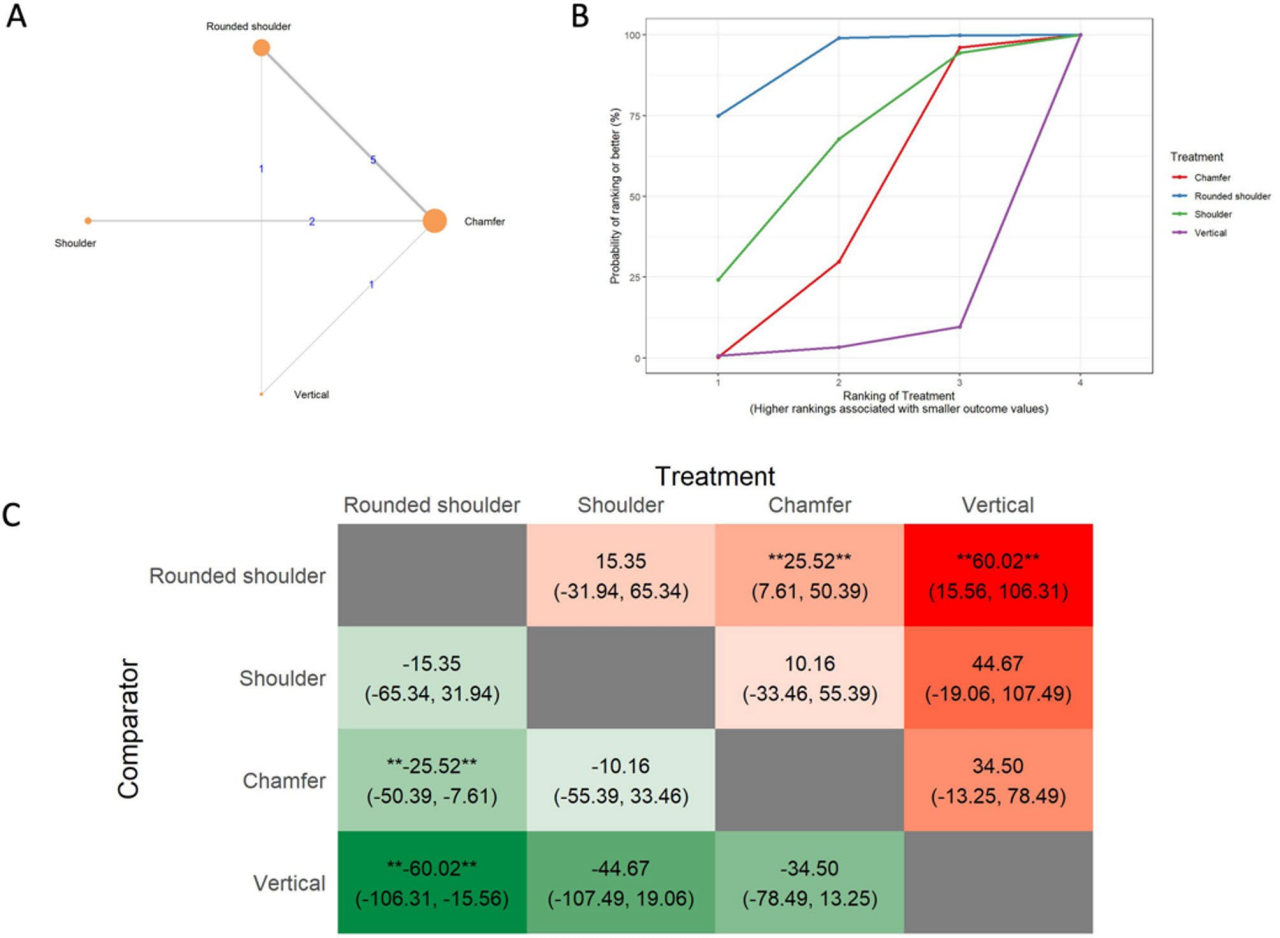
Absolute marginal discrepancy of CAD/CAM all-ceramic system **A**: Network geometry of the eligible comparisons of the absolute marginal discrepancy of CAD/CAM all-ceramic system for rounded shoulder, shoulde, chamfer, and vertical preparation designs, **B**: Surface under the cumulative ranking curves (SUCRA%) values of marginal gap values, **C**: The league heat diagram shows the mean difference and 95% credible interval for all possible treatment pairs in µm

**Fig. 8:**
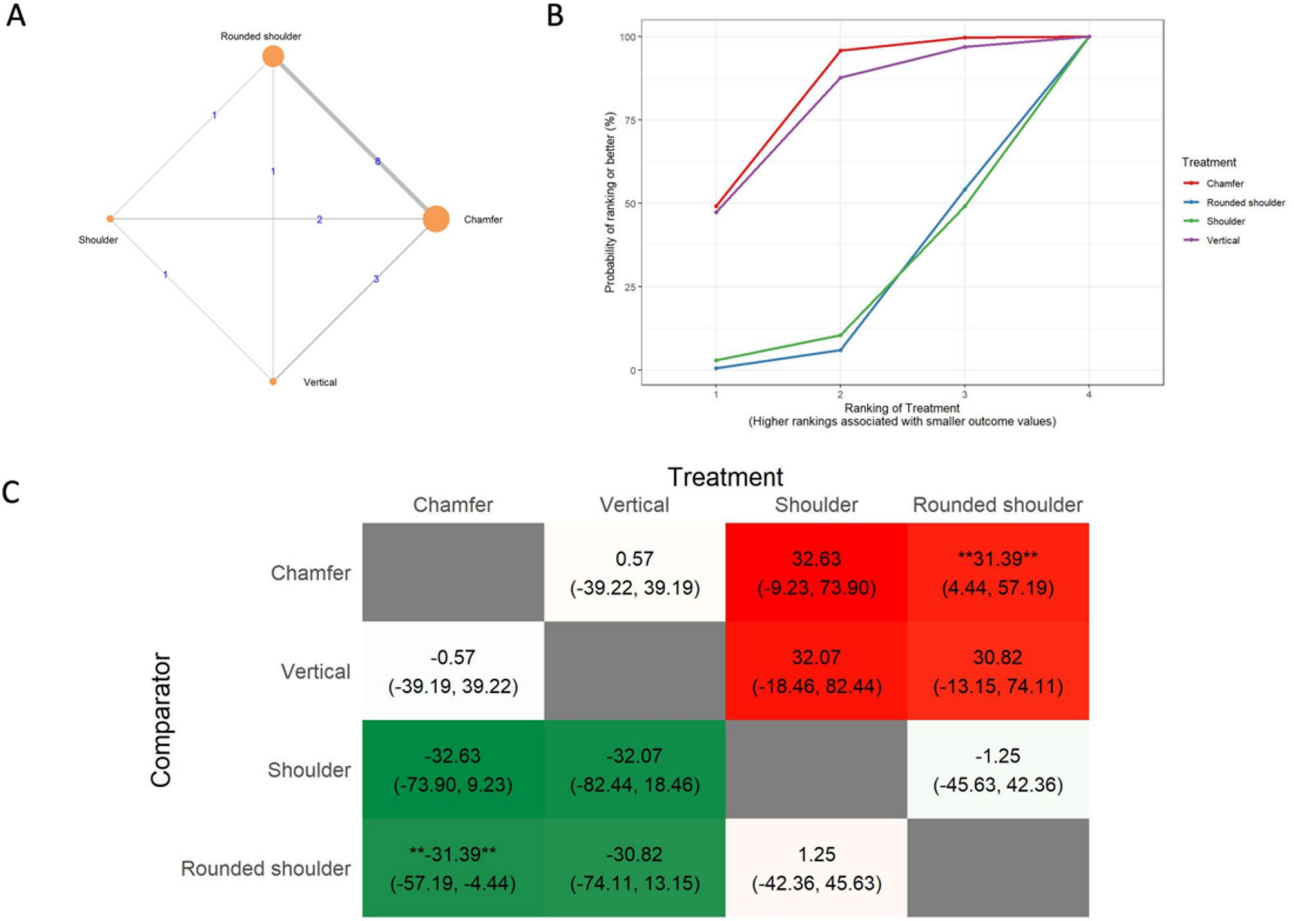
Internal gap of CAD/CAM all-ceramic system **A**: Network geometry of the eligible comparisons of the internal gap of CAD/CAM all-ceramic system for rounded shoulder, shoulder, chamfer, and vertical preparation designs, **B**: Surface under the cumulative ranking curves (SUCRA%) values of marginal gap values, **C**: The league heat diagram shows the mean difference and 95% credible interval for all possible treatment pairs in µm

In the CAD/CAM all-ceramic system, we found contrasting results between AMD and IG. For the absolute marginal discrepancy of CAD/CAM all-ceramic system (Fig. 7), the rounded shoulder showed statistically better results than the vertical preparation (MD: 60.02 μm CrI: 15.56, 106.21) and the chamfer preparation (MD: 25.52 μm CrI: 7.61, 50.39). In terms of the internal gap of the CAD/CAM all-ceramic system (Fig. 8), chamfer showed significantly better results than rounded shoulder (MD: 31.39 μm CrI: 4.44, 57.19).

**Fig. 9:**
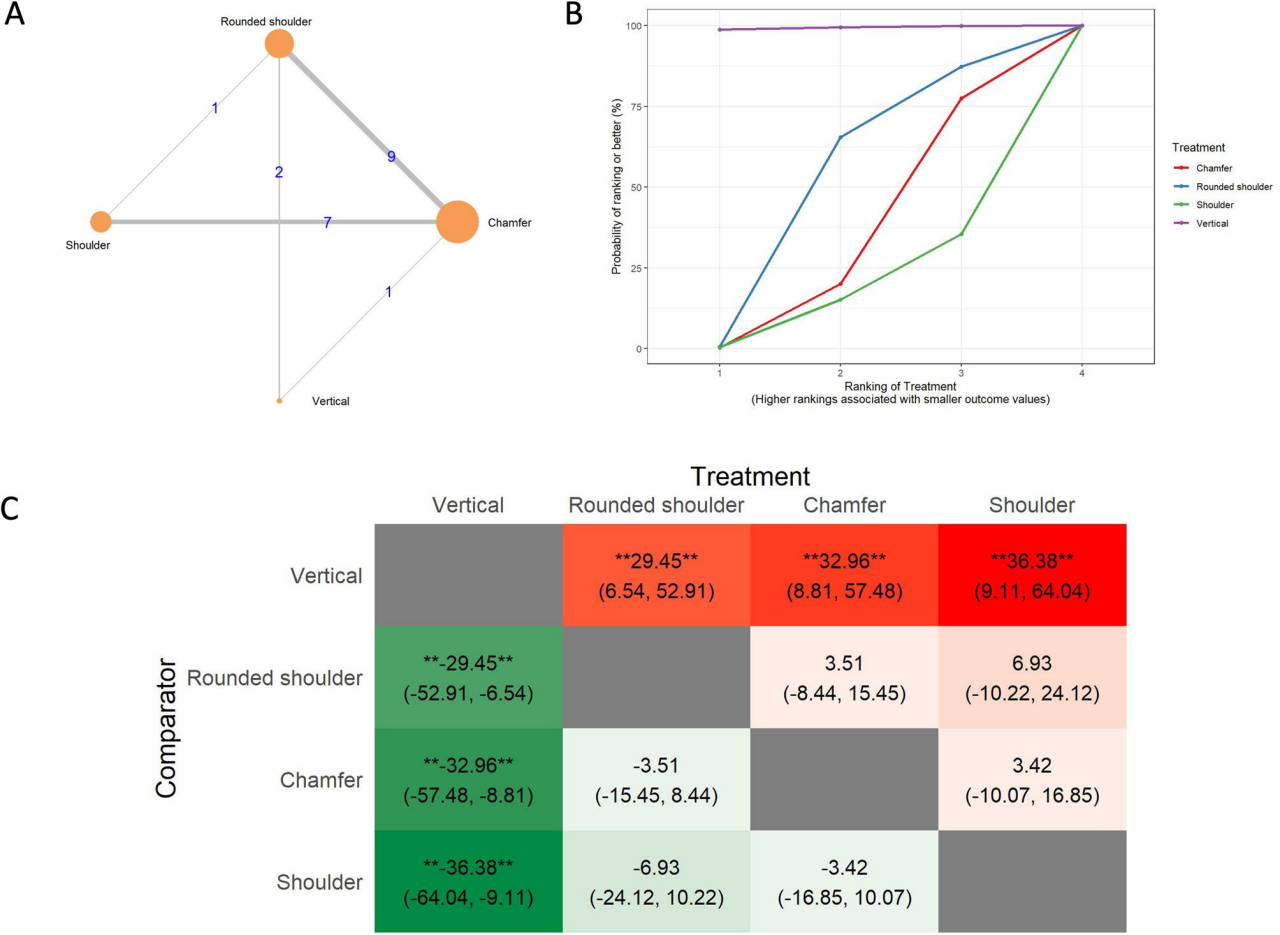
Marginal gap evaluation using the direct view technique **A**: Network geometry of the eligible comparisons of the internal gap of the marginal gap of the conventional all-ceramic system for rounded shoulder, shoulder, chamfer, and vertical preparation designs, **B**: Surface under the cumulative ranking curves (SUCRA%) values of marginal gap values, **C**: The league heat diagram shows the mean difference and 95% credible interval for all possible treatment pairs in µm

In the subgroup analysis of evaluation techniques, we obtained controversial results. In the marginal gap evaluation using the direct view technique, the vertical preparation showed significantly smaller marginal gap values compared to the rounded shoulder (MD: 29.45 μm CrI: 6.54, 52.91), chamfer (MD: 32.96 μm CrI: 8.81, 57.48), and shoulder (MD: 36.38 μm CrI: 9.11, 64.04 (Fig. 9). However, in the marginal gap evaluation using the micro-CT technique, the vertical preparation yielded the worst results, with an MD of 68.81 μm compared to the rounded shoulder.

The subgroup division and the results of the analysis are detailed in Supplementary Material 7. The consistency analysis (Supplementary Material 6, Figs. 4–31) showed that comparisons in the network were consistent.

#### Qualitative analysis

Some studies measured the vertical and horizontal marginal discrepancy values. These studies could only be included in qualitative analysis. The vertical marginal discrepancy refers to misalignment in the vertical plane, parallel to the draw path of the casting [6]. In contrast, horizontal marginal discrepancy occurs in the horizontal plane, perpendicular to the draw path [6].

### Vertical marginal discrepancy

In three studies, Souza et al., Rinke et al., and Suárez et al. concluded that the rounded shoulder finish line resulted in significantly lower vertical marginal discrepancy values compared to the chamfer finish line [58, 62, 63].

Re et al. found no statistically significant difference between chamfer and rounded shoulder; however, the lowest vertical marginal discrepancy values were obtained for rounded shoulder [56]. Quintas et al. also found no significant differences in vertical marginal discrepancy associated with the finish line [55].

Komine et al. compared shoulder, rounded shoulder, and chamfer preparation designs and found that the finish line design did not impact vertical marginal discrepancy values in single tooth zirconium dioxide copings and crowns [47].

Ates et al. investigated zirconia copings before and after veneering on dies with shoulder and chamfer finish lines [26]. The study determined that, following esthetic veneering, copy-milled copings fabricated with a chamfer finish line failed to achieve dimensions within clinically acceptable parameters [26].

### Horizontal marginal discrepancy

In terms of horizontal marginal discrepancy, Suárez et al. found no significant difference between chamfer and rounded shoulder finish lines. They found that both finish lines exhibited horizontal overcontouring [63].

In 2010, Baig et al. found that zirconia CAM crowns with rounded shoulder finish lines performed significantly better than chamfer ones in marginal overhang [29]. However, in 2022, they reported no significant difference in overhang between these finish lines in zirconia and lithium disilicate crowns [28].

### Qualitative analysis of MG, IG, and AMD values

Ribeiro et al. [57] investigated the impact of chamfer and rounded shoulder finish line designs on CAD/CAM-manufactured zirconia copings and found no statistically significant difference. Their study confirmed that the marginal gap values for both designs were within clinically acceptable limits (<120 μm). However, they observed that the lowest internal gap values were associated with the chamfer finish line.

Ferrari et al. [45] compared knife-edge and chamfer margin designs on the same tooth and found that the knife-edge preparation had a lower marginal gap. However, the difference was not statistically significant. Elsherbini et al. [36] found that feather-edge finish line adversely affected both marginal and internal adaptation when compared to the chamfer.

Jalali et al. [44] and Porojan et al. [54] found no significant difference between rounded shoulder and chamfer preparation designs for zirconia crowns. Krasanaki et al. [48] found that chamfer and shoulder preparations had no impact on the marginal gap (MG) or absolute marginal discrepancy (AMD). Mitchell et al. [51] found that the shoulder finish line ensured improved AMD compared with chamfer. Faruqi et al. [39] concluded that chamfer finish lines produced a smaller marginal gap than the shoulder.

### Risk of bias in studies

Of the 50 studies, 12 were assessed as low risk, 31 as medium risk, and 7 as high risk. The main risk factors were lack of information on sample size calculation, operator and outcome assessor details, randomization and blinding. The results of the risk-of-bias assessment are presented in Table 2. The domain-specific results are presented in Supplementary Material 3.

**Table 2:**
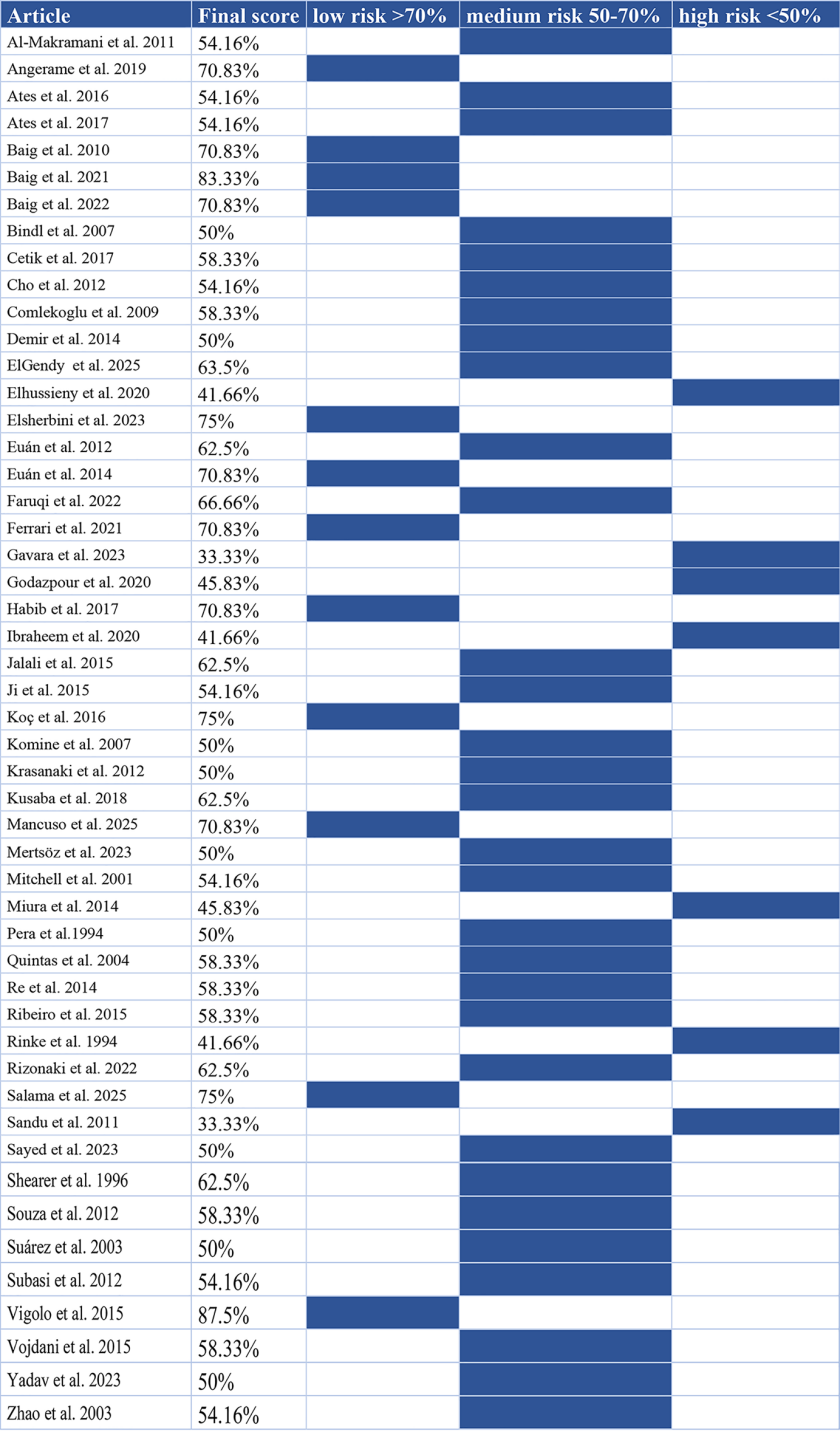
Risk of bias assessment with QUIN tool

### Certainty of evidence

Using the CINeMA tool, the overall quality of evidence was rated as high. According to the GRADEpro assessment, the certainty of evidence was high for the ‘Not cemented absolute marginal discrepancy’ subgroup, but low for both ‘Marginal gap evaluation using the silicon replica technique’ and ‘Internal gap of veneers.’

Major concerns for all outcomes were due to high imprecision, incoherence and heterogeneity. Imprecision was reflected in the wide range of CrIs and the limited number of fixed dental prostheses in certain studies. Additionally, incoherence occurred due to the significant heterogeneity observed in certain methodological and clinical domains across the reported studies. The certainty of the results of the evidence analysis is detailed in Supplementary Material 4.

## Discussion

This systematic review and network meta-analysis aimed at evaluating the effect of different tooth preparation designs on the marginal and internal fit of ceramic fixed dental prostheses. The null hypothesis stated that there was no significant difference between the preparation designs.

The results of this NMA indicate no significant difference in the marginal gap and absolute marginal discrepancy of ceramic fixed dental prostheses between different preparation designs. However, a significant difference in internal adaptation was observed, particularly between rounded shoulder and chamfer finish lines, with a mean difference of 26.65 μm. Given these findings, we failed to reject the null hypothesis for the marginal fit, as the observed differences were not statistically significant. However, the significant difference in internal adaptation allows us to reject the null hypothesis, indicating that chamfer finish lines may be the preferred choice for ceramic fixed dental prostheses.

These findings align with a previous systematic review and meta-analysis comparing chamfer and rounded shoulder finish line designs for ceramic crowns [13]. The authors reported that chamfer finish lines offered superior internal adaptation, with a significant mean difference of 35.0 μm (*P* =.020; 95% CI = 6.5–63.5) favoring chamfer [13]. However, the mean difference in marginal adaptation was only 7.9 μm, a magnitude that is unlikely to be of substantial clinical relevance [13].

Traditionally, in the past decades, the limit of the clinically acceptable marginal gap was considered 120 μm, based on a study by McLean et al. involving over 1,000 fixed restorations evaluated over five years [72]. On the basis of this 120 μm limit, all four preparation designs that we investigated are clinically acceptable for ceramic FDPs. In modern prosthodontics and digital dentistry, these results are significantly surpassed. In an in vitro study, Schwindling et al. reported a marginal misfit ranging from 36 to 88 μm for monolithic full-arch restorations [73]. Nizaroglu et al. reported a mean marginal gap of 60.24–83.66 μm for monolithic and veneered zirconia CAD/CAM crowns [74]. Rizonaki et al. found mean marginal gap values between 23 and 96 μm for lithium disilicate CAD-CAM crowns [59]. CAD/CAM-fabricated restorations generally demonstrate smaller marginal gaps, typically between 50 and 100 μm. Regardless of the preparation design, most studies in this NMA confirmed that CAD/CAM technology consistently achieves precise and reliable marginal gaps within or below this expected range. However, one study presented higher values (152.18–162.25 μm), which the authors attributed to differences in the CAD/CAM system used [42].

The results of this NMA highlight a conflict between marginal and internal fit. For absolute marginal discrepancy of CAD/CAM all-ceramic system, the rounded shoulder showed statistically better results than the vertical (MD: 60.02 μm CrI: 15.56, 106.21) and chamfer preparation (MD: 25.52 μm CrI: 7.61, 50.39). In terms of the internal gap of the CAD/CAM all-ceramic system, chamfer showed significantly better results than rounded shoulder (MD: 31.39 μm CrI: 4.44, 57.19). This discrepancy may be attributed to the cementation process, during which the internal surface moves toward the axial wall, reducing the cement escape path and increasing pressure [75]. This can obstruct proper seating and widen the marginal gap if internal contact occurs prematurely, hindering cement flow [76] and potentially compromising the quality and longevity of the fixed dental prosthesis. Previous research supported the importance of optimizing cement space to improve adaptation of CAD/CAM fixed dental restorations. Zheng et al. emphasized the significant influence of virtual cement space settings on CAD/CAM endocrown restorations, recommending a 60 μm cement space for ceramic materials to ensure even cement distribution while preventing restoration elevation [77]. Similarly, Sultan et al. found that a 60 μm cement space achieved the smallest marginal gap for resin-ceramic crowns on implant abutments [78]. Additionally, Elbadawy et al. concluded that increasing the digital cement space (30, 40, and 50 μm) significantly enhanced the marginal adaptation of IPS e.max-CAD occlusal veneers [79]. These findings suggest that adjusting the cement space is a crucial factor in achieving optimal restoration fit, balancing both marginal and internal adaptation.

In conventional all-ceramic systems, vertical preparation has been shown to achieve statistically superior marginal adaptation compared to chamfer and shoulder preparations. However, for CAD/CAM-fabricated restorations, the rounded shoulder preparation tends to yield the best results in terms of marginal gap, and statistically better results in AMD compared to vertical preparation. This discrepancy arises because most milling systems utilize rounded burs with a minimum diameter of approximately 1 mm, which limits their ability to reproduce sharp or narrow internal angles and fine edge details below this size [80–82]. Consequently, geometries such as vertical or sharply defined margins may not be accurately milled, leading to increased marginal discrepancies. Conversely, these intricate features can still be manually produced by skilled dental technicians using traditional techniques, allowing for greater precision in adapting to fine preparation geometries.

The findings from the subgroup analysis of evaluation techniques reveal contrasting outcomes depending on the assessment method used. Specifically, when marginal gap values were assessed using the direct view technique, the vertical preparation demonstrated significantly smaller marginal gap values compared to other finish line designs such as rounded shoulder, chamfer, and shoulder. However, this trend was not consistent across all evaluation methods. In stark contrast, when the micro-CT technique - a more advanced, three-dimensional, and highly accurate method [7] - was employed, vertical preparation yielded the worst marginal gap values. It is important to note, however, that the number of studies using each technique varied considerably: the group using the direct view technique included 18 studies, whereas only 2 studies utilized the micro-CT technique. This discrepancy in sample size may influence the strength and generalizability of the conclusions drawn. Nevertheless, this divergence underscores a critical methodological consideration: evaluation techniques vary in sensitivity and comprehensiveness [83]. Traditional methods such as direct view technique, cross-sectional microscopy, and silicone replica techniques, although widely used and validated, have limitations in thoroughly assessing gaps and often overlook recommended measurement points. Conversely, 3D digital methods such as the micro-CT technique, Triple-Scan Method, Dual-Scan Method, and Optical Coherence Tomography provide a more thorough and clinically relevant assessment of restoration fit and are recommended for current use [7]. These findings highlight the need for standardized and clinically validated evaluation protocols that leverage the accuracy of modern digital technologies.

### Strengths and limitations

Our analysis has several strengths, including strict adherence to a pre-registered protocol and the application of a rigorous methodology. This was the first systematic review and network meta-analysis to investigate the efficacy of multiple finish line designs on ceramic fixed dental prostheses. We conducted a comprehensive evaluation of various outcomes with both direct and indirect comparisons. One of the strengths of this NMA is that we conducted multiple subgroup analyses to minimize heterogeneity, based on cementation (with and without cementation), manufacturing techniques (CAD/CAM and conventional manufacturing), restoration types (crowns, endocrowns, copings, veneers, occlusal veneers, and small-span bridges), and evaluation techniques (direct view technique, cross-sectioning method, micro-CT, and silicone replica technique). No date or language restrictions were applied.

The authors acknowledge the extensive nature of this study, which covers a broad spectrum of materials, technologies, and processes. Several limitations should be considered when interpreting the findings. The network meta-analysis was affected by population and measurement method heterogeneity and a limited number of studies for specific outcomes, leading to an incomplete network geometry in some domains. Variations in measurement methods across studies may have significantly affected the results. The subgroup analysis by measurement technique is constrained by the limited number of studies available in some categories and should be interpreted with caution, as some findings are derived from as few as one or two studies. It is important to note that in some subgroups (subgroup analysis of small span bridges, AMD of veneers, AMD micro-CT evaluation, AMD silicone replica technique evaluation, and internal gap of conventional all-ceramic system) such analyses could not be performed due to insufficient data. In some subgroups (Not cemented absolute marginal discrepancy, Marginal gap evaluation using the silicon replica technique, and Internal gap of veneers), only pairwise comparisons were possible due to the limited availability of data. In some cases, only three of the available comparators could be included in the evaluation due to insufficient data across the remaining groups (Marginal gap evaluation using the micro-CT technique, Absolute marginal discrepancy evaluation using the direct view technique, Internal gap evaluation using the silicone replica technique, Internal gap evaluation using the micro-CT technique, Marginal gap of copings, Marginal gap of veneers, Absolute marginal discrepancy of copings, and Internal gap of copings). The limited number of studies and inconsistent reporting within certain categories restricted our ability to draw meaningful comparisons or conclusions for specific subgroups. This limitation highlights the need for more comprehensive and standardized reporting in future research to enable more robust subgroup evaluations. Furthermore, the lack of analysis on the effect of varying cement space values posed an additional limitation, as no further subgroups were created based on this factor.

Additionally, there was a moderate to high risk of bias in some domains, primarily due to lack of information on operator experience, which, the authors suggest, is a key factor influencing accuracy in dental preparation. The absence of details on the outcome assessor and the statistical program used further contributed to the risk of bias.

Although the study provides a valuable synthesis of current evidence, its limitations reflect the complexity of the topic and the need for ongoing refinement in research approaches.

### Implications for practice

The rapid application of scientific results is critically essential [84, 85]. The findings collected from this NMA inform dentists that all finish line designs produced clinically acceptable results. Therefore, dentists should select the preparation design based on the specific clinical situation and tooth anatomy, including factors such as tooth morphology, enamel thickness, and the position of the tooth in the dental arch [86, 87]. Horizontal preparations are widely accepted as the standard approach for tooth preparation. On the other hand, minimally invasive vertical preparations should be considered for teeth that require tooth preservation, such as tilted teeth or smaller lower incisors [87]. This technique is ideal when the preservation of as much natural tooth structure as possible is a priority, especially when esthetics and minimal tooth reduction are key factors [87].

A systematic review and meta-analysis found that dental restorations showed comparable outcomes in terms of longevity and effectiveness, regardless of whether horizontal or vertical preparation methods were used [86].

According to the results of the current NMA, the chamfer finish line is specifically favored for ceramic fixed dental prostheses for the best overall performance and minimal invasiveness.

### Implications for research

Future studies should compare different preparation designs using standardized methodologies and consistent measuring points. Micro-CT is recommended for its high accuracy and spatial resolution. Additionally, establishing standardized and accurate thresholds for marginal and internal gaps is essential for meaningful comparisons. A strict limit should be applied for prosthodontic purposes. We recommend that future studies employ clear and well-defined comparators, with more uniform and standardized study designs to reduce heterogeneity and improve comparability.

## Conclusions

The following conclusions were drawn based on the results of this NMA and within its limitations:

This network meta-analysis identified the **chamfer** as the most effective preparation design for minimizing the internal gap, **vertical preparation** as the most effective for reducing the marginal gap, and the **rounded shoulder** as the optimal design for minimizing the absolute marginal discrepancy.

Although no statistically significant differences in marginal fit (MG and AMD) were found between the tested designs (**vertical**,** chamfer**,** rounded shoulder**,** and shoulder**), the superior internal adaptation (IG) observed with the **chamfer** finish line supports its clinical recommendation.

Given the conflicting results between internal and marginal fit, optimizing cement space remains essential to achieve a well-balanced adaptation, thereby enhancing the longevity and clinical success of ceramic fixed dental prostheses.

## Electronic supplementary material

Below is the link to the electronic supplementary material.

Supplementary Material 1

Supplementary Material 2

Supplementary Material 3

Supplementary Material 4

Supplementary Material 5

Supplementary Material 6

Supplementary Material 7

## Data Availability

The datasets utilized in this study can be found in the full-text articles included in the systematic review and meta-analysis.

## Abbreviations

NMA: Network meta-analysis
FDP: Fixed dental prostheses
MG: Marginal gap
IG: Internal gap
AMD: Absolute marginal discrepancy
CAD/CAM: Computer-aided design and computer-aided manufacturing
SUCRA: The surface under cumulative ranking
micro-CT: Microcomputed X-ray tomography
95% CrI: 95% credible intervals
MD: Mean difference

## References

[1] Giordano Ii R. Ceramics overview. Br Dent J. 2022;232(9):658–63. 35562468 10.1038/s41415-022-4242-6

[2] Jurado CA, Davila CE, Davila A, Hernandez AI, Odagiri Y, Afrashtehfar KI, Lee D. Influence of occlusal thickness on the fracture resistance of chairside milled lithium disilicate posterior full-coverage single-unit prostheses containing virgilite: A comparative in vitro study. J Prosthodont 2024. 10.1111/jopr.13870 PMC12541292 38790151

[3] Warreth A, Elkareimi Y. All-ceramic restorations: A review of the literature. Saudi Dent J. 2020;32(8):365–72. 34588757 10.1016/j.sdentj.2020.05.004 PMC8461086

[4] Mously HA, Finkelman M, Zandparsa R, Hirayama H. Marginal and internal adaptation of ceramic crown restorations fabricated with CAD/CAM technology and the heat-press technique. J Prosthet Dent. 2014;112(2):249–56. 24795263 10.1016/j.prosdent.2014.03.017

[5] Contrepois M, Soenen A, Bartala M, Laviole O. Marginal adaptation of ceramic crowns: a systematic review. J Prosthet Dent. 2013;110(6):447–e454410. 24120071 10.1016/j.prosdent.2013.08.003

[6] Holmes JR, Bayne SC, Holland GA, Sulik WD. Considerations in measurement of marginal fit. J Prosthet Dent. 1989;62(4):405–8. 2685240 10.1016/0022-3913(89)90170-4

[7] Ayres AP, Cuschieri LA, Bianchi DM, Pradíes G, Côrtes ARG. Advantages and drawbacks of different methods to measure marginal gaps in fixed dental prostheses: A scoping review. J Dent. 2024;151:105400. 39393607 10.1016/j.jdent.2024.105400

[8] Ahmed WM, Shariati B, Gazzaz AZ, Sayed ME, Carvalho RM. Fit of tooth-supported zirconia single crowns-A systematic review of the literature. Clin Exp Dent Res. 2020;6(6):700–16. 32885613 10.1002/cre2.323 PMC7745068

[9] Papadiochou S, Pissiotis AL. Marginal adaptation and CAD-CAM technology: A systematic review of restorative material and fabrication techniques. J Prosthet Dent. 2018;119(4):545–51. 28967399 10.1016/j.prosdent.2017.07.001

[10] Loi I, Di Felice A. Biologically oriented Preparation technique (BOPT): a new approach for prosthetic restoration of periodontically healthy teeth. Eur J Esthet Dent. 2013;8(1):10–23. 23390618

[11] Lawand G, Ajili A, Ismail Y. Biologically Oriented Preparation Technique (BOPT). In: *Innovative Perspectives in Oral and Maxillofacial Surgery.* edn. Edited by Stevens MR, Ghasemi S, Tabrizi R. Cham: Springer International Publishing; 2021: 175–194.

[12] Borelli B, Sorrentino R, Goracci C, Zarone F, Ferrari M. In vitro analysis of residual tooth structure of maxillary anterior teeth after different prosthetic finish line preparations for full-coverage single crowns. J Oral Sci. 2013;55(1):79–84. 23485605 10.2334/josnusd.55.79

[13] Yu H, Chen YH, Cheng H, Sawase T. Finish-line designs for ceramic crowns: A systematic review and meta-analysis. J Prosthet Dent. 2019;122(1):22–e3025. 30782459 10.1016/j.prosdent.2018.10.002

[14] Kasem AT, Ellayeh M, Özcan M, Sakrana AA. Three-year clinical evaluation of zirconia and zirconia-reinforced lithium silicate crowns with minimally invasive vertical Preparation technique. Clin Oral Investig. 2023;27(4):1577–88. 36383297 10.1007/s00784-022-04779-1 PMC10102102

[15] Mohammad A, Abraham S, Nada A. The effect of biologically oriented and subgingival horizontal Preparation techniques on periodontal health: A double-blind randomized controlled clinical trial. Saudi Dent J. 2023;35(6):727–33. 37817795 10.1016/j.sdentj.2023.06.003 PMC10562095

[16] Hermann P, Kispélyi B. Fogpótlástan: Semmelweis Kiadó; 2022.

[17] Page MJ, McKenzie JE, Bossuyt PM, Boutron I, Hoffmann TC, Mulrow CD, Shamseer L, Tetzlaff JM, Akl EA, Brennan SE, et al. The PRISMA 2020 statement: an updated guideline for reporting systematic reviews. BMJ. 2021;372:n71. 33782057 10.1136/bmj.n71 PMC8005924

[18] Ouzzani M, Hammady H, Fedorowicz Z, Elmagarmid A. Rayyan—a web and mobile app for systematic reviews. Syst Reviews 2016; 5. 10.1186/s13643-016-0384-4 PMC5139140 27919275

[19] Sheth VH, Shah NP, Jain R, Bhanushali N, Bhatnagar V. Development and validation of a risk-of-bias tool for assessing in vitro studies conducted in dentistry: the QUIN. J Prosthet Dent 2022. 10.1016/j.prosdent.2022.05.019 35752496

[20] GRADE-PRO website. [https://www.gradepro.org/]

[21] Nikolakopoulou A, Higgins JPT, Papakonstantinou T, Chaimani A, Del Giovane C, Egger M, Salanti G. CINeMA: an approach for assessing confidence in the results of a network meta-analysis. PLoS Med. 2020;17(4):e1003082. 32243458 10.1371/journal.pmed.1003082 PMC7122720

[22] Ahmadzadeh A, Godazpour R, Jafarizadeh A. Comparison of shoulder & chamfer finish line designs on marginal adaptation of IPS e. 2020.

[23] Al-Makramani BM, Razak AA, Abu-Hassan MI, Sulaiman E, Loon LJ, Yahya NA. Marginal integrity of turkom-cera compared to other all-ceramic materials: effect of finish line. Int J Prosthodont. 2011;24(4):379–81. 21716978

[24] Angerame D, De Biasi M, Agostinetto M, Franzò A, Marchesi G. Influence of Preparation designs on marginal adaptation and failure load of full-coverage occlusal veneers after thermomechanical aging simulation. J Esthet Restor Dent. 2019;31(3):280–9. 30790399 10.1111/jerd.12457

[25] Ates SM, Yesil Duymus Z. Influence of tooth Preparation design on fitting accuracy of CAD-CAM based restorations. J Esthet Restor Dent. 2016;28(4):238–46. 27061751 10.1111/jerd.12208

[26] Ates SM, Yesil Duymus Z, Caglar I, Hologlu B. The effect of veneering on the marginal fit of CAD/CAM-generated, copy-milled, and cast metal copings. Clin Oral Investig. 2017;21(8):2553–60. 28091875 10.1007/s00784-017-2054-x

[27] Baig MR, Akbar AA, Embaireeg M. Effect of Finish Line Design on the Fit Accuracy of CAD/CAM Monolithic Polymer-Infiltrated Ceramic-Network Fixed Dental Prostheses: An In Vitro Study. Polymers (Basel) 2021; 13(24). 10.3390/polym13244311 PMC8705895 34960861

[28] Baig MR, Al-Tarakemah Y, Kasim NHA, Omar R. Evaluation of the marginal fit of a CAD/CAM zirconia-based ceramic crown system. Int J Prosthodont. 2022;35(3):319–29. 33616567 10.11607/ijp.6654

[29] Baig MR, Tan KB, Nicholls JI. Evaluation of the marginal fit of a zirconia ceramic computer-aided machined (CAM) crown system. J Prosthet Dent. 2010;104(4):216–27. 20875526 10.1016/S0022-3913(10)60128-X

[30] Bindl A, Mörmann WH. Fit of all-ceramic posterior fixed partial denture frameworks in vitro. Int J Periodontics Restor Dent. 2007;27(6):567–75. 18092451

[31] Cetik S, Bahrami B, Fossoyeux I, Atash R. Adaptation of zirconia crowns created by conventional versus optical impression: in vitro study. J Adv Prosthodont. 2017;9(3):208–16. 28680553 10.4047/jap.2017.9.3.208 PMC5483408

[32] Cho SH, Nagy WW, Goodman JT, Solomon E, Koike M. The effect of multiple firings on the marginal integrity of pressable ceramic single crowns. J Prosthet Dent. 2012;107(1):17–23. 22230912 10.1016/S0022-3913(12)60011-0

[33] Comlekoglu M, Dundar M, Ozcan M, Gungor M, Gokce B, Artunc C. Influence of cervical finish line type on the marginal adaptation of zirconia ceramic crowns. Oper Dent. 2009;34(5):586–92. 19830974 10.2341/08-076-L

[34] Demir N, Ozturk AN, Malkoc MA. Evaluation of the marginal fit of full ceramic crowns by the microcomputed tomography (micro-CT) technique. Eur J Dent. 2014;8(4):437–44. 25512721 10.4103/1305-7456.143612 PMC4253096

[35] Elhussieny M, Ismail M, Mohsen CA. Aging effect on marginal gap distance and Cyclic loading of two different ceramic crowns. Indian J Public Health Res Dev. 2020;11(3):1869–73.

[36] Elsherbini M, Sakrana AA, Amin RA, Diaa M, Özcan M, Al-Zordk W. A micro-computed tomography analysis of internal and marginal fits of fixed partial dentures: effect of Preparation finish line designs on monolithic zirconia and heat-pressed zirconia-reinforced lithium disilicate. J Prosthodont. 2023;32(5):e90–9. 10.1111/jopr.13656 36718906

[37] Euán R, Figueras-Álvarez O, Cabratosa-Termes J, Brufau-de Barberà M, Gomes-Azevedo S. Comparison of the marginal adaptation of zirconium dioxide crowns in preparations with two different finish lines. J Prosthodont. 2012;21(4):291–5. 22372886 10.1111/j.1532-849X.2011.00831.x

[38] Euán R, Figueras-Álvarez O, Cabratosa-Termes J, Oliver-Parra R. Marginal adaptation of zirconium dioxide copings: influence of the CAD/CAM system and the finish line design. J Prosthet Dent. 2014;112(2):155–62. 24445027 10.1016/j.prosdent.2013.10.012

[39] Faruqi S, Ganji KK, Bandela V, Nagarajappa AK, Mohamed RN, Ahmed MA, Farhan M, Alwakid WN, Al-Hammad KAS, Alam MK. Digital assessment of marginal accuracy in ceramic crowns fabricated with different marginal finish line configurations. J Esthet Restor Dent. 2022;34(5):789–95. 34668306 10.1111/jerd.12822

[40] Ferrari M, Marucci A, Cagidiaco EF, Pontoriero DI, Fuzzi M. Sealing ability of new translucent zirconia crowns made with digital workflow and cemented with different types of cement. Int J Periodontics Restor Dent. 2021;41(5):703–10. 10.11607/prd.4973 34547074

[41] Gavara SG, Jain S, Gupta H, Sharma S, Panwar P, Momin MS. Comparative effect of no finish line, heavy chamfer, and shoulder marginal designs on the fracture resistance of zirconia (Cercon) ceramic restoration: an in vitro study. Cureus. 2023;15(5):e39009. 37323304 10.7759/cureus.39009 PMC10264088

[42] Habib SR, Al Ajmi MG, Al Dhafyan M, Jomah A, Abualsaud H, Almashali M. Effect of margin designs on the marginal adaptation of zirconia copings. Acta Stomatol Croat. 2017;51(3):179–87. 29225358 10.15644/asc51/3/1 PMC5708331

[43] Ibraheem A, Abdullah L. Evaluation of post cementation marginal seating of monolithic zirconia crown restorations using different Preparation designs (A comparative in vitro study). Indian J Forensic Med Toxicol. 2020;14:787.

[44] Jalali H, Sadighpour L, Miri A, Shamshiri AR. Comparison of marginal fit and fracture strength of a CAD/CAM zirconia crown with two Preparation designs. J Dent (Tehran). 2015;12(12):874–81. 27559346 PMC4983302

[45] Ji MK, Park JH, Park SW, Yun KD, Oh GJ, Lim HP. Evaluation of marginal fit of 2 CAD-CAM anatomic contour zirconia crown systems and lithium disilicate glass-ceramic crown. J Adv Prosthodont. 2015;7(4):271–7. 26330973 10.4047/jap.2015.7.4.271 PMC4551782

[46] Koç E, Öngül D, Şermet B. A comparative study of marginal fit of copings prepared with various techniques on different implant abutments. Dent Mater J. 2016;35(3):447–53. 27252001 10.4012/dmj.2015-252

[47] Komine F, Iwai T, Kobayashi K, Matsumura H. Marginal and internal adaptation of zirconium dioxide ceramic copings and crowns with different finish line designs. Dent Mater J. 2007;26(5):659–64. 18203465 10.4012/dmj.26.659

[48] Krasanaki ME, Pelekanos S, Andreiotelli M, Koutayas SO, Eliades G. X-ray microtomographic evaluation of the influence of two Preparation types on marginal fit of CAD/CAM alumina copings: a pilot study. Int J Prosthodont. 2012;25(2):170–2. 22371840

[49] Kusaba K, Komine F, Honda J, Kubochi K, Matsumura H. Effect of Preparation design on marginal and internal adaptation of translucent zirconia laminate veneers. Eur J Oral Sci. 2018;126(6):507–11. 30289591 10.1111/eos.12574

[50] Mertsöz B, Ongun S, Ulusoy M. In-Vitro investigation of marginal adaptation and fracture resistance of resin matrix ceramic Endo-Crown restorations. Materials. 2023;16(5):2059. 36903174 10.3390/ma16052059 PMC10004208

[51] Mitchell CA, Pintado MR, Douglas WH. Nondestructive, in vitro quantification of crown margins. J Prosthet Dent. 2001;85(6):575–84. 11404758 10.1067/mpr.2001.114268

[52] Miura S, Inagaki R, Kasahara S, Yoda M. Fit of zirconia all-ceramic crowns with different cervical margin designs, before and after porcelain firing and glazing. Dent Mater J. 2014;33(4):484–9. 24988882 10.4012/dmj.2013-284

[53] Pera P, Gilodi S, Bassi F, Carossa S. In vitro marginal adaptation of alumina porcelain ceramic crowns. J Prosthet Dent. 1994;72(6):585–90. 7853254 10.1016/0022-3913(94)90289-5

[54] Porojan L, Topala F, Porojan S. Marginal design evaluation for CAM obtained zirconia based crown frameworks. Adv Mater Res. 2011;213:349–53.

[55] Quintas AF, Oliveira F, Bottino MA. Vertical marginal discrepancy of ceramic copings with different ceramic materials, finish lines, and Luting agents: an in vitro evaluation. J Prosthet Dent. 2004;92(3):250–7. 15343160 10.1016/j.prosdent.2004.06.023

[56] Re D, Cerutti F, Augusti G, Cerutti A, Augusti D. Comparison of marginal fit of lava CAD/CAM crown-copings with two finish lines. Int J Esthet Dent. 2014;9(3):426–35. 25126621

[57] Ribeiro IL, Campos F, Sousa RS, Alves ML, Rodrigues DM, Souza RO, Bottino MA. Marginal and internal discrepancies of zirconia copings: effects of milling system and finish line design. Indian J Dent Res. 2015;26(1):15–20. 25961609 10.4103/0970-9290.156790

[58] Rinke S, Margraf G, Jahn L, Hüls A. [The quality appraisal of copy-milled complete-ceramic crown structures (Celay/In-Ceram)]. Schweiz Monatsschr Zahnmed. 1994;104(12):1495–9. 7824900

[59] Rizonaki M, Jacquet W, Bottenberg P, Depla L, Boone M, De Coster PJ. Evaluation of marginal and internal fit of lithium disilicate CAD-CAM crowns with different finish lines by using a micro-CT technique. J Prosthet Dent. 2022;127(6):890–8. 33541816 10.1016/j.prosdent.2020.11.027

[60] Sayed O, Elbolok A, Amgad S. Assessment of internnal adaptation of cad cam all ceramic crowns with two different margin designs. Egypt Dent J. 2023;69(1):583–92.

[61] Shearer B, Gough MB, Setchell DJ. Influence of marginal configuration and porcelain addition on the fit of In-Ceram crowns. Biomaterials. 1996;17(19):1891–5. 8889069 10.1016/0142-9612(95)00302-9

[62] Souza RO, Özcan M, Pavanelli CA, Buso L, Lombardo GH, Michida SM, Mesquita AM, Bottino MA. Marginal and internal discrepancies related to margin design of ceramic crowns fabricated by a CAD/CAM system. J Prosthodont. 2012;21(2):94–100. 22050205 10.1111/j.1532-849X.2011.00793.x

[63] Suárez MJ, González de Villaumbrosia P, Pradíes G, Lozano JF. Comparison of the marginal fit of procera allceram crowns with two finish lines. Int J Prosthodont. 2003;16(3):229–32. 12854783

[64] Subasi G, Ozturk N, Inan O, Bozogullari N. Evaluation of marginal fit of two all-ceramic copings with two finish lines. Eur J Dent. 2012;6(2):163–8. 22509119 PMC3327498

[65] Vigolo P, Mutinelli S, Biscaro L, Stellini E. An in vivo evaluation of the fit of Zirconium-Oxide based, ceramic single crowns with vertical and horizontal finish line preparations. J Prosthodont. 2015;24(8):603–9. 26359654 10.1111/jopr.12340

[66] Vojdani M, Safari A, Mohaghegh M, Pardis S, Mahdavi F. The effect of porcelain firing and type of finish line on the marginal fit of zirconia copings. J Dent (Shiraz). 2015;16(2):113–20. 26046107 PMC4445849

[67] Yadav P, Sharma V, Paliwal J, Meena KK, Madaan R, Gurjar B. An in vitro comparison of zirconia and hybrid ceramic crowns with heavy chamfer and shoulder finish lines. Cureus. 2023;15(1):e33940. 36819334 10.7759/cureus.33940 PMC9937780

[68] Zhao YF, Wang HR, Li Y. [The effect of tooth Preparation design on the CAD/CAM all-ceramic coping crown’s fitness]. Zhonghua Kou Qiang Yi Xue Za Zhi. 2003;38(5):330–2. 14680576

[69] ElGendy M, Sherif R, Rabie K. Internal fit and marginal adaptation of posterior CAD/CAM crowns fabricated from fully crystallized Lithium disilicate compared to partially crystallized Lithium disilicate with two finish line thicknesses: an in vitro study. J Contemp Dent Pract. 2024;25(8):740–4. 39653665 10.5005/jp-journals-10024-3744

[70] Mancuso E, Forte A, Maravic T, Mazzitelli C, Comba A, Baldi A, Fehmer V, Sailer I, Scotti N, Mazzoni A et al. Effects of Preparation design on the marginal and internal fit of CAD-CAM overlay restorations: A µCT evaluation. J Prosthet Dent 2025. 10.1016/j.prosdent.2024.12.023 39894697

[71] Salama MA, Aldamaty MF, Abdalla MA, Omar EA, AbdElaziz MH, Alqutaibi AY. Marginal fit and fracture resistance of vertical versus horizontal margins in monolithic zirconia crowns. Clin Exp Dent Res. 2025;11(1):e70064. 39853710 10.1002/cre2.70064 PMC11757025

[72] McLean JW, von Fraunhofer JA. The Estimation of cement film thickness by an in vivo technique. Br Dent J. 1971;131(3):107–11. 5283545 10.1038/sj.bdj.4802708

[73] Schwindling FS, Bechtel KN, Zenthöfer A, Handermann R, Rammelsberg P, Rues S. In-vitro fit of experimental full-arch restorations made from monolithic zirconia. J Prosthodont Res. 2022;66(2):258–64. 34305088 10.2186/jpr.JPR_D_20_00321

[74] Temizkan Nizaroglu R, Küçük C. Evaluation of marginal and internal adaptation of crowns fabricated with three different zirconia CAD/CAM materials. Niger J Clin Pract 2024, 27(1). 10.4103/njcp.njcp_410_23 38317035

[75] Gavelis JR, Morency JD, Riley ED, Sozio RB. The effect of various finish line preparations on the marginal seal and occlusal seat of full crown preparations. J Prosthet Dent. 1981;45(2):138–45. 7009833 10.1016/0022-3913(81)90330-9

[76] Hmaidouch R, Neumann P, Mueller W-D. Influence of Preparation form, Luting space setting and cement type on the marginal and internal fit of CAD/CAM crown copings. Int J Comput Dent. 2011;14:219–26. 22141231

[77] Zheng Z, Wang H, Mo J, Ling Z, Zeng Y, Zhang Y, Wang J, Yan W. Effect of virtual cement space and restorative materials on the adaptation of CAD-CAM endocrowns. BMC Oral Health. 2022;22(1):580. 36494663 10.1186/s12903-022-02598-0 PMC9733092

[78] Sultan S, Hegazy M, Shakal M, Magdy S. Effect of virtual cement gap settings on the marginal fit of cemented resin-ceramic crowns on implant abutments. J Prosthet Dent. 2021;125(5):e804801–6. 10.1016/j.prosdent.2021.02.014 33934821

[79] Elbadawy AA, Elaziz MHA, Alnazzawi AA, Borzangy SS. Effect of various digital cement space settings on the adaptation of CAD/CAM occlusal veneer micro-ct evaluation. Dent Mater J. 2021;40(3):625–30. 33390385 10.4012/dmj.2020-226

[80] Zuskova L, Mortadi NAA, Williams RJ, Alzoubi KH, Khabour OF. Comparison of Overall Fit of Milled and Laser-Sintered CAD/CAM Crown Copings. *Int J Dent* 2019, 2019:7310175. 10.1155/2019/7310175 PMC6642757 31360167

[81] Giannetopoulos S, van Noort R, Tsitrou E. Evaluation of the marginal integrity of ceramic copings with different marginal angles using two different CAD/CAM systems. J Dent. 2010;38(12):980–6. 20736043 10.1016/j.jdent.2010.08.011

[82] Turkyilmaz I, Wilkins GN, Yun S. Moving from analogue to digital workflows in dentistry: Understanding undermilling and overmilling as detrimental factors in fabricating CAD/CAM crowns. Prim Dent J. 2022;11(2):59–61. 35658655 10.1177/20501684221100938

[83] Di Fiore A, Zuccon A, Carraro F, Basilicata M, Bollero P, Bruno G, Stellini E. Assessment methods for marginal and internal fit of partial crown restorations: A systematic review. J Clin Med 2023; 12(15). 10.3390/jcm12155048 PMC10419640 37568450

[84] Hegyi P, Erőss B, Izbéki F, Párniczky A, Szentesi A. Accelerating the translational medicine cycle: the academia Europaea pilot. Nat Med. 2021;27(8):1317–9. 34312557 10.1038/s41591-021-01458-8

[85] Hegyi P, Petersen OH, Holgate S, Erőss B, Garami A, Szakács Z, Dobszai D, Balaskó M, Kemény L, Peng S et al. Academia Europaea position paper on translational medicine: the cycle model for translating scientific results into community benefits. J Clin Med 2020; 9(5). 10.3390/jcm9051532 PMC7290380 32438747

[86] Bonfanti-Gris M, Pradies G, Moron-Conejo B, Gil A, Martinez-Rus F. Vertical versus horizontal finishing lines for dental preparations: A systematic review with Meta-Analysis. J Esthet Restor Dent 2024. 10.1111/jerd.13360 PMC12076104 39620436

[87] Łabno P, Drobnik K. Comparison of horizontal and vertical methods of tooth Preparation for a prosthetic crown. J Pre Clin Clin Res 2020; 14.

