# Supplementary material for: Impact of finish line designs on the adaptation of ceramic fixed dental prostheses: a systematic review and network meta-analysis
Source: BMC Oral Health. 2025 Jul 3;25:1085. doi: 10.1186/s12903-025-06433-0 (PMC12231902; doi:10.1186/s12903-025-06433-0)
Supplement: Supplementary file 2 — Supplementary Material 2 [file 12903_2025_6433_MOESM2_ESM.docx]

**Search keys:**

Throughout the systematic search, the following search key was used for Pubmed: (ceram* OR zircon* OR „lithium" OR „disilicate" OR alumin*) AND ((„finish” AND „line”) OR „shoulder” OR „chamfer” OR „edge” OR „knife” OR „feather”) AND (marginal OR fit OR gap OR internal OR discrepancy OR adaptation)

The following terms were used in the Embase search: (ceram* OR zircon* OR lithium OR disilicate OR alumin*) AND ((finish AND line) OR shoulder OR chamfer OR edge OR knife OR feather) AND (marginal OR fit OR gap OR internal OR discrepancy OR adaptation).

The subsequent terms were used in the Web of Science search: (ceram* OR zircon* OR "lithium" OR "disilicate" OR alumin*) AND (("finish" AND "line") OR "shoulder" OR "chamfer" OR "edge" OR "knife" OR "feather") AND ("marginal" OR "fit" OR "gap" OR "internal" OR "discrepancy" OR "adaptation")

The terms listed below were utilized in the Cochrane search: (ceram* OR zircon* OR "lithium" OR "disilicate" OR alumin*) AND (("finish" AND "line") OR "shoulder" OR "chamfer" OR "edge" OR "knife" OR "feather") AND (marginal OR fit OR gap OR internal OR discrepancy OR adaptation)

Additionally, the reference lists of articles deemed relevant were examined to ensure comprehensive coverage.
