# Supplementary material for: Impact of finish line designs on the adaptation of ceramic fixed dental prostheses: a systematic review and network meta-analysis
Source: BMC Oral Health. 2025 Jul 3;25:1085. doi: 10.1186/s12903-025-06433-0 (PMC12231902; doi:10.1186/s12903-025-06433-0)
Supplement: Supplementary file 3 — Supplementary Material 3 [file 12903_2025_6433_MOESM3_ESM.docx]

**Quality assessment tool for in vitro studies:** QUIN Tool

| Study | Clearly stated aims/objectives | Detailed explanation of sample size calculation | Detailed explanation of sampling technique | Details of comparison group | Detailed explanation of methodology | Operator details | Randomization | Method of measurement of outcome | Outcome assessor details | Blinding | Statistical analysis | Presentation of results | SCORE | Bias Evaluation∗ |
| --- | --- | --- | --- | --- | --- | --- | --- | --- | --- | --- | --- | --- | --- | --- |
| Al-Makramani et al. 2011 (1) | 2 | 0 | 1 | 2 | 2 | 0 | 1 | 2 | 0 | 0 | 1 | 2 | 13 | 54,16: medium risk of bias |
| Angerame et al. 2019 (2) | 2 | 2 | 2 | 2 | 2 | 1 | 1 | 2 | 0 | 0 | 2 | 1 | 17 | 70,83: low risk of bias |
| Ates et al. 2016 (3) | 2 | 0 | 1 | 2 | 2 | 0 | 0 | 2 | 0 | 0 | 2 | 2 | 13 | 54,16: medium risk of bias |
| Ates et al. 2017 (4) | 2 | 0 | 1 | 2 | 2 | 0 | 0 | 2 | 0 | 0 | 2 | 2 | 13 | 54,16: medium risk of bias |
| Baig et al. 2010 (5) | 2 | 1 | 1 | 2 | 2 | 1 | 0 | 2 | 2 | 0 | 2 | 2 | 17 | 70,83: low risk of bias |
| Baig et al. 2021 (6) | 2 | 2 | 2 | 2 | 2 | 0 | 2 | 2 | 2 | 0 | 2 | 2 | 20 | 83,33: low risk of bias |
| Baig et al. 2022 (7) | 2 | 1 | 1 | 2 | 2 | 1 | 1 | 2 | 2 | 0 | 1 | 2 | 17 | 70,83: low risk of bias |
| Bindl et al. 2007 (8) | 2 | 0 | 1 | 2 | 2 | 0 | 0 | 2 | 0 | 0 | 2 | 1 | 12 | 50: medium risk of bias |
| Cetik et al. 2017 (9) | 2 | 0 | 1 | 2 | 2 | 0 | 0 | 2 | 1 | 0 | 2 | 2 | 14 | 58,33: medium risk of bias |
| Cho et al. 2012 (10) | 2 | 0 | 1 | 2 | 2 | 1 | 0 | 2 | 0 | 0 | 1 | 2 | 13 | 54,16: medium risk of bias |
| Comlekoglu et al. 2009 (11) | 2 | 0 | 1 | 2 | 2 | 1 | 0 | 2 | 1 | 0 | 2 | 1 | 14 | 58,33: medium risk of bias |
| Demir et al. 2014 (12) | 2 | 0 | 1 | 2 | 2 | 1 | 0 | 2 | 0 | 0 | 2 | 2 | 14 | 50: medium risk of bias |
| Elhussieny et al. 2020 (13) | 2 | 0 | 1 | 2 | 2 | 0 | 0 | 1 | 0 | 0 | 0 | 2 | 10 | 41,66: high risk of bias |
| Elsherbini et al. 2023 (14) | 2 | 2 | 2 | 2 | 2 | 1 | 2 | 2 | 0 | 0 | 1 | 2 | 18 | 75: low risk of bias |
| Euán et al. 2012 (15) | 2 | 0 | 1 | 2 | 2 | 1 | 1 | 2 | 0 | 0 | 2 | 2 | 15 | 62,5: medium risk of bias |
| Euán et al. 2014 (16) | 2 | 2 | 2 | 2 | 2 | 1 | 0 | 2 | 0 | 0 | 2 | 2 | 17 | 70,83: low risk of bias |
| Faruqi et al. 2022 (17) | 2 | 2 | 2 | 2 | 2 | 1 | 0 | 2 | 0 | 0 | 1 | 2 | 16 | 66,66: medium risk of bias |
| Ferrari et al. 2021 (18) | 2 | 0 | 1 | 2 | 2 | 1 | 1 | 2 | 1 | 2 | 1 | 2 | 17 | 70,83: low risk of bias |
| Gavara et al. 2023 (19) | 2 | 0 | 1 | 2 | 1 | 0 | 0 | 1 | 0 | 0 | 0 | 1 | 8 | 33,33: high risk of bias |
| Godazpour et al. 2020 (20) | 2 | 0 | 1 | 2 | 1 | 0 | 0 | 1 | 0 | 0 | 2 | 2 | 11 | 45,83: high risk of bias |
| Habib et al. 2017 (21) | 2 | 0 | 1 | 2 | 2 | 1 | 2 | 2 | 1 | 0 | 2 | 2 | 17 | 70,83: low risk of bias |
| Ibraheem et al. 2020 (22) | 1 | 0 | 1 | 2 | 2 | 0 | 0 | 2 | 0 | 0 | 1 | 1 | 10 | 41,66: high risk of bias |
| Jalali et al. 2015 (23) | 2 | 0 | 2 | 2 | 2 | 0 | 1 | 2 | 0 | 0 | 2 | 2 | 15 | 62,5: medium risk of bias |
| Ji et al. 2015 (24) | 2 | 0 | 1 | 2 | 2 | 0 | 0 | 2 | 0 | 0 | 2 | 2 | 13 | 54,16: medium risk of bias |
| Koç et al. 2016 (25) | 2 | 2 | 2 | 2 | 2 | 1 | 0 | 2 | 1 | 0 | 2 | 2 | 18 | 75: low risk of bias |
| Komine et al. 2007 (26) | 2 | 0 | 1 | 2 | 2 | 0 | 0 | 2 | 1 | 0 | 1 | 1 | 12 | 50: medium risk of bias |
| Krasanaki et al. 2012 (27) | 2 | 0 | 1 | 2 | 2 | 0 | 0 | 2 | 0 | 0 | 2 | 1 | 12 | 50: medium risk of bias |
| Kusaba et al. 2018 (28) | 2 | 2 | 2 | 2 | 2 | 0 | 0 | 2 | 0 | 0 | 1 | 2 | 15 | 62,5: medium risk of bias |
| Mertsöz et al. 2023 (29) | 2 | 0 | 1 | 2 | 2 | 0 | 0 | 2 | 0 | 0 | 1 | 2 | 12 | 50: medium risk of bias |
| Mitchell et al. 2001 (30) | 2 | 0 | 1 | 2 | 2 | 0 | 1 | 2 | 0 | 0 | 1 | 2 | 13 | 54,16: medium risk of bias |
| Miura et al. 2014 (31) | 2 | 0 | 1 | 2 | 2 | 0 | 0 | 2 | 0 | 0 | 1 | 1 | 11 | 45,83: high risk of bias |
| Pera et al.1994 (32) | 2 | 0 | 1 | 2 | 2 | 0 | 0 | 2 | 0 | 0 | 1 | 2 | 12 | 50: medium risk of bias |
| Quintas et al. 2004 (33) | 2 | 0 | 1 | 2 | 2 | 0 | 0 | 2 | 1 | 0 | 2 | 2 | 14 | 58,33: medium risk of bias |
| Re et al. 2014 (34) | 2 | 0 | 1 | 2 | 2 | 0 | 0 | 2 | 1 | 0 | 2 | 2 | 14 | 58,33: medium risk of bias |
| Ribeiro et al. 2015 (35) | 2 | 0 | 1 | 2 | 2 | 0 | 0 | 2 | 1 | 0 | 2 | 2 | 14 | 58,33: medium risk of bias |
| Rinke et al. 1994 (36) | 1 | 1 | 1 | 2 | 1 | 0 | 0 | 2 | 0 | 0 | 1 | 1 | 10 | 41,66: high risk of bias |
| Rizonaki et al. 2022 (37) | 2 | 0 | 1 | 2 | 2 | 1 | 0 | 2 | 1 | 0 | 2 | 2 | 15 | 62,5: medium risk of bias |
| Sandu et al. 2011 (38) | 2 | 0 | 1 | 2 | 1 | 0 | 0 | 0 | 0 | 0 | 0 | 2 | 8 | 33,33: high risk of bias |
| Sayed et al. 2023 (39) | 1 | 0 | 1 | 2 | 2 | 1 | 0 | 2 | 0 | 0 | 1 | 2 | 12 | 50: medium risk of bias |
| Shearer et al. 1996 (40) | 2 | 0 | 1 | 2 | 2 | 0 | 0 | 2 | 2 | 0 | 2 | 2 | 15 | 62,5: medium risk of bias |
| Souza et al. 2012 (41) | 2 | 0 | 1 | 2 | 2 | 0 | 0 | 2 | 1 | 0 | 2 | 2 | 14 | 58,33: medium risk of bias |
| Suárez et al. 2003 (42) | 2 | 0 | 1 | 2 | 2 | 0 | 0 | 2 | 0 | 0 | 1 | 2 | 12 | 50: medium risk of bias |
| Subasi et al. 2012 (43) | 2 | 0 | 1 | 2 | 2 | 0 | 0 | 2 | 1 | 0 | 2 | 1 | 13 | 54,16: medium risk of bias |
| Vigolo et al. 2015 (44) | 2 | 1 | 2 | 2 | 2 | 1 | 2 | 2 | 1 | 2 | 2 | 2 | 21 | 87,5: low risk of bias |
| Vojdani et al. 2015 (45) | 2 | 1 | 1 | 2 | 2 | 0 | 0 | 2 | 0 | 0 | 2 | 2 | 14 | 58,33: medium risk of bias |
| Yadav et al. 2023 (46) | 2 | 0 | 1 | 2 | 1 | 0 | 0 | 2 | 0 | 0 | 2 | 2 | 12 | 50: medium risk of bias |
| Zhao et al. 2003 (47) | 2 | 0 | 1 | 2 | 2 | 1 | 0 | 2 | 0 | 0 | 1 | 2 | 13 | 54,16: medium risk of bias |
| ElGendy et al. 2025 | 1 | 2 | 2 | 2 | 2 | 0 | 0 | 2 | 0 | 0 | 2 | 2 | 15 | 62,5: medium risk of bias |
| Mancuso et al. 2025 | 2 | 2 | 2 | 2 | 2 | 1 | 0 | 2 | 0 | 0 | 2 | 2 | 17 | 70,83: low risk of bias |
| Salama et al. 2025 | 2 | 1 | 2 | 2 | 2 | 2 | 0 | 2 | 1 | 0 | 2 | 2 | 18 | 75: low risk of bias |

∗ Final score = (Total score×100)/ (2×number of criteria applicable)

>70%=low risk of bias, 50% to 70%=medium risk of bias, and <50% high risk of bias (48)

**REFERENCES**

1. Al-Makramani BM, Razak AA, Abu-Hassan MI, Sulaiman E, Loon LJ, Yahya NA. Marginal integrity of turkom-cera compared to other all-ceramic materials: effect of finish line. Int J Prosthodont. 2011;24(4):379-81.

2. Angerame D, De Biasi M, Agostinetto M, Franzò A, Marchesi G. Influence of preparation designs on marginal adaptation and failure load of full-coverage occlusal veneers after thermomechanical aging simulation. J Esthet Restor Dent. 2019;31(3):280-9.

3. Ates SM, Yesil Duymus Z. Influence of Tooth Preparation Design on Fitting Accuracy of CAD-CAM Based Restorations. J Esthet Restor Dent. 2016;28(4):238-46.

4. Ates SM, Yesil Duymus Z, Caglar I, Hologlu B. The effect of veneering on the marginal fit of CAD/CAM-generated, copy-milled, and cast metal copings. Clin Oral Investig. 2017;21(8):2553-60.

5. Baig MR, Tan KB, Nicholls JI. Evaluation of the marginal fit of a zirconia ceramic computer-aided machined (CAM) crown system. J Prosthet Dent. 2010;104(4):216-27.

6. Baig MR, Akbar AA, Embaireeg M. Effect of Finish Line Design on the Fit Accuracy of CAD/CAM Monolithic Polymer-Infiltrated Ceramic-Network Fixed Dental Prostheses: An In Vitro Study. Polymers (Basel). 2021;13(24).

7. Baig MR, Al-Tarakemah Y, Kasim NHA, Omar R. Evaluation of the marginal fit of a CAD/CAM zirconia-based ceramic crown system. Int J Prosthodont. 2022;35(3):319–29.

8. Bindl A, Mörmann WH. Fit of all-ceramic posterior fixed partial denture frameworks in vitro. Int J Periodontics Restorative Dent. 2007;27(6):567-75.

9. Cetik S, Bahrami B, Fossoyeux I, Atash R. Adaptation of zirconia crowns created by conventional versus optical impression: in vitro study. J Adv Prosthodont. 2017;9(3):208-16.

10. Cho SH, Nagy WW, Goodman JT, Solomon E, Koike M. The effect of multiple firings on the marginal integrity of pressable ceramic single crowns. J Prosthet Dent. 2012;107(1):17-23.

11. Comlekoglu M, Dundar M, Ozcan M, Gungor M, Gokce B, Artunc C. Influence of cervical finish line type on the marginal adaptation of zirconia ceramic crowns. Oper Dent. 2009;34(5):586-92.

12. Demir N, Ozturk AN, Malkoc MA. Evaluation of the marginal fit of full ceramic crowns by the microcomputed tomography (micro-CT) technique. Eur J Dent. 2014;8(4):437-44.

13. Elhussieny M, Ismail M, Mohsen CA. Aging Effect on Marginal Gap Distance and Cyclic Loading of Two Different Ceramic Crowns. Indian Journal of Public Health Research & Development. 2020;11(3):1869-73.

14. Elsherbini M, Sakrana AA, Amin RA, Diaa M, Özcan M, Al-Zordk W. A micro-computed tomography analysis of internal and marginal fits of fixed partial dentures: Effect of preparation finish line designs on monolithic zirconia and heat-pressed zirconia-reinforced lithium disilicate. Journal of Prosthodontics. 2023;32(5):e90-e9.

15. Euán R, Figueras-Álvarez O, Cabratosa-Termes J, Brufau-de Barberà M, Gomes-Azevedo S. Comparison of the marginal adaptation of zirconium dioxide crowns in preparations with two different finish lines. J Prosthodont. 2012;21(4):291-5.

16. Euán R, Figueras-Álvarez O, Cabratosa-Termes J, Oliver-Parra R. Marginal adaptation of zirconium dioxide copings: influence of the CAD/CAM system and the finish line design. J Prosthet Dent. 2014;112(2):155-62.

17. Faruqi S, Ganji KK, Bandela V, Nagarajappa AK, Mohamed RN, Ahmed MA, et al. Digital assessment of marginal accuracy in ceramic crowns fabricated with different marginal finish line configurations. J Esthet Restor Dent. 2022;34(5):789-95.

18. Ferrari M, Marucci A, Cagidiaco EF, Pontoriero DI, Fuzzi M. Sealing Ability of New Translucent Zirconia Crowns Made with Digital Workflow and Cemented with Different Types of Cement. Int J Periodontics Restorative Dent. 2021;41(5):703-10.

19. Gavara SG, Jain S, Gupta H, Sharma S, Panwar P, Momin MS. Comparative Effect of No Finish Line, Heavy Chamfer, and Shoulder Marginal Designs on the Fracture Resistance of Zirconia (Cercon) Ceramic Restoration: An In Vitro Study. Cureus. 2023;15(5):e39009.

20. Ahmadzadeh A, Godazpour R, Jafarizadeh A. Comparison of shoulder & chamfer finish line designs on marginal adaptation of IPS e. 2020.

21. Habib SR, Al Ajmi MG, Al Dhafyan M, Jomah A, Abualsaud H, Almashali M. Effect of Margin Designs on the Marginal Adaptation of Zirconia Copings. Acta Stomatol Croat. 2017;51(3):179-87.

22. Ibraheem A, Abdullah L. Evaluation of Post Cementation Marginal Seating of Monolithic Zirconia Crown Restorations Using Different Preparation Designs (A comparative in vitro study). Indian Journal of Forensic Medicine & Toxicology. 2020;14:787.

23. Jalali H, Sadighpour L, Miri A, Shamshiri AR. Comparison of Marginal Fit and Fracture Strength of a CAD/CAM Zirconia Crown with Two Preparation Designs. J Dent (Tehran). 2015;12(12):874-81.

24. Ji MK, Park JH, Park SW, Yun KD, Oh GJ, Lim HP. Evaluation of marginal fit of 2 CAD-CAM anatomic contour zirconia crown systems and lithium disilicate glass-ceramic crown. J Adv Prosthodont. 2015;7(4):271-7.

25. Koç E, Öngül D, Şermet B. A comparative study of marginal fit of copings prepared with various techniques on different implant abutments. Dent Mater J. 2016;35(3):447-53.

26. Komine F, Iwai T, Kobayashi K, Matsumura H. Marginal and internal adaptation of zirconium dioxide ceramic copings and crowns with different finish line designs. Dent Mater J. 2007;26(5):659-64.

27. Krasanaki ME, Pelekanos S, Andreiotelli M, Koutayas SO, Eliades G. X-ray microtomographic evaluation of the influence of two preparation types on marginal fit of CAD/CAM alumina copings: a pilot study. Int J Prosthodont. 2012;25(2):170-2.

28. Kusaba K, Komine F, Honda J, Kubochi K, Matsumura H. Effect of preparation design on marginal and internal adaptation of translucent zirconia laminate veneers. Eur J Oral Sci. 2018;126(6):507-11.

29. Mertsöz B, Ongun S, Ulusoy M. In-Vitro Investigation of Marginal Adaptation and Fracture Resistance of Resin Matrix Ceramic Endo-Crown Restorations. Materials. 2023;16(5):2059.

30. Mitchell CA, Pintado MR, Douglas WH. Nondestructive, in vitro quantification of crown margins. J Prosthet Dent. 2001;85(6):575-84.

31. Miura S, Inagaki R, Kasahara S, Yoda M. Fit of zirconia all-ceramic crowns with different cervical margin designs, before and after porcelain firing and glazing. Dent Mater J. 2014;33(4):484-9.

32. Pera P, Gilodi S, Bassi F, Carossa S. In vitro marginal adaptation of alumina porcelain ceramic crowns. J Prosthet Dent. 1994;72(6):585-90.

33. Quintas AF, Oliveira F, Bottino MA. Vertical marginal discrepancy of ceramic copings with different ceramic materials, finish lines, and luting agents: an in vitro evaluation. J Prosthet Dent. 2004;92(3):250-7.

34. Re D, Cerutti F, Augusti G, Cerutti A, Augusti D. Comparison of marginal fit of Lava CAD/CAM crown-copings with two finish lines. Int J Esthet Dent. 2014;9(3):426-35.

35. Ribeiro IL, Campos F, Sousa RS, Alves ML, Rodrigues DM, Souza RO, et al. Marginal and internal discrepancies of zirconia copings: effects of milling system and finish line design. Indian J Dent Res. 2015;26(1):15-20.

36. Rinke S, Margraf G, Jahn L, Hüls A. [The quality appraisal of copy-milled complete-ceramic crown structures (Celay/In-Ceram)]. Schweiz Monatsschr Zahnmed. 1994;104(12):1495-9.

37. Rizonaki M, Jacquet W, Bottenberg P, Depla L, Boone M, De Coster PJ. Evaluation of marginal and internal fit of lithium disilicate CAD-CAM crowns with different finish lines by using a micro-CT technique. J Prosthet Dent. 2022;127(6):890-8.

38. Porojan L, Topala F, Porojan S. Marginal Design Evaluation for CAM Obtained Zirconia Based Crown Frameworks. Advanced Materials Research. 2011;213:349-53.

39. Sayed O, Elbolok A, Amgad S. ASSESSMENT OF INTERNNAL ADAPTATION OF CAD CAM ALL CERAMIC CROWNS WITH TWO DIFFERENT MARGIN DESIGNS. Egyptian Dental Journal. 2023;69(1):583-92.

40. Shearer B, Gough MB, Setchell DJ. Influence of marginal configuration and porcelain addition on the fit of In-Ceram crowns. Biomaterials. 1996;17(19):1891-5.

41. Souza RO, Özcan M, Pavanelli CA, Buso L, Lombardo GH, Michida SM, et al. Marginal and internal discrepancies related to margin design of ceramic crowns fabricated by a CAD/CAM system. J Prosthodont. 2012;21(2):94-100.

42. Suárez MJ, González de Villaumbrosia P, Pradíes G, Lozano JF. Comparison of the marginal fit of Procera AllCeram crowns with two finish lines. Int J Prosthodont. 2003;16(3):229-32.

43. Subasi G, Ozturk N, Inan O, Bozogullari N. Evaluation of marginal fit of two all-ceramic copings with two finish lines. Eur J Dent. 2012;6(2):163-8.

44. Vigolo P, Mutinelli S, Biscaro L, Stellini E. An In Vivo Evaluation of the Fit of Zirconium-Oxide Based, Ceramic Single Crowns with Vertical and Horizontal Finish Line Preparations. J Prosthodont. 2015;24(8):603-9.

45. Vojdani M, Safari A, Mohaghegh M, Pardis S, Mahdavi F. The effect of porcelain firing and type of finish line on the marginal fit of zirconia copings. J Dent (Shiraz). 2015;16(2):113-20.

46. Yadav P, Sharma V, Paliwal J, Meena KK, Madaan R, Gurjar B. An In Vitro Comparison of Zirconia and Hybrid Ceramic Crowns With Heavy Chamfer and Shoulder Finish Lines. Cureus. 2023;15(1):e33940.

47. Zhao YF, Wang HR, Li Y. [The effect of tooth preparation design on the CAD/CAM all-ceramic coping crown's fitness]. Zhonghua Kou Qiang Yi Xue Za Zhi. 2003;38(5):330-2.

48. Sheth VH, Shah NP, Jain R, Bhanushali N, Bhatnagar V. Development and validation of a risk-of-bias tool for assessing in vitro studies conducted in dentistry: The QUIN. J Prosthet Dent. 2022.
