# Supplementary material for: Impact of finish line designs on the adaptation of ceramic fixed dental prostheses: a systematic review and network meta-analysis
Source: BMC Oral Health. 2025 Jul 3;25:1085. doi: 10.1186/s12903-025-06433-0 (PMC12231902; doi:10.1186/s12903-025-06433-0)
Supplement: Supplementary file 4 — Supplementary Material 4 [file 12903_2025_6433_MOESM4_ESM.docx]

**Results of CINeMA (Confidence in Network Meta-Analysis)**

**Supplementary material 3. Table** **1.: Marginal gap**

| **Comparison** | **Number of studies** | **Within-study bias** | **Reporting bias** | **Indirectness** | **Imprecision** | **Heterogeneity** | **Incoherence** | **Confidence rating** | **Reason(s) for downgrading** |
| --- | --- | --- | --- | --- | --- | --- | --- | --- | --- |
| **Chamfer:Rounded shoulder** | 15 | Some concerns | Low risk | No concerns | Major concerns | No concerns | Major concerns | High | ["Within-study bias","Imprecision","Incoherence"] |
| **Chamfer:Shoulder** | 11 | Some concerns | Low risk | No concerns | Major concerns | No concerns | No concerns | High | ["Within-study bias","Imprecision"] |
| **Chamfer:Vertical** | 4 | Some concerns | Low risk | No concerns | No concerns | Major concerns | Major concerns | High | ["Within-study bias","Heterogeneity","Incoherence"] |
| **Rounded shoulder:Shoulder** | 1 | Some concerns | Low risk | No concerns | Major concerns | No concerns | No concerns | High | ["Within-study bias","Imprecision"] |
| **Rounded shoulder:Vertical** | 4 | Some concerns | Low risk | No concerns | Major concerns | No concerns | No concerns | High | ["Within-study bias","Imprecision"] |
| **Shoulder:Vertical** | 1 | Some concerns | Low risk | No concerns | No concerns | Major concerns | Major concerns | High | ["Within-study bias","Heterogeneity","Incoherence"] |

**Supplementary material 3. Table** **2.: Absolute marginal discrepancy**

| **Comparison** | **Number of studies** | **Within-study bias** | **Reporting bias** | **Indirectness** | **Imprecision** | **Heterogeneity** | **Incoherence** | **Confidence rating** | **Reason(s) for downgrading** |
| --- | --- | --- | --- | --- | --- | --- | --- | --- | --- |
| **Chamfer:Rounded shoulder** | 7 | Some concerns | Low risk | No concerns | No concerns | Major concerns | No concerns | High | ["Heterogeneity"] |
| **Chamfer:Shoulder** | 2 | Some concerns | Low risk | No concerns | Major concerns | No concerns | No concerns | High | ["Imprecision"] |
| **Chamfer:Vertical** | 2 | Some concerns | Low risk | No concerns | Major concerns | No concerns | No concerns | High | ["Imprecision"] |
| **Rounded shoulder:Vertical** | 2 | Some concerns | Low risk | No concerns | Major concerns | No concerns | No concerns | High | ["Imprecision"] |
| **Rounded shoulder:Shoulder** | 0 | Some concerns | Low risk | No concerns | Major concerns | No concerns | No concerns | High | ["Imprecision"] |
| **Shoulder:Vertical** | 0 | Some concerns | Low risk | No concerns | Major concerns | No concerns | No concerns | High | ["Imprecision"] |

**Supplementary material 3. Table** **3.: Internal gap**

| **Comparison** | **Number of studies** | **Within-study bias** | **Reporting bias** | **Indirectness** | **Imprecision** | **Heterogeneity** | **Incoherence** | **Confidence rating** | **Reason(s) for downgrading** |
| --- | --- | --- | --- | --- | --- | --- | --- | --- | --- |
| **Chamfer:Rounded shoulder** | 8 | Some concerns | Low risk | No concerns | No concerns | Major concerns | No concerns | High | ["Within-study bias","Heterogeneity"] |
| **Chamfer:Shoulder** | 3 | Some concerns | Low risk | No concerns | Major concerns | No concerns | No concerns | High | ["Within-study bias","Imprecision"] |
| **Chamfer:Vertical** | 3 | Some concerns | Low risk | No concerns | Major concerns | No concerns | No concerns | High | ["Within-study bias","Imprecision"] |
| **Rounded shoulder:Shoulder** | 1 | Some concerns | Low risk | No concerns | Major concerns | No concerns | No concerns | High | ["Within-study bias","Imprecision"] |
| **Rounded shoulder:Vertical** | 1 | Some concerns | Low risk | No concerns | Major concerns | No concerns | No concerns | High | ["Within-study bias","Imprecision"] |
| **Shoulder:Vertical** | 1 | Some concerns | Low risk | No concerns | Major concerns | No concerns | No concerns | High | ["Within-study bias","Imprecision"] |

**Supplementary material 3. Table** **4.: Cemented marginal gap**

| **Comparison** | **Number of studies** | **Within-study bias** | **Reporting bias** | **Indirectness** | **Imprecision** | **Heterogeneity** | **Incoherence** | **Confidence rating** | **Reason(s) for downgrading** |
| --- | --- | --- | --- | --- | --- | --- | --- | --- | --- |
| **Chamfer:Rounded shoulder** | 9 | Some concerns | Low risk | No concerns | Major concerns | No concerns | Major concerns | High | ["Imprecision","Incoherence"] |
| **Chamfer:Shoulder** | 4 | Some concerns | Low risk | No concerns | Major concerns | No concerns | Major concerns | High | ["Imprecision","Incoherence"] |
| **Chamfer:Vertical** | 3 | Some concerns | Low risk | No concerns | Major concerns | No concerns | No concerns | High | ["Imprecision"] |
| **Rounded shoulder:Vertical** | 4 | Some concerns | Low risk | No concerns | Major concerns | No concerns | No concerns | High | ["Imprecision"] |
| **Rounded shoulder:Shoulder** | 0 | Some concerns | Low risk | No concerns | Major concerns | No concerns | Major concerns | High | ["Imprecision","Incoherence"] |
| **Shoulder:Vertical** | 0 | Some concerns | Low risk | No concerns | Major concerns | No concerns | Major concerns | High | ["Imprecision","Incoherence"] |

**Supplementary material 3. Table** **5.: Not cemented marginal gap**

| **Comparison** | **Number of studies** | **Within-study bias** | **Reporting bias** | **Indirectness** | **Imprecision** | **Heterogeneity** | **Incoherence** | **Confidence rating** | **Reason(s) for downgrading** |
| --- | --- | --- | --- | --- | --- | --- | --- | --- | --- |
| **Chamfer:Rounded shoulder** | 6 | No concerns | Low risk | No concerns | Major concerns | No concerns | Major concerns | High | ["Imprecision","Incoherence"] |
| **Chamfer:Shoulder** | 9 | Some concerns | Low risk | No concerns | Major concerns | No concerns | Some concerns | High | ["Within-study bias","Imprecision","Incoherence"] |
| **Chamfer:Vertical** | 1 | Some concerns | Low risk | No concerns | No concerns | No concerns | Major concerns | High | ["Within-study bias","Incoherence"] |
| **Rounded shoulder:Shoulder** | 1 | Some concerns | Low risk | No concerns | Major concerns | No concerns | No concerns | High | ["Within-study bias","Imprecision"] |
| **Rounded shoulder:Vertical** | 1 | No concerns | Low risk | No concerns | No concerns | No concerns | Major concerns | High | ["Incoherence"] |
| **Shoulder:Vertical** | 1 | Some concerns | Low risk | No concerns | No concerns | No concerns | Major concerns | High | ["Within-study bias","Incoherence"] |

**Supplementary material 3. Table** **6.: Cemented absolute marginal discrepancy**

| **Comparison** | **Number of studies** | **Within-study bias** | **Reporting bias** | **Indirectness** | **Imprecision** | **Heterogeneity** | **Incoherence** | **Confidence rating** | **Reason(s) for downgrading** |
| --- | --- | --- | --- | --- | --- | --- | --- | --- | --- |
| **Chamfer:Rounded shoulder** | 5 | Some concerns | Low risk | No concerns | No concerns | Major concerns | No concerns | High | ["Within-study bias","Heterogeneity"] |
| **Chamfer:Shoulder** | 2 | Some concerns | Low risk | No concerns | Major concerns | No concerns | No concerns | High | ["Within-study bias","Imprecision"] |
| **Chamfer:Vertical** | 2 | Some concerns | Low risk | No concerns | Major concerns | No concerns | No concerns | High | ["Within-study bias","Imprecision"] |
| **Rounded shoulder:Vertical** | 2 | Some concerns | Low risk | No concerns | Major concerns | No concerns | No concerns | High | ["Within-study bias","Imprecision"] |
| **Rounded shoulder:Shoulder** | 0 | Some concerns | Low risk | No concerns | Major concerns | No concerns | No concerns | High | ["Within-study bias","Imprecision"] |
| **Shoulder:Vertical** | 0 | Some concerns | Low risk | No concerns | Major concerns | No concerns | No concerns | High | ["Within-study bias","Imprecision"] |

**Supplementary material 3. Table** **7.: Cemented internal gap**

| **Comparison** | **Number of studies** | **Within-study bias** | **Reporting bias** | **Indirectness** | **Imprecision** | **Heterogeneity** | **Incoherence** | **Confidence rating** | **Reason(s) for downgrading** |
| --- | --- | --- | --- | --- | --- | --- | --- | --- | --- |
| **Chamfer:Rounded shoulder** | 6 | Some concerns | Low risk | No concerns | No concerns | Major concerns | No concerns | High | ["Within-study bias","Heterogeneity"] |
| **Chamfer:Shoulder** | 1 | Some concerns | Low risk | No concerns | Major concerns | No concerns | No concerns | High | ["Within-study bias","Imprecision"] |
| **Chamfer:Vertical** | 2 | Some concerns | Low risk | No concerns | Major concerns | No concerns | No concerns | High | ["Within-study bias","Imprecision"] |
| **Rounded shoulder:Vertical** | 1 | Some concerns | Low risk | No concerns | Major concerns | No concerns | No concerns | High | ["Within-study bias","Imprecision"] |
| **Rounded shoulder:Shoulder** | 0 | Some concerns | Low risk | No concerns | Major concerns | No concerns | No concerns | High | ["Within-study bias","Imprecision"] |
| **Shoulder:Vertical** | 0 | Some concerns | Low risk | No concerns | Major concerns | No concerns | No concerns | High | ["Within-study bias","Imprecision"] |

**Supplementary material 3. Table** **8. Not cemented internal gap**

| **Comparison** | **Number of studies** | **Within-study bias** | **Reporting bias** | **Indirectness** | **Imprecision** | **Heterogeneity** | **Incoherence** | **Confidence rating** | **Reason(s) for downgrading** |
| --- | --- | --- | --- | --- | --- | --- | --- | --- | --- |
| **Chamfer:Rounded shoulder** | 2 | Some concerns | Low risk | No concerns | Major concerns | No concerns | No concerns | High | ["Within-study bias","Imprecision"] |
| **Chamfer:Shoulder** | 2 | Some concerns | Low risk | No concerns | No concerns | Major concerns | No concerns | High | ["Within-study bias","Heterogeneity"] |
| **Chamfer:Vertical** | 1 | Some concerns | Low risk | No concerns | Major concerns | No concerns | No concerns | High | ["Within-study bias","Imprecision"] |
| **Rounded shoulder:Shoulder** | 1 | Some concerns | Low risk | No concerns | Major concerns | No concerns | No concerns | High | ["Within-study bias","Imprecision"] |
| **Shoulder:Vertical** | 1 | Some concerns | Low risk | No concerns | Major concerns | No concerns | No concerns | High | ["Within-study bias","Imprecision"] |
| **Rounded shoulder:Vertical** | 0 | Some concerns | Low risk | No concerns | Major concerns | No concerns | Major concerns | High | ["Within-study bias","Imprecision",  "Incoherence"] |

**Supplementary material 3. Table** **9.: CAD/CAM marginal gap**

| **Comparison** | **Number of studies** | **Within-study bias** | **Reporting bias** | **Indirectness** | **Imprecision** | **Heterogeneity** | **Incoherence** | **Confidence rating** | **Reason(s) for downgrading** |
| --- | --- | --- | --- | --- | --- | --- | --- | --- | --- |
| **Chamfer:Rounded shoulder** | 12 | Some concerns | Low risk | No concerns | Major concerns | No concerns | Major concerns | High | ["Within-study bias","Imprecision","Incoherence"] |
| **Chamfer:Shoulder** | 9 | Some concerns | Low risk | No concerns | Major concerns | No concerns | No concerns | High | ["Within-study bias","Imprecision"] |
| **Chamfer:Vertical** | 3 | Some concerns | Low risk | No concerns | Major concerns | No concerns | Major concerns | High | ["Within-study bias","Imprecision","Incoherence"] |
| **Rounded shoulder:Shoulder** | 1 | Some concerns | Low risk | No concerns | Major concerns | No concerns | No concerns | High | ["Within-study bias","Imprecision"] |
| **Rounded shoulder:Vertical** | 3 | Some concerns | Low risk | No concerns | Major concerns | No concerns | No concerns | High | ["Within-study bias","Imprecision"] |
| **Shoulder:Vertical** | 1 | Some concerns | Low risk | No concerns | Major concerns | No concerns | No concerns | High | ["Within-study bias","Imprecision"] |

**Supplementary material 3. Table** **10.: Conventional marginal gap**

| **Comparison** | **Number of studies** | **Within-study bias** | **Reporting bias** | **Indirectness** | **Imprecision** | **Heterogeneity** | **Incoherence** | **Confidence rating** | **Reason(s) for downgrading** |
| --- | --- | --- | --- | --- | --- | --- | --- | --- | --- |
| **Chamfer:Rounded shoulder** | 5 | Some concerns | Low risk | No concerns | Major concerns | No concerns | No concerns | High | ["Within-study bias","Imprecision"] |
| **Chamfer:Shoulder** | 4 | Some concerns | Low risk | No concerns | Major concerns | No concerns | No concerns | High | ["Within-study bias","Imprecision"] |
| **Chamfer:Vertical** | 1 | Some concerns | Low risk | No concerns | No concerns | Major concerns | No concerns | High | ["Within-study bias","Heterogeneity"] |
| **Rounded shoulder:Vertical** | 1 | Some concerns | Low risk | No concerns | No concerns | Major concerns | No concerns | High | ["Within-study bias","Heterogeneity"] |
| **Rounded shoulder:Shoulder** | 0 | Some concerns | Low risk | No concerns | Major concerns | No concerns | No concerns | High | ["Within-study bias","Imprecision"] |
| **Shoulder:Vertical** | 0 | Some concerns | Low risk | No concerns | No concerns | No concerns | No concerns | High | ["Within-study bias"] |

**Supplementary material 3. Table** **11.: CAD/CAM absolute marginal discrepancy**

| **Comparison** | **Number of studies** | **Within-study bias** | **Reporting bias** | **Indirectness** | **Imprecision** | **Heterogeneity** | **Incoherence** | **Confidence rating** | **Reason(s) for downgrading** |
| --- | --- | --- | --- | --- | --- | --- | --- | --- | --- |
| **Chamfer:Rounded shoulder** | 5 | Some concerns | Low risk | No concerns | No concerns | Major concerns | No concerns | High | ["Within-study bias","Heterogeneity"] |
| **Chamfer:Shoulder** | 2 | Some concerns | Low risk | No concerns | Major concerns | No concerns | No concerns | High | ["Within-study bias","Imprecision"] |
| **Chamfer:Vertical** | 1 | Some concerns | Low risk | No concerns | No concerns | Major concerns | No concerns | High | ["Within-study bias","Heterogeneity"] |
| **Rounded shoulder:Vertical** | 1 | Some concerns | Low risk | No concerns | No concerns | No concerns | No concerns | High | ["Within-study bias"] |
| **Rounded shoulder:Shoulder** | 0 | Some concerns | Low risk | No concerns | Major concerns | No concerns | No concerns | High | ["Within-study bias","Imprecision"] |
| **Shoulder:Vertical** | 0 | Some concerns | Low risk | No concerns | Major concerns | No concerns | No concerns | High | ["Within-study bias","Imprecision"] |

**Supplementary material 3. Table** **12.: Conventional absolute marginal discrepancy**

| **Comparison** | **Number of studies** | **Within-study bias** | **Reporting bias** | **Indirectness** | **Imprecision** | **Heterogeneity** | **Incoherence** | **Confidence rating** | **Reason(s) for downgrading** |
| --- | --- | --- | --- | --- | --- | --- | --- | --- | --- |
| **Chamfer:Rounded shoulder** | 2 | Some concerns | Low risk | No concerns | No concerns | Major concerns | No concerns | High | ["Within-study bias", "Heterogeneity"] |
| **Chamfer:Shoulder** | 1 | Some concerns | Low risk | No concerns | No concerns | No concerns | No concerns | High | ["Within-study bias"] |
| **Chamfer:Vertical** | 1 | Some concerns | Low risk | No concerns | No concerns | No concerns | No concerns | High | ["Within-study bias"] |
| **Rounded shoulder:Vertical** | 1 | Some concerns | Low risk | No concerns | Some concerns | No concerns | No concerns | High | ["Within-study bias", "Imprecision"] |
| **Rounded shoulder:Shoulder** | 0 | Some concerns | Low risk | No concerns | Major concerns | No concerns | No concerns | High | ["Within-study bias","Imprecision"] |
| **Shoulder:Vertical** | 0 | Some concerns | Low risk | No concerns | No concerns | No concerns | No concerns | High | ["Within-study bias"] |

**Supplementary material 3. Table** **13.: CAD/CAM internal gap**

| **Comparison** | **Number of studies** | **Within-study bias** | **Reporting bias** | **Indirectness** | **Imprecision** | **Heterogeneity** | **Incoherence** | **Confidence rating** | **Reason(s) for downgrading** |
| --- | --- | --- | --- | --- | --- | --- | --- | --- | --- |
| **Chamfer:Rounded shoulder** | 8 | Some concerns | Low risk | No concerns | No concerns | Major concerns | No concerns | High | ["Within-study bias","Heterogeneity"] |
| **Chamfer:Shoulder** | 2 | Some concerns | Low risk | No concerns | No concerns | Major concerns | No concerns | High | ["Within-study bias","Heterogeneity"] |
| **Chamfer:Vertical** | 3 | Some concerns | Low risk | No concerns | Major concerns | No concerns | No concerns | High | ["Within-study bias","Imprecision"] |
| **Rounded shoulder:Shoulder** | 1 | Some concerns | Low risk | No concerns | Major concerns | No concerns | No concerns | High | ["Within-study bias","Imprecision"] |
| **Rounded shoulder:Vertical** | 1 | Some concerns | Low risk | No concerns | No concerns | Major concerns | No concerns | High | ["Within-study bias","Heterogeneity"] |
| **Shoulder:Vertical** | 1 | Some concerns | Low risk | No concerns | Major concerns | No concerns | No concerns | High | ["Within-study bias","Imprecision"] |

**Supplementary material 3. Table** **14.: Marginal gap evaluation using the direct view technique:**

| **Comparison** | **Number of studies** | **Within-study bias** | **Reporting bias** | **Indirectness** | **Imprecision** | **Heterogeneity** | **Incoherence** | **Confidence rating** | **Reason(s) for downgrading** |
| --- | --- | --- | --- | --- | --- | --- | --- | --- | --- |
| **Chamfer:Rounded shoulder** | 9 | Some concerns | Low risk | No concerns | Major concerns | No concerns | No concerns | High | ["Within-study bias","Imprecision"] |
| **Chamfer:Shoulder** | 7 | Some concerns | Low risk | No concerns | Major concerns | No concerns | No concerns | High | ["Within-study bias","Imprecision"] |
| **Chamfer:Vertical** | 1 | Some concerns | Low risk | No concerns | No concerns | Major concerns | Major concerns | High | ["Within-study bias","Heterogeneity","Incoherence"] |
| **Rounded shoulder:Shoulder** | 1 | Some concerns | Low risk | No concerns | Major concerns | No concerns | No concerns | High | ["Within-study bias","Imprecision"] |
| **Rounded shoulder:Vertical** | 2 | Some concerns | Low risk | No concerns | No concerns | Major concerns | No concerns | High | ["Within-study bias","Heterogeneity"] |
| **Shoulder:Vertical** | 0 | Some concerns | Low risk | No concerns | No concerns | Major concerns | Major concerns | High | ["Within-study bias","Heterogeneity","Incoherence"] |

**Supplementary material 3. Table** **15.: Marginal gap evaluation using the cross sectioned technique:**

| **Comparison** | **Number of studies** | **Within-study bias** | **Reporting bias** | **Indirectness** | **Imprecision** | **Heterogeneity** | **Incoherence** | **Confidence rating** | **Reason(s) for downgrading** |
| --- | --- | --- | --- | --- | --- | --- | --- | --- | --- |
| **Chamfer:Rounded shoulder** | 4 | Some concerns | Low risk | No concerns | Major concerns | No concerns | No concerns | High | ["Within-study bias","Imprecision"] |
| **Chamfer:Shoulder** | 2 | Some concerns | Low risk | No concerns | Major concerns | No concerns | No concerns | High | ["Within-study bias","Imprecision"] |
| **Chamfer:Vertical** | 2 | Some concerns | Low risk | No concerns | Major concerns | No concerns | No concerns | High | ["Within-study bias","Imprecision"] |
| **Rounded shoulder:Vertical** | 1 | Some concerns | Low risk | No concerns | Major concerns | No concerns | No concerns | High | ["Within-study bias","Imprecision"] |
| **Shoulder:Vertical** | 1 | Some concerns | Low risk | No concerns | Major concerns | No concerns | No concerns | High | ["Within-study bias","Imprecision"] |
| **Rounded shoulder:Shoulder** | 0 | Some concerns | Low risk | No concerns | Major concerns | No concerns | No concerns | High | ["Within-study bias","Imprecision"] |

**Supplementary material 3. Table** **15.: Marginal gap evaluation using the micro-CT technique:**

| **Comparison** | **Number of studies** | **Within-study bias** | **Reporting bias** | **Indirectness** | **Imprecision** | **Heterogeneity** | **Incoherence** | **Confidence rating** | **Reason(s) for downgrading** |
| --- | --- | --- | --- | --- | --- | --- | --- | --- | --- |
| **Chamfer:Rounded shoulder** | 2 | Some concerns | Low risk | No concerns | Major concerns | No concerns | No concerns | High | ["Within-study bias" Imprecision"," ] |
| **Chamfer:Vertical** | 1 | Some concerns | Low risk | No concerns | Major concerns | No concerns | No concerns | High | ["Within-study bias" Imprecision"," ] |
| **Rounded shoulder:Vertical** | 1 | Some concerns | Low risk | No concerns | Major concerns | No concerns | No concerns | High | ["Within-study bias" Imprecision"," ] |

**Supplementary material 3. Table** **16.: Absolute marginal discrepancy evaluation using the direct view technique:**

| **Comparison** | **Number of studies** | **Within-study bias** | **Reporting bias** | **Indirectness** | **Imprecision** | **Heterogeneity** | **Incoherence** | **Confidence rating** | **Reason(s) for downgrading** |
| --- | --- | --- | --- | --- | --- | --- | --- | --- | --- |
| **Chamfer:**  **Rounded shoulder** | 3 | Some concerns | Low risk | No concerns | Major concerns | No concerns | Major concerns | High | ["Within-study bias","Imprecision","Incoherence"] |
| **Chamfer:**  **Shoulder** | 1 | Some concerns | Low risk | No concerns | Major concerns | No concerns | Major concerns | High | ["Within-study bias","Imprecision","Incoherence"] |
| **Rounded shoulder:Shoulder** | 0 | Some concerns | Low risk | No concerns | Major concerns | No concerns | Major concerns | High | ["Within-study bias","Imprecision","Incoherence"] |

**Supplementary material 3. Table** **17.: Absolute marginal discrepancy evaluation using the cross sectioned technique:**

| **Comparison** | **Number of studies** | **Within-study bias** | **Reporting bias** | **Indirectness** | **Imprecision** | **Heterogeneity** | **Incoherence** | **Confidence rating** | **Reason(s) for downgrading** |
| --- | --- | --- | --- | --- | --- | --- | --- | --- | --- |
| **Chamfer:Rounded shoulder** | 2 | Some concerns | Low risk | No concerns | Major concerns | No concerns | High | High | ["Within-study bias" ","Imprecision","Incoherence"] |
| **Chamfer:Shoulder** | 1 | No concerns | Low risk | No concerns | Major concerns | No concerns | High | High | ["Imprecision","Incoherence"] |
| **Chamfer:Vertical** | 1 | Some concerns | Low risk | No concerns | Major concerns | No concerns | High | High | ["Within-study bias","Imprecision","Incoherence"] |
| **Rounded shoulder:Vertical** | 1 | Some concerns | Low risk | No concerns | Major concerns | No concerns | High | High | ["Within-study bias", "Imprecision","Incoherence"] |
| **Rounded shoulder:Shoulder** | 0 | Some concerns | Low risk | No concerns | Major concerns | No concerns | High | High | ["Within-study bias","Imprecision","Incoherence"] |
| **Shoulder:Vertical** | 0 | Some concerns | Low risk | No concerns | Major concerns | No concerns | High | High | ["Within-study bias""Imprecision","Incoherence"] |

**Supplementary material 3. Table** **18.: Internal gap evaluation using the cross sectioned technique:**

| **Comparison** | **Number of studies** | **Within-study bias** | **Reporting bias** | **Indirectness** | **Imprecision** | **Heterogeneity** | **Incoherence** | **Confidence rating** | **Reason(s) for downgrading** |
| --- | --- | --- | --- | --- | --- | --- | --- | --- | --- |
| **Chamfer:Rounded shoulder** | 4 | Some concerns | Low risk | No concerns | No concerns | Major concerns | No concerns | High | ["Within-study bias","Heterogeneity"] |
| **Chamfer:Shoulder** | 2 | Some concerns | Low risk | No concerns | Major concerns | No concerns | No concerns | High | ["Within-study bias","Imprecision"] |
| **Chamfer:Vertical** | 2 | Some concerns | Low risk | No concerns | Major concerns | No concerns | No concerns | High | ["Within-study bias","Imprecision"] |
| **Shoulder:Vertical** | 1 | Some concerns | Low risk | No concerns | Major concerns | No concerns | No concerns | High | ["Within-study bias","Imprecision"] |
| **Rounded shoulder:Shoulder** | 0 | Some concerns | Low risk | No concerns | Major concerns | No concerns | No concerns | High | ["Within-study bias","Imprecision"] |
| **Rounded shoulder:Vertical** | 0 | Some concerns | Low risk | No concerns | Major concerns | No concerns | No concerns | High | ["Within-study bias","Imprecision"] |

**Supplementary material 3. Table** **19.: Internal gap evaluation using the silicon replica technique:**

| **Comparison** | **Number of studies** | **Within-study bias** | **Reporting bias** | **Indirectness** | **Imprecision** | **Heterogeneity** | **Incoherence** | **Confidence rating** | **Reason(s) for downgrading** |
| --- | --- | --- | --- | --- | --- | --- | --- | --- | --- |
| **Chamfer:Rounded shoulder** | 2 | Some concerns | Low risk | No concerns | Major concerns | No concerns | No concerns | High | ["Within-study bias","Imprecision"] |
| **Chamfer:Shoulder** | 1 | Some concerns | Low risk | No concerns | Major concerns | No concerns | No concerns | High | ["Within-study bias","Imprecision"] |
| **Rounded shoulder:Shoulder** | 1 | Some concerns | Low risk | No concerns | Major concerns | No concerns | No concerns | High | ["Within-study bias","Imprecision"] |

**Supplementary material 3. Table** **20.: Internal gap evaluation using the micro-CT technique:**

| **Comparison** | **Number of studies** | **Within-study bias** | **Reporting bias** | **Indirectness** | **Imprecision** | **Heterogeneity** | **Incoherence** | **Confidence rating** | **Reason(s) for downgrading** |
| --- | --- | --- | --- | --- | --- | --- | --- | --- | --- |
| **Chamfer:Rounded shoulder** | 2 | Some concerns | Low risk | No concerns | Major concerns | No concerns | No concerns | High | ["Within-study bias","Imprecision"] |
| **Chamfer:Vertical** | 1 | Some concerns | Low risk | No concerns | Major concerns | No concerns | No concerns | High | ["Within-study bias","Imprecision"] |
| **Rounded shoulder:Vertical** | 1 | Some concerns | Low risk | No concerns | Major concerns | No concerns | No concerns | High | ["Within-study bias","Imprecision"] |

**Supplementary material 3. Table** **21.: Marginal gap of crowns, endocrowns:**

| **Comparison** | **Number of studies** | **Within-study bias** | **Reporting bias** | **Indirectness** | **Imprecision** | **Heterogeneity** | **Incoherence** | **Confidence rating** | **Reason(s) for downgrading** |
| --- | --- | --- | --- | --- | --- | --- | --- | --- | --- |
| **Chamfer:**  **Rounded shoulder** | 7 | Some concerns | Low risk | No concerns | Major concerns | No concerns | Major concerns | High | ["Imprecision","Incoherence"] |
| **Chamfer:**  **Shoulder** | 8 | Some concerns | Low risk | No concerns | Major concerns | No concerns | No concerns | High | ["Imprecision"] |
| **Chamfer:Vertical** | 3 | Some concerns | Low risk | No concerns | Major concerns | No concerns | Major concerns | High | ["Imprecision","Incoherence"] |
| **Rounded shoulder:**  **Shoulder** | 1 | Some concerns | Low risk | No concerns | Major concerns | No concerns | No concerns | High | ["Imprecision"] |
| **Rounded shoulder:Vertical** | 3 | Some concerns | Low risk | No concerns | Major concerns | No concerns | No concerns | High | ["Imprecision"] |
| **Shoulder:**  **Vertical** | 1 | Some concerns | Low risk | No concerns | No concerns | Major concerns | Major concerns | High | ["Heterogeneity","Incoherence"] |

**Supplementary material 3. Table** **22.: Marginal gap of copings:**

| **Comparison** | **Number of studies** | **Within-study bias** | **Reporting bias** | **Indirectness** | **Imprecision** | **Heterogeneity** | **Incoherence** | **Confidence rating** | **Reason(s) for downgrading** |
| --- | --- | --- | --- | --- | --- | --- | --- | --- | --- |
| **Chamfer:Rounded shoulder** | 6 | Some concerns | Low risk | No concerns | Major concerns | No concerns | Major concerns | High | ["Imprecision","Incoherence"] |
| **Chamfer:Shoulder** | 4 | Some concerns | Low risk | No concerns | Major concerns | No concerns | Major concerns | High | ["Imprecision","Incoherence"] |
| **Rounded shoulder:Shoulder** | 0 | Some concerns | Low risk | No concerns | Major concerns | No concerns | Major concerns | High | ["Imprecision","Incoherence"] |

**Supplementary material 3. Table** **22.: Marginal gap of veneers:**

| **Comparison** | **Number of studies** | **Within-study bias** | **Reporting bias** | **Indirectness** | **Imprecision** | **Heterogeneity** | **Incoherence** | **Confidence rating** | **Reason(s) for downgrading** |
| --- | --- | --- | --- | --- | --- | --- | --- | --- | --- |
| **Chamfer:Rounded shoulder** | 2 | Some concerns | Low risk | No concerns | Major concerns | No concerns | No concerns | High | ["Within-study bias","Imprecision"] |
| **Chamfer:Vertical** | 1 | Some concerns | Low risk | No concerns | Major concerns | No concerns | No concerns | High | ["Within-study bias","Imprecision"] |
| **Rounded shoulder:Vertical** | 1 | Some concerns | Low risk | No concerns | Major concerns | No concerns | No concerns | High | ["Within-study bias","Imprecision"] |

**Supplementary material 3. Table** **22.: Absolute marginal discrepancy of crowns, endorowns:**

| **Comparison** | **Number of studies** | **Within-study bias** | **Reporting bias** | **Indirectness** | **Imprecision** | **Heterogeneity** | **Incoherence** | **Confidence rating** | **Reason(s) for downgrading** |
| --- | --- | --- | --- | --- | --- | --- | --- | --- | --- |
| **Chamfer:Rounded shoulder** | 5 | Some concerns | Low risk | No concerns | Major concerns | No concerns | No concerns | High | ["Within-study bias","Imprecision"] |
| **Chamfer:Shoulder** | 1 | Some concerns | Low risk | No concerns | Major concerns | No concerns | No concerns | High | ["Within-study bias","Imprecision"] |
| **Chamfer:Vertical** | 2 | Some concerns | Low risk | No concerns | Major concerns | No concerns | No concerns | High | ["Within-study bias","Imprecision"] |
| **Rounded shoulder:Vertical** | 2 | Some concerns | Low risk | No concerns | Major concerns | No concerns | No concerns | High | ["Within-study bias","Imprecision"] |
| **Rounded shoulder:Shoulder** | 0 | Some concerns | Low risk | No concerns | Major concerns | No concerns | No concerns | High | ["Within-study bias","Imprecision"] |
| **Shoulder:Vertical** | 0 | Some concerns | Low risk | No concerns | Major concerns | No concerns | No concerns | High | ["Within-study bias","Imprecision"] |

**Supplementary material 3. Table** **23.: Absolute marginal discrepancy of copings:**

| **Comparison** | **Number of studies** | **Within-study bias** | **Reporting bias** | **Indirectness** | **Imprecision** | **Heterogeneity** | **Incoherence** | **Confidence rating** | **Reason(s) for downgrading** |
| --- | --- | --- | --- | --- | --- | --- | --- | --- | --- |
| **Chamfer:Rounded shoulder** | 2 | Some concerns | Low risk | No concerns | Major concerns | No concerns | No concerns | High | ["Within-study bias","Imprecision"] |
| **Chamfer:Shoulder** | 1 | No concerns | Low risk | No concerns | Major concerns | No concerns | No concerns | High | ["Imprecision"] |
| **Rounded shoulder:Shoulder** | 0 | Some concerns | Low risk | No concerns | Major concerns | No concerns | No concerns | High | ["Within-study bias","Imprecision"] |

**Supplementary material 3. Table** **23.: Internal gap of crowns, endocrowns:**

| **Comparison** | **Number of studies** | **Within-study bias** | **Reporting bias** | **Indirectness** | **Imprecision** | **Heterogeneity** | **Incoherence** | **Confidence rating** | **Reason(s) for downgrading** |
| --- | --- | --- | --- | --- | --- | --- | --- | --- | --- |
| **Chamfer:**  **Rounded shoulder** | 3 | Some concerns | Low risk | No concerns | Some concerns | Some concerns | No concerns | High | ["Within-study bias","Imprecision","Heterogeneity"] |
| **Chamfer:**  **Shoulder** | 2 | Some concerns | Low risk | No concerns | No concerns | Major concerns | No concerns | High | ["Within-study bias","Heterogeneity"] |
| **Chamfer:**  **Vertical** | 3 | Some concerns | Low risk | No concerns | Major concerns | No concerns | No concerns | High | ["Within-study bias","Imprecision"] |
| **Rounded shoulder:**  **Shoulder** | 1 | Some concerns | Low risk | No concerns | Major concerns | No concerns | No concerns | High | ["Within-study bias","Imprecision"] |
| **Rounded shoulder:**  **Vertical** | 1 | Some concerns | Low risk | No concerns | Major concerns | No concerns | Major concerns | High | ["Within-study bias","Imprecision","Incoherence"] |
| **Shoulder:**  **Vertical** | 1 | Some concerns | Low risk | No concerns | Major concerns | No concerns | No concerns | High | ["Within-study bias","Imprecision"] |

**Supplementary material 3. Table** **24.: Internal gap of copings:**

| **Comparison** | **Number of studies** | **Within-study bias** | **Reporting bias** | **Indirectness** | **Imprecision** | **Heterogeneity** | **Incoherence** | **Confidence rating** | **Reason(s) for downgrading** |
| --- | --- | --- | --- | --- | --- | --- | --- | --- | --- |
| **Chamfer:Rounded shoulder** | 2 | Some concerns | Low risk | No concerns | Major concerns | No concerns | Major concerns | High | ["Within-study bias","Imprecision","Incoherence"] |
| **Rounded shoulder:Shoulder** | 1 | Major concerns | Low risk | No concerns | Major concerns | No concerns | Major concerns | High | ["Within-study bias","Imprecision","Incoherence"] |
| **Chamfer:Shoulder** | 0 | Major concerns | Low risk | No concerns | Major concerns | No concerns | Major concerns | High | ["Within-study bias", "Imprecision","Incoherence"] |

**Certainty of Evidence** - GRADE rating system

**Supplementary material 3. Table** **25.: Not cemented absolute marginal discrepancy**

| **Certainty assessment** | | | | | | | **№ of patients** | | **Effect** | | **Certainty** | **Importance** |
| --- | --- | --- | --- | --- | --- | --- | --- | --- | --- | --- | --- | --- |
| **№ of studies** | **Study design** | **Risk of bias** | **Inconsistency** | **Indirectness** | **Imprecision** | **Other considerations** | **chamfer** | **rounded shoulder** | **Relative (95% CI)** | **Absolute (95% CI)** |  |  |
| **Absolute marginal discrepancy (assessed with: micrometer):** Chamfer compared to rounded shoulder for ceramic restorations | | | | | | | | | | | | |
| 3 | non-randomised studies | not serious | not serious | not serious | not serious | none | 70 | 70 | - | MD -**9.85 micrometer lower** (-23.65 lower to 4.69 higher) | ⨁⨁⨁⨁ High | CRITICAL |

**CI:** confidence interval; **MD:** mean difference

**Supplementary material 3. Table** **26.:Marginal gap evaluation using the silicon replica technique:**

| **Certainty assessment** | | | | | | | **№ of patients** | | **Effect** | | **Certainty** | **Importance** |
| --- | --- | --- | --- | --- | --- | --- | --- | --- | --- | --- | --- | --- |
| **№ of studies** | **Study design** | **Risk of bias** | **Inconsistency** | **Indirectness** | **Imprecision** | **Other considerations** | **chamfer** | **rounded shoulder** | **Relative (95% CI)** | **Absolute (95% CI)** |  |  |
| **Absolute marginal discrepancy (assessed with: micrometer):** Chamfer compared to rounded shoulder for ceramic restorations | | | | | | | | | | | | |
| 3 | non-randomised studies | not serious | not serious | not serious | not serious | none | 60 | 60 | - | MD -**0.66**  **micrometer lower** (-5.28  lower to 6.54  higher) | ⨁⨁◯◯ Low | CRITICAL |

**Supplementary material 3. Table** **27.:Internal gap of veneers:**

| **Certainty assessment** | | | | | | | **№ of patients** | | **Effect** | | **Certainty** | **Importance** |
| --- | --- | --- | --- | --- | --- | --- | --- | --- | --- | --- | --- | --- |
| **№ of studies** | **Study design** | **Risk of bias** | **Inconsistency** | **Indirectness** | **Imprecision** | **Other considerations** | **chamfer** | **rounded shoulder** | **Relative (95% CI)** | **Absolute (95% CI)** |  |  |
| **Absolute marginal discrepancy (assessed with: micrometer):** Chamfer compared to rounded shoulder for ceramic veneers | | | | | | | | | | | | |
| 2 | non-randomised studies | not serious | not serious | not serious | not serious | none | 28 | 28 | - | MD -**13.47**  **micrometer lower** (-19.21  lower to 46.17  higher) | ⨁⨁◯◯ Low | CRITICAL |
