# Supplementary material for: Impact of finish line designs on the adaptation of ceramic fixed dental prostheses: a systematic review and network meta-analysis
Source: BMC Oral Health. 2025 Jul 3;25:1085. doi: 10.1186/s12903-025-06433-0 (PMC12231902; doi:10.1186/s12903-025-06433-0)
Supplement: Supplementary file 6 — Supplementary Material 6 [file 12903_2025_6433_MOESM6_ESM.docx]

**Consistency-inconsistency plots for each outcomes**


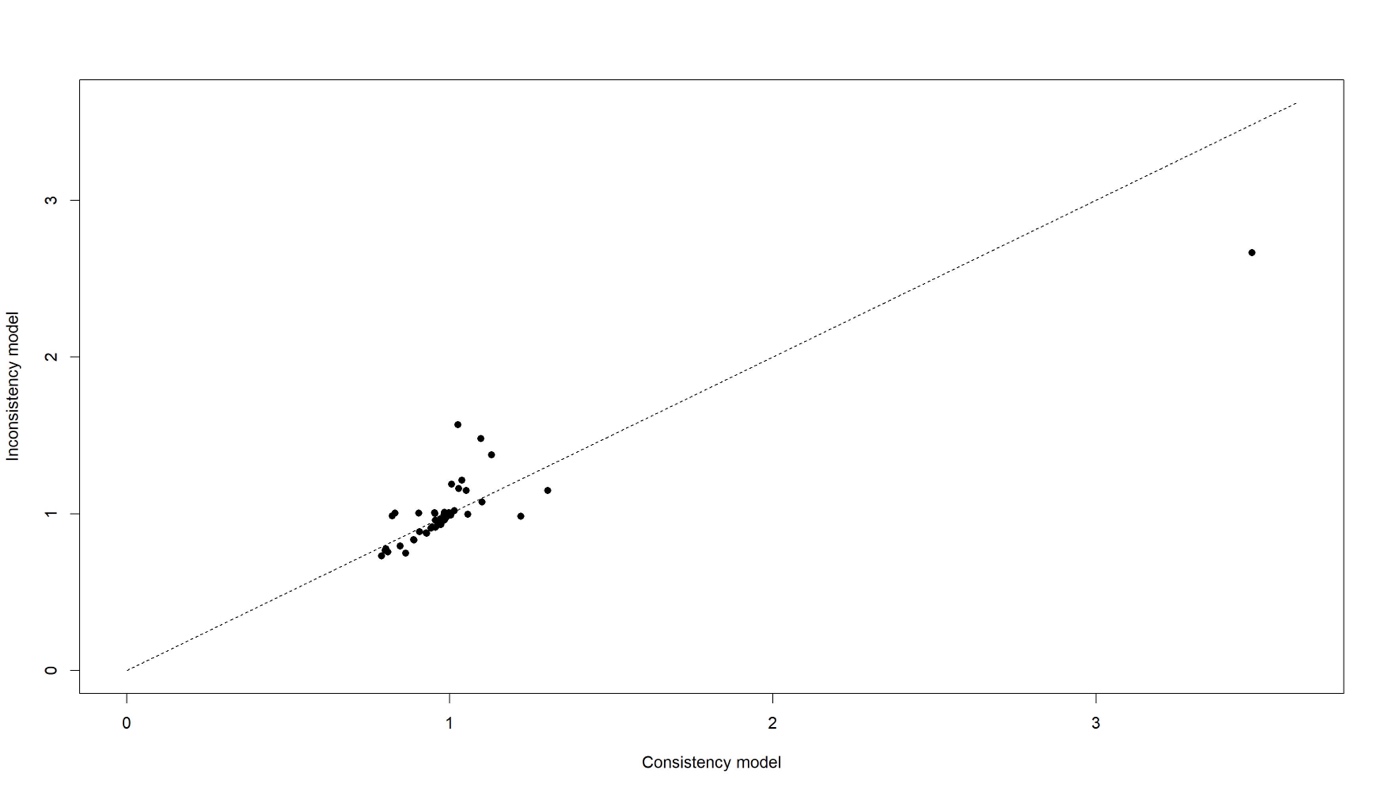


**Supplementary material 6. Figure 1.** Consistency- incostisency plot for marginal gap


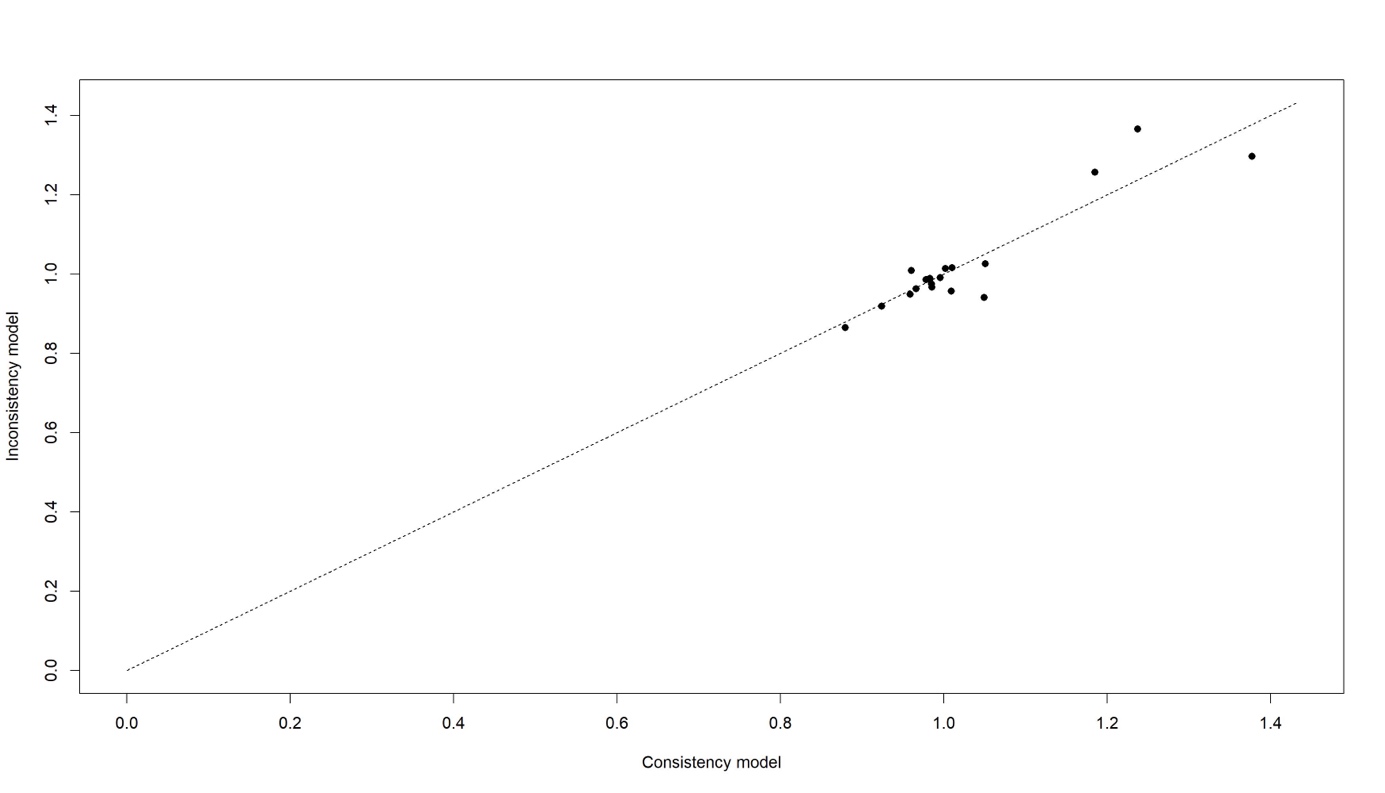


**Supplementary material 6. Figure 2**. Consistency plot for absolute marginal discrepancy


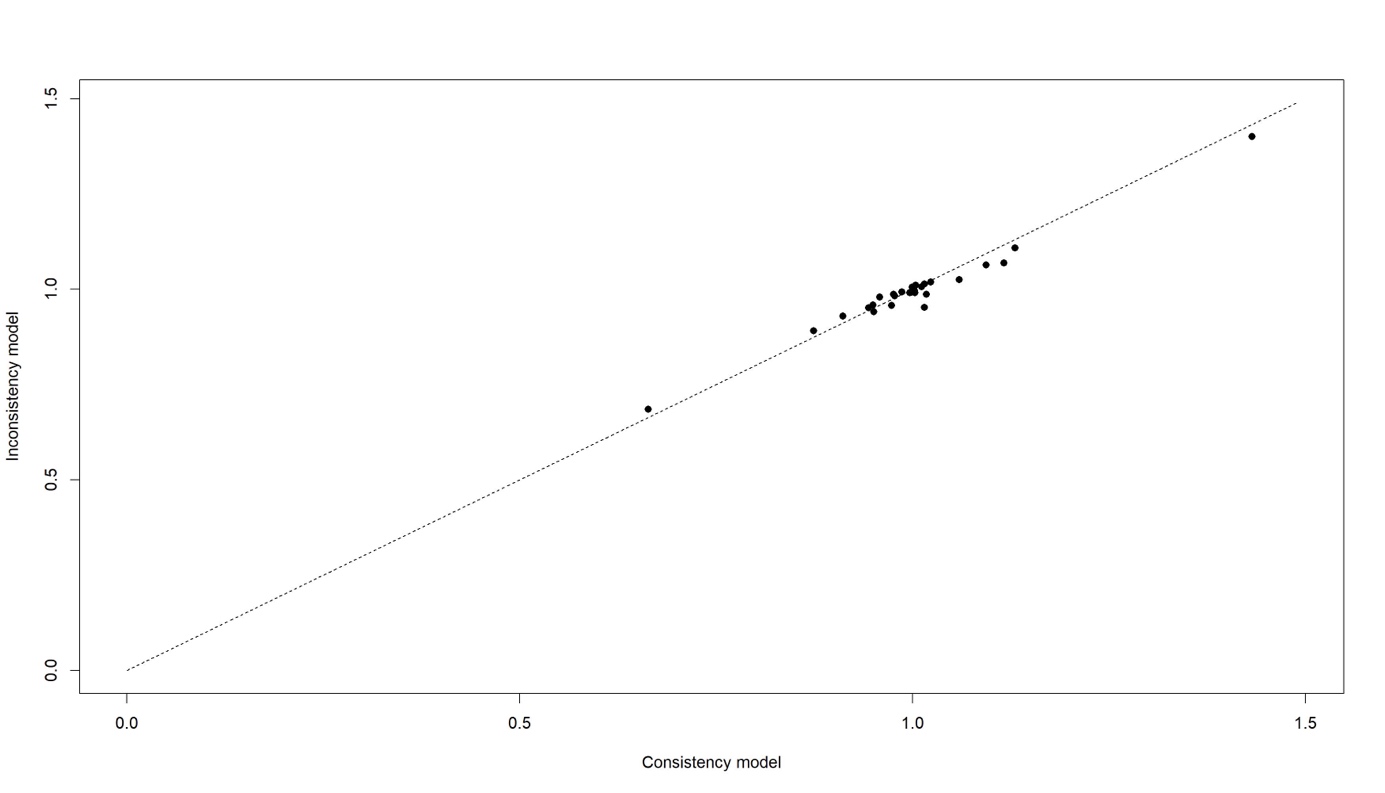


**Supplementary material 6. Figure 3**. Consistency plot for internal gap


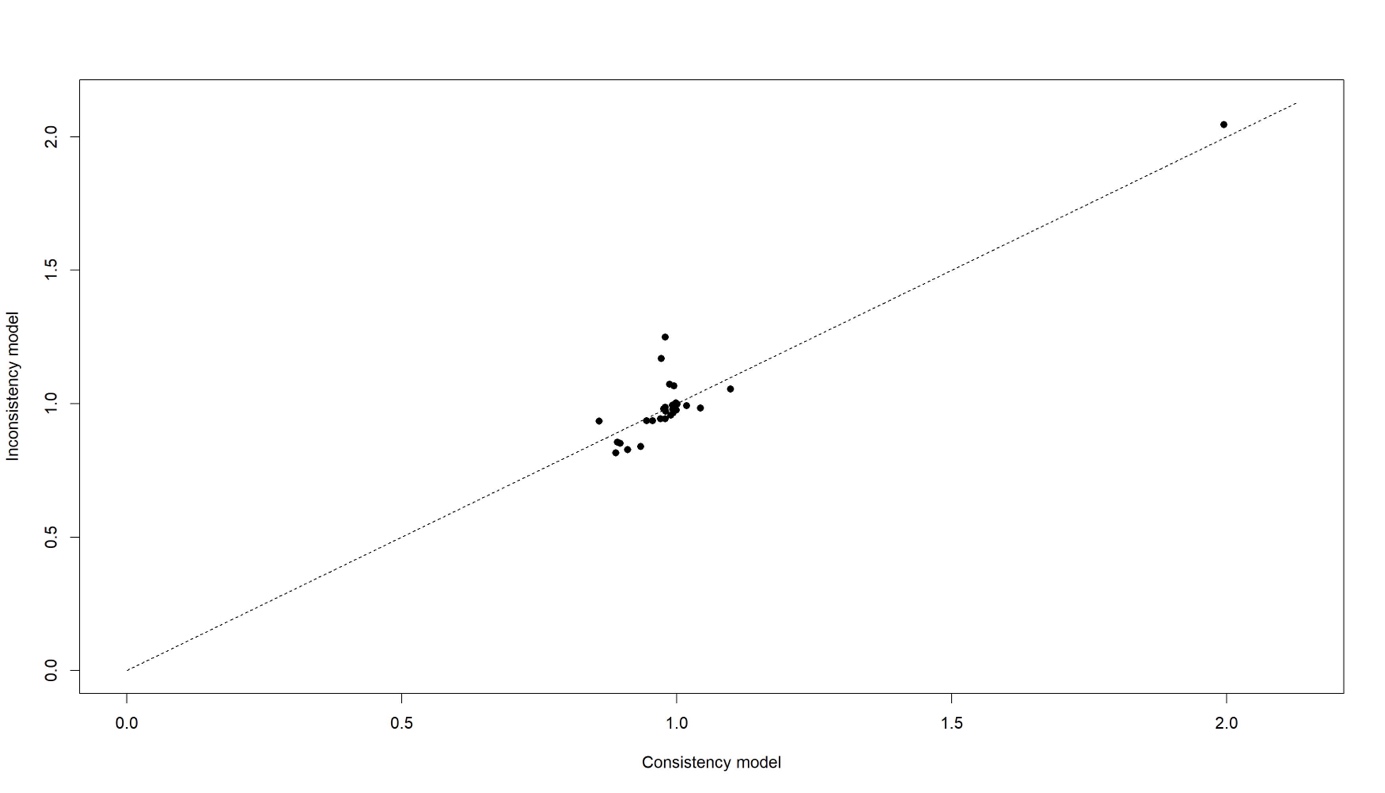


**Supplementary material 6. Figure 4**. Consistency plot for cemented marginal gap


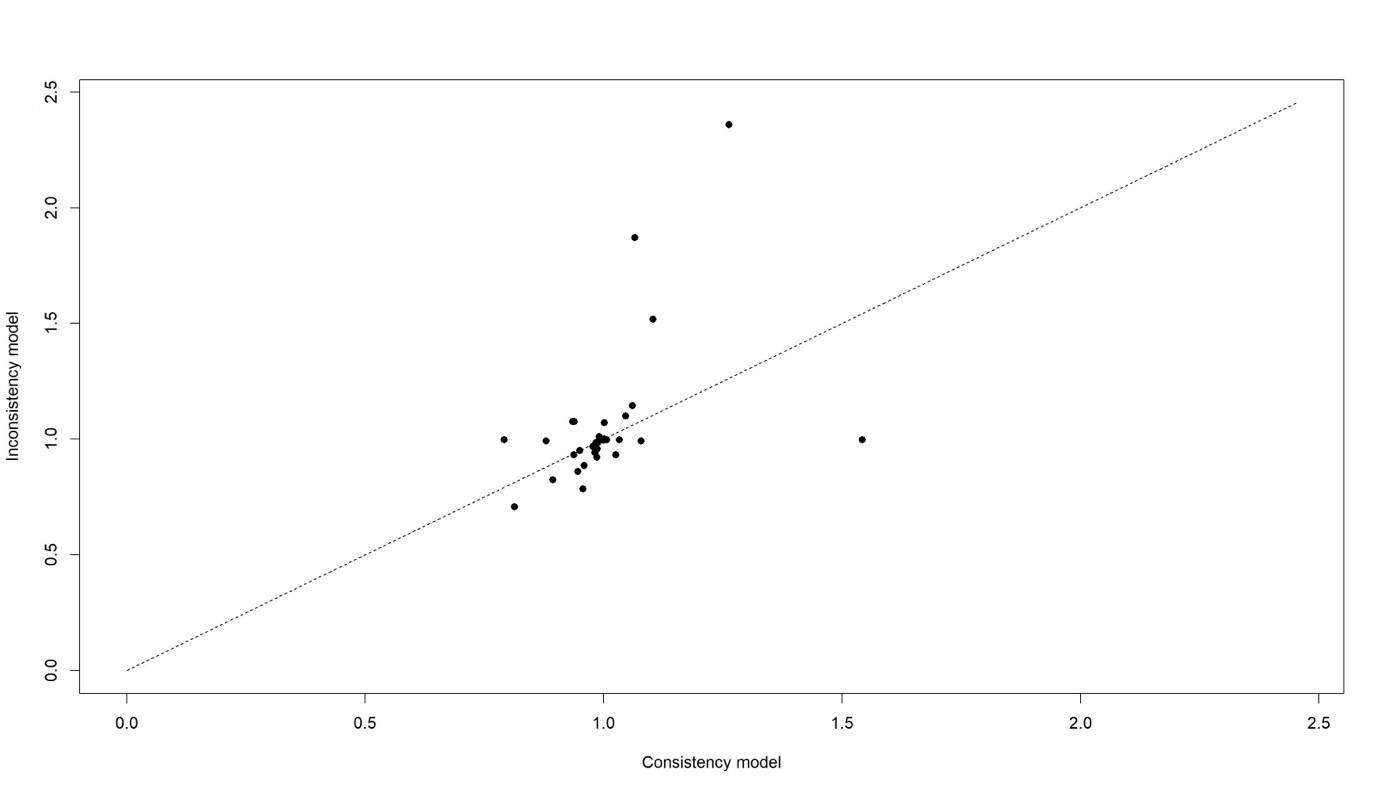


**Supplementary material 6. Figure 5**. Consistency plot for not cemented marginal gap


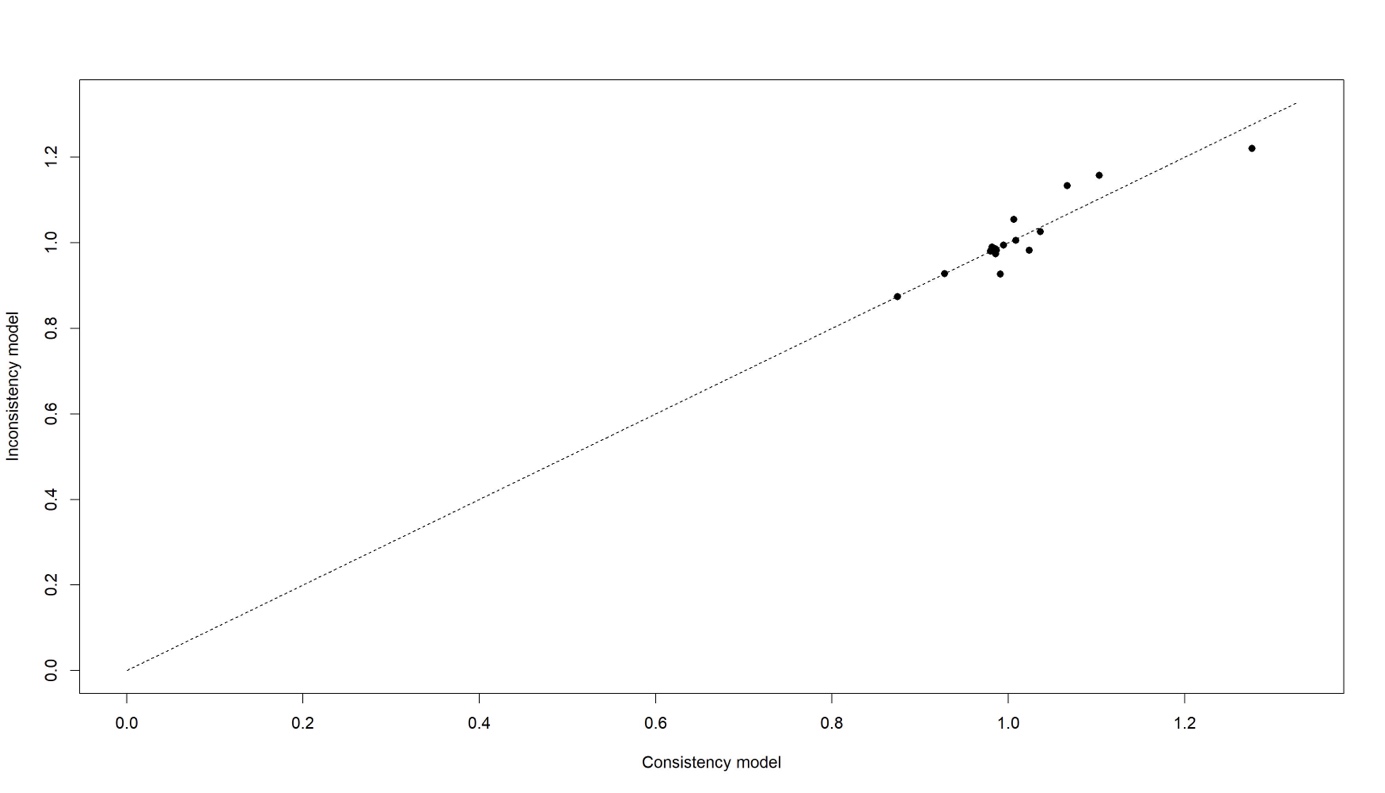


**Supplementary material 6. Figure 6**. Consistency plot for cemented absolute marginal discrepancy


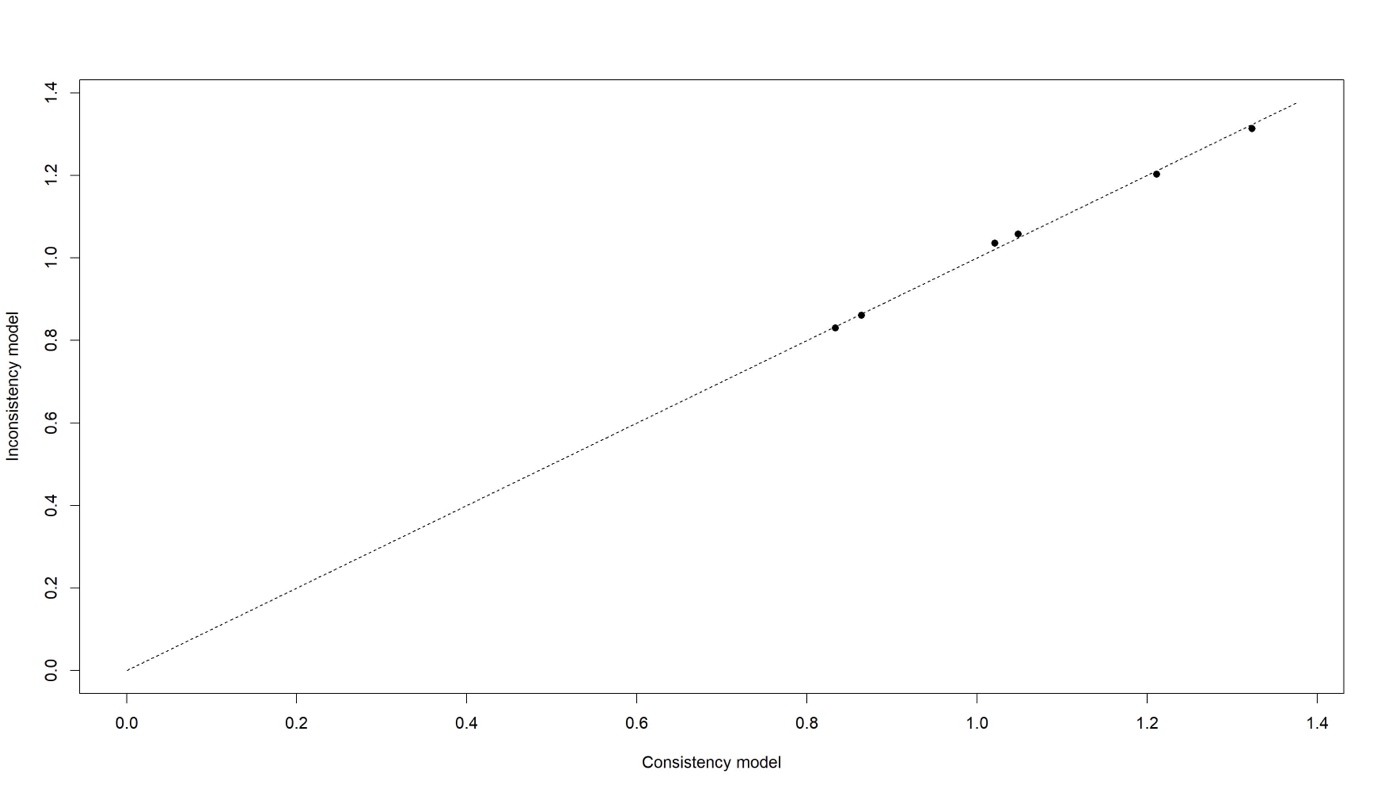


**Supplementary material 6. Figure 7**. Consistency plot for not cemented absolute marginal discrepancy


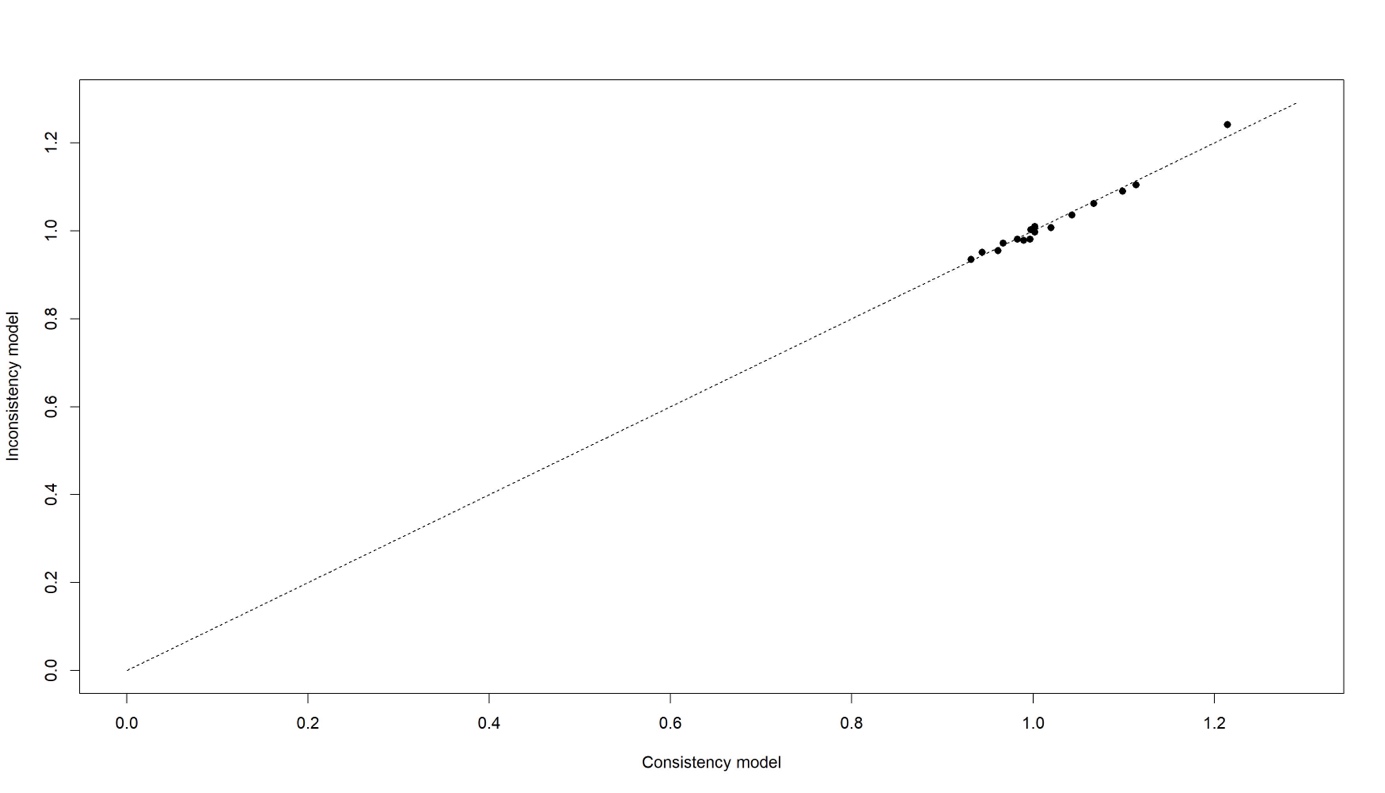


**Supplementary material 6. Figure 8.** Consistency plot for cemented internal gap


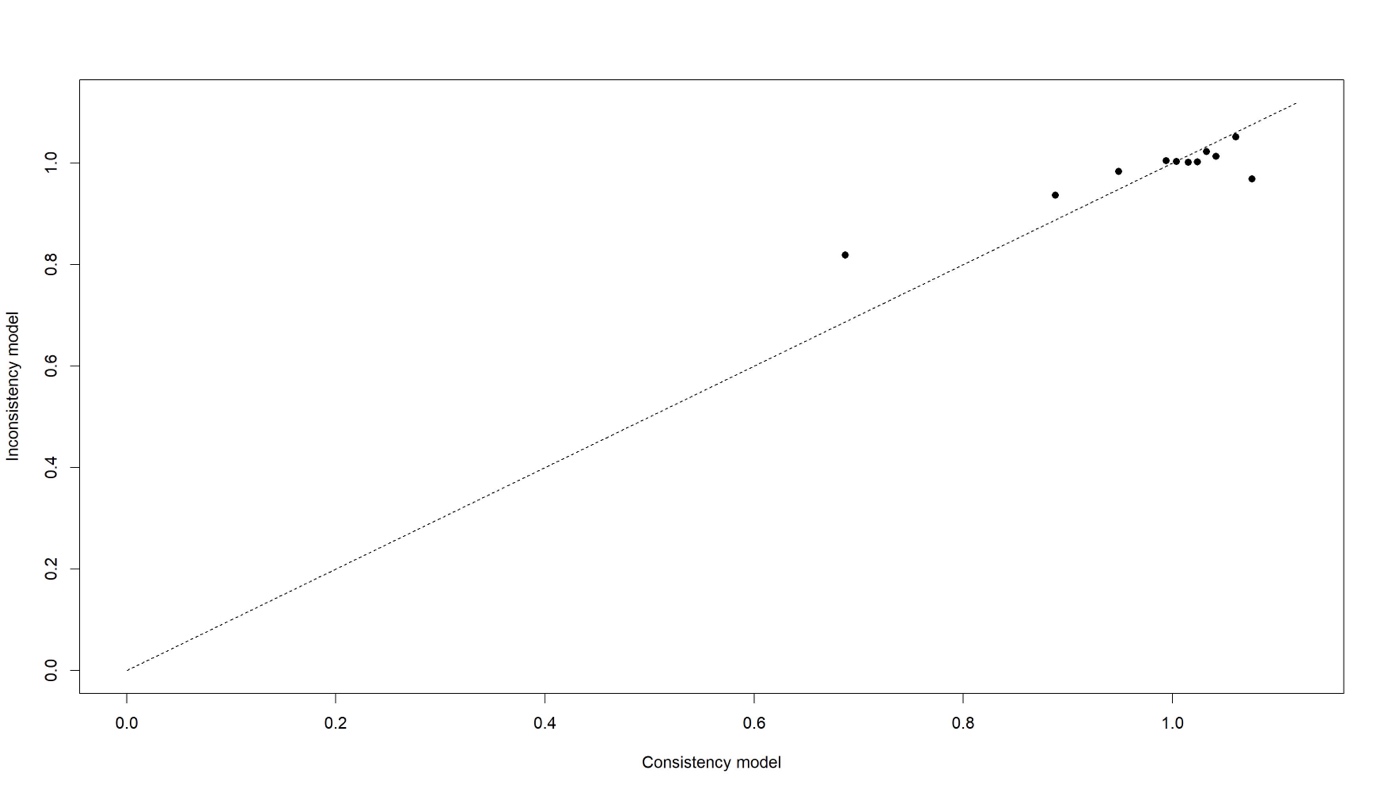


**Supplementary material 6. Figure 9.** Consistency plot for not cemented internal gap


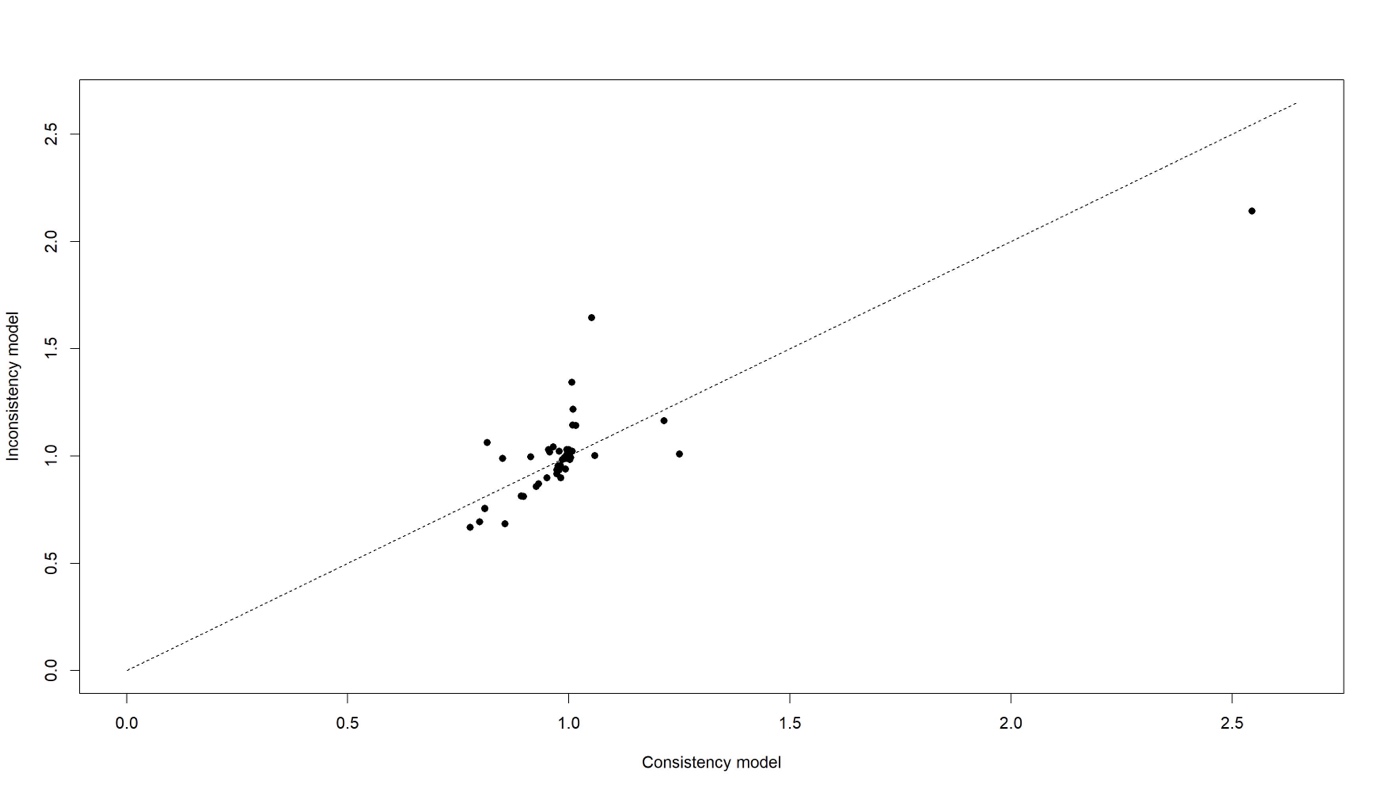


**Supplementary material 6. Figure 10.** Consistency plot for marginal gap of CAD/CAM all-ceramic system


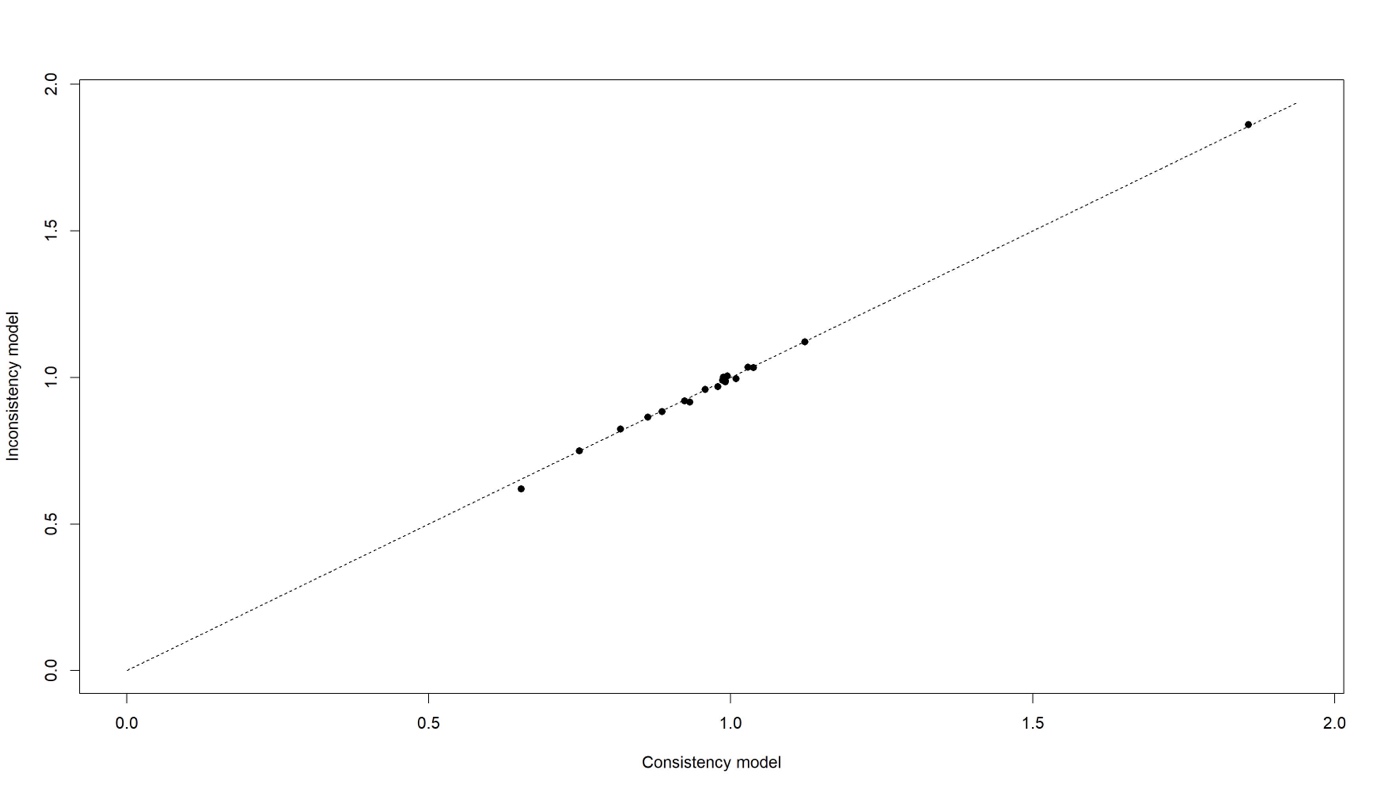


**Supplementary material 6. Figure 11**. Consistency plot for marginal gap of conventional all-ceramic system


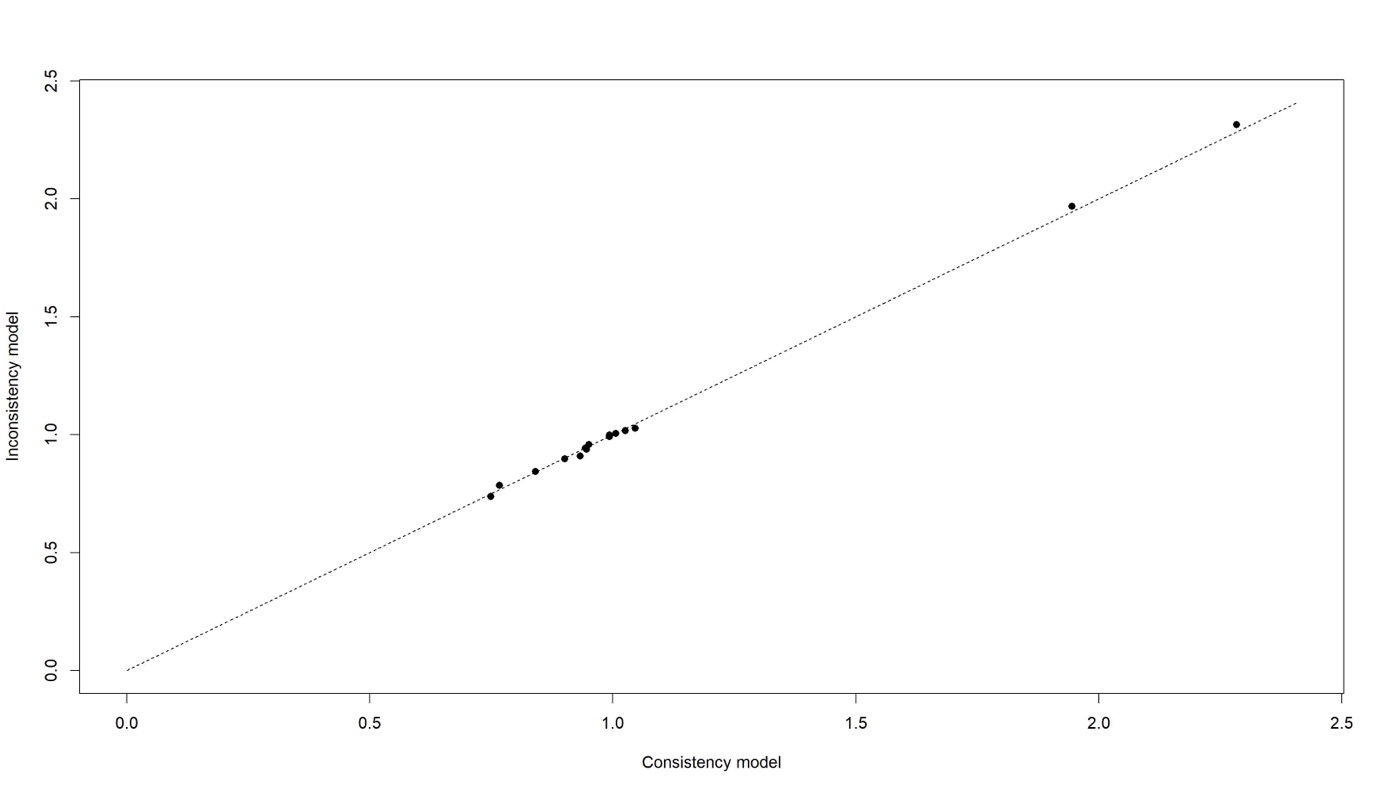


**Supplementary material 6. Figure 12**. Consistency plot for absolute marginal discrepancy of CAD/CAM all-ceramic system


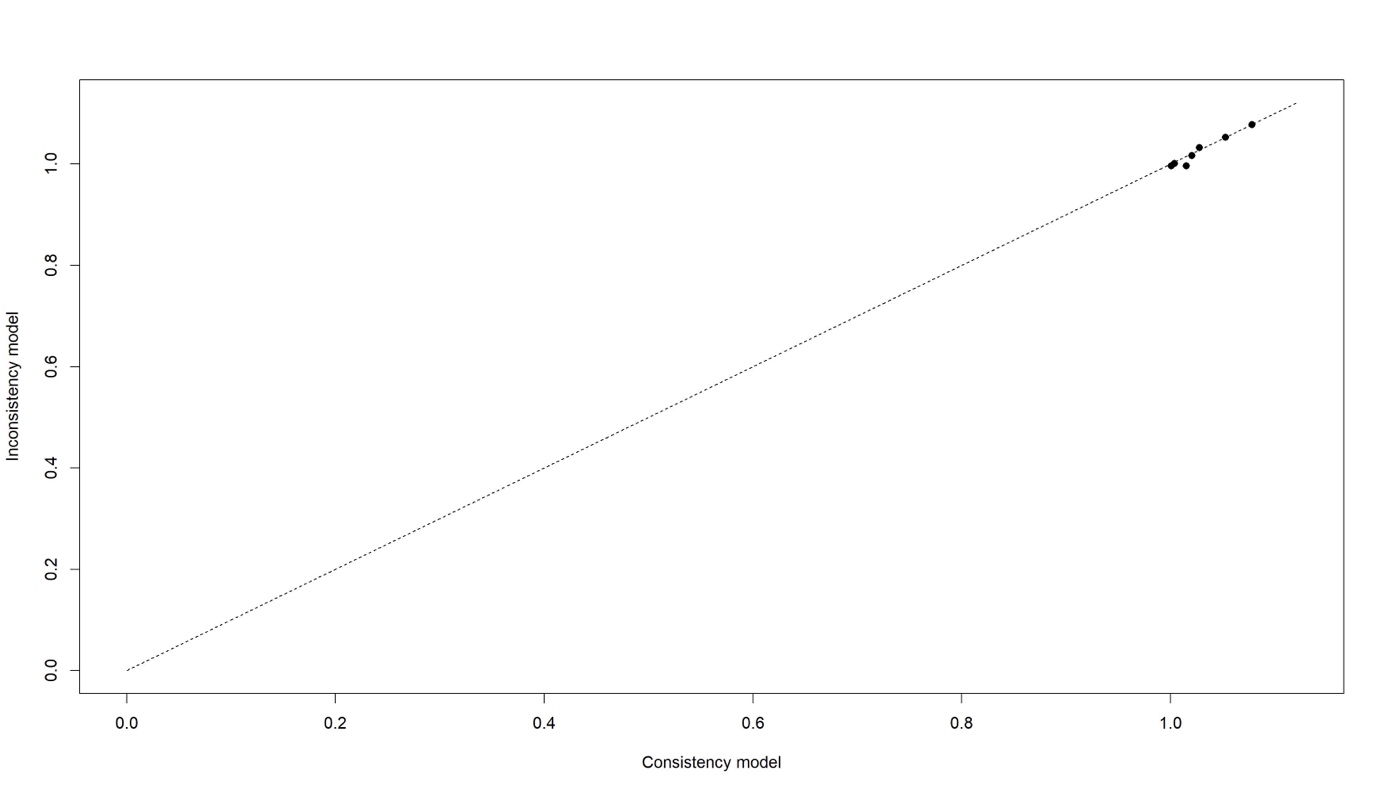


**Supplementary material 6. Figure 13**. Consistency plot for absolute marginal discrepancy of conventional all-ceramic system


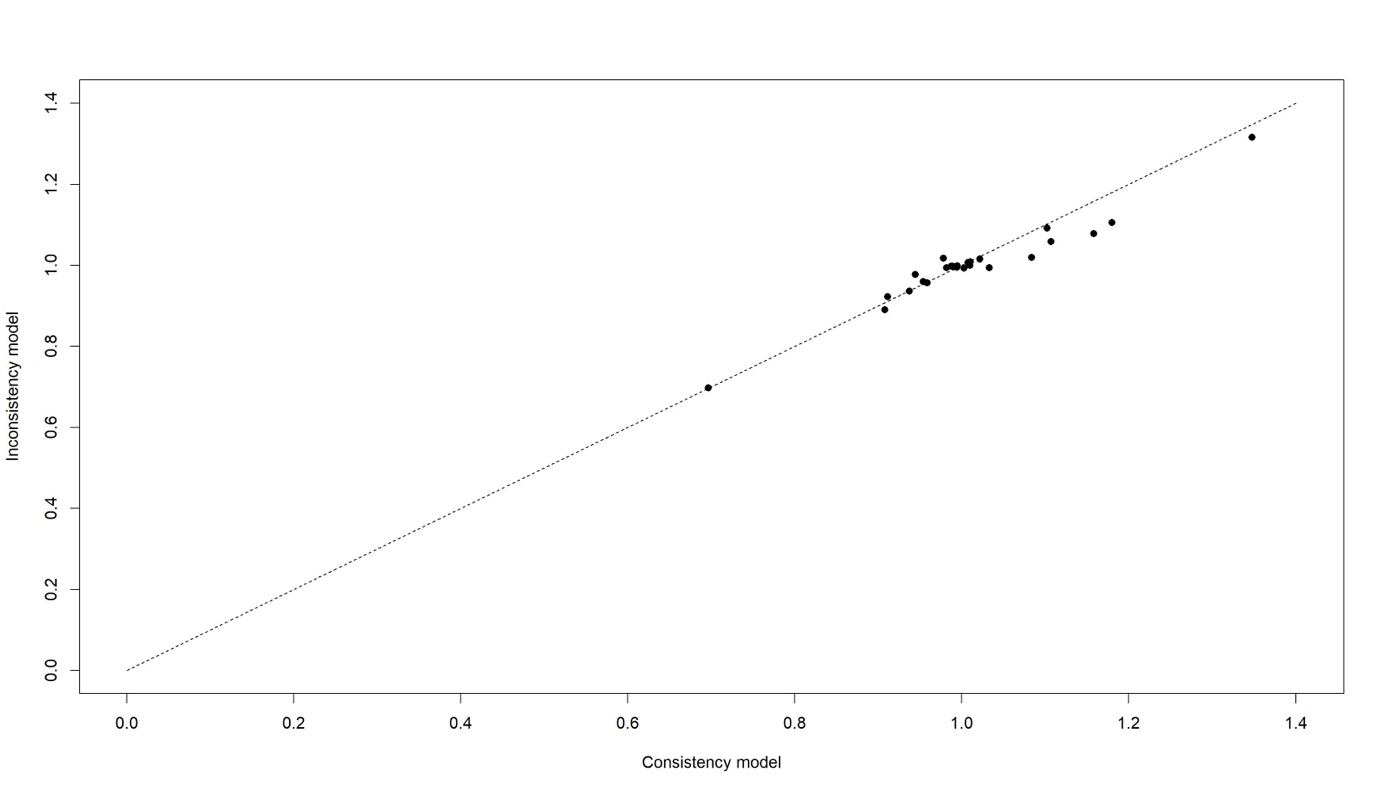


**Supplementary material 6. Figure 14**. Consistency plot for internal gap of CAD/CAM all-ceramic system


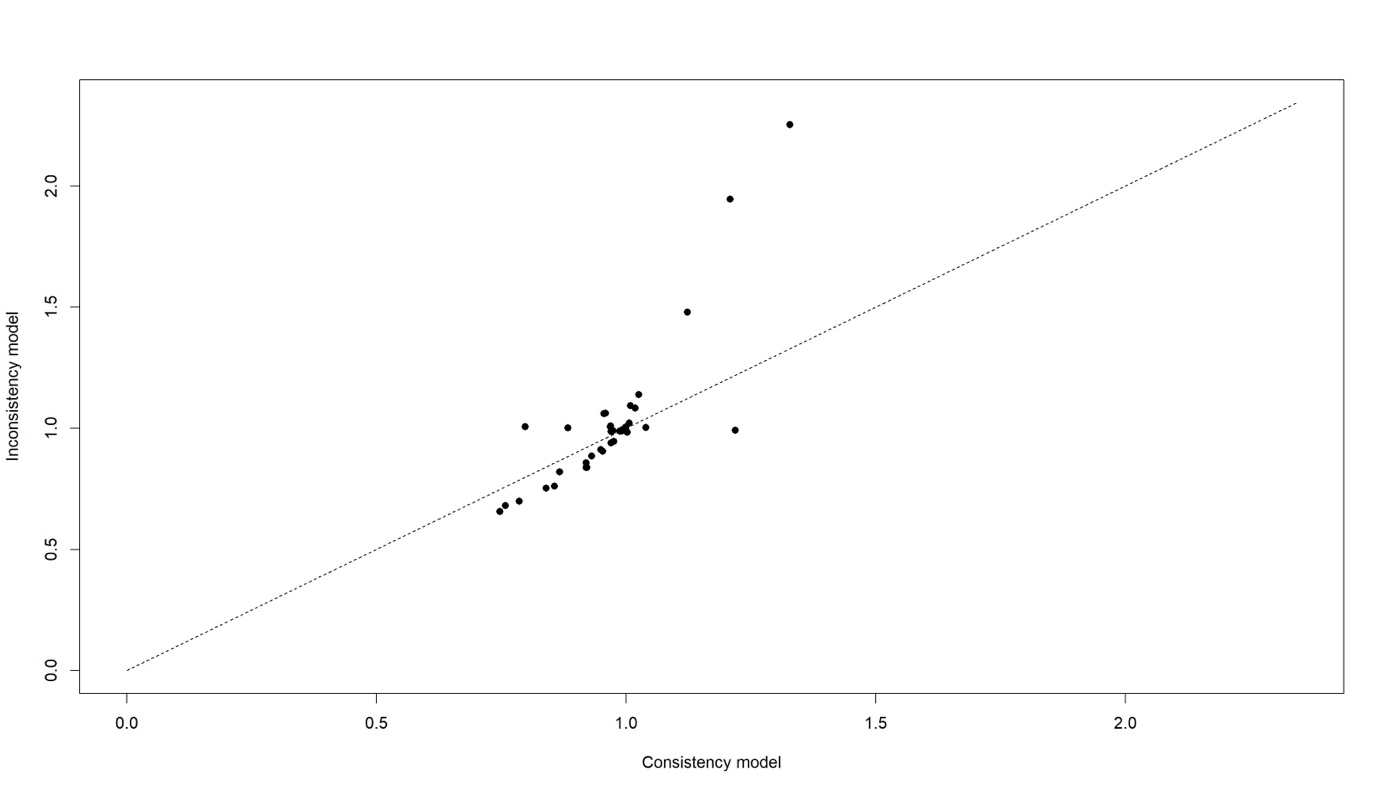


**Supplementary material 6. Figure 15.** Consistency plot for Marginal gap evaluation using the direct view technique

**
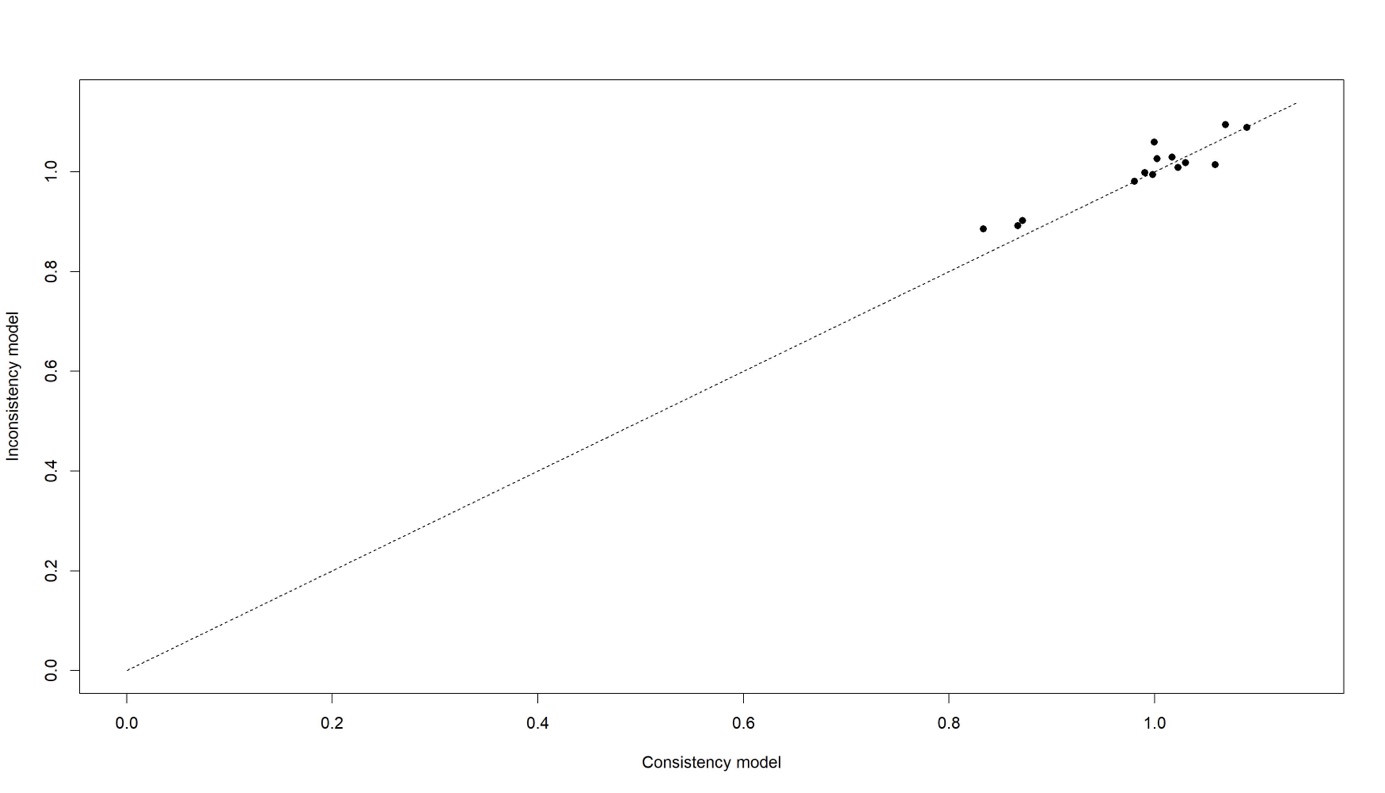
**

**Supplementary material 6. Figure 16.** Consistency plot for Marginal gap evaluation using the cross sectioned technique

**
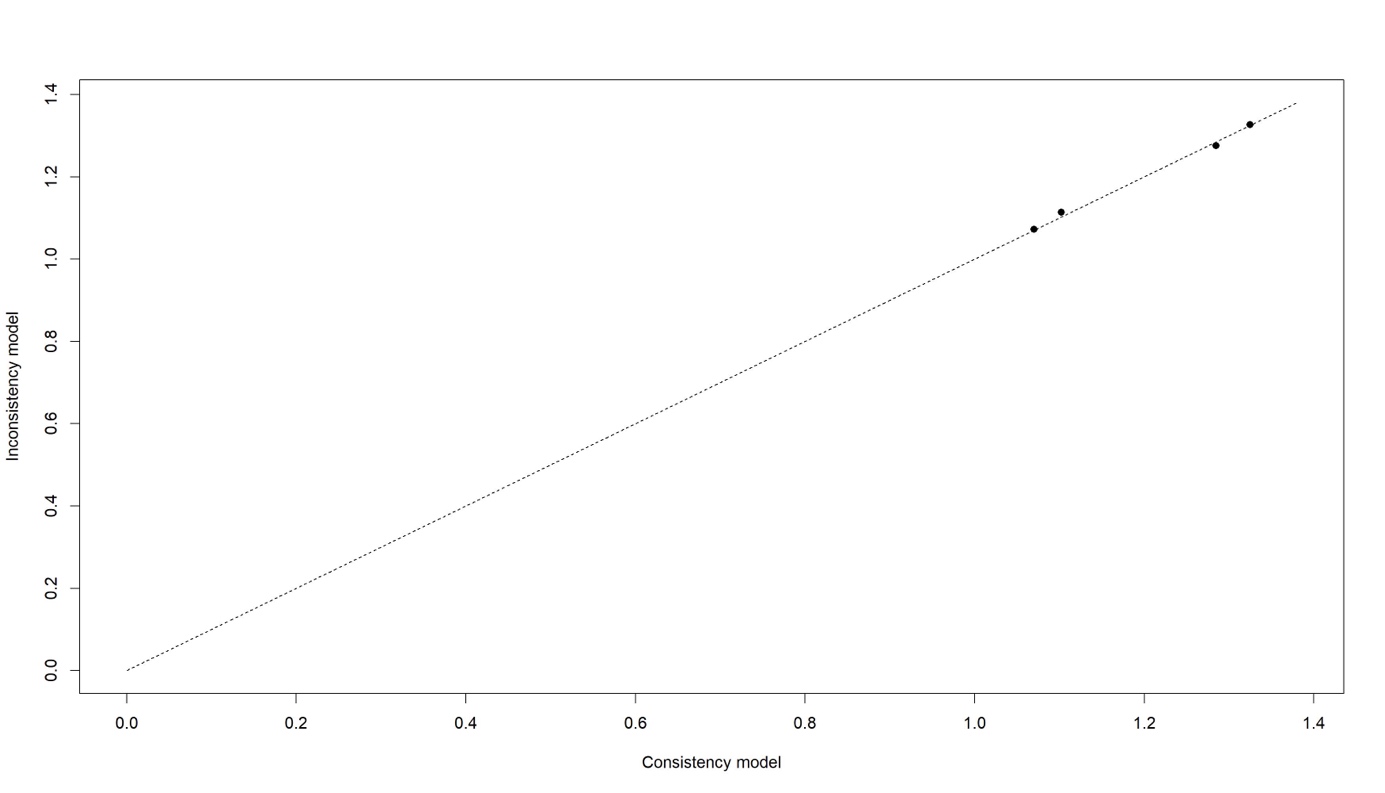
**

**Supplementary material 6. Figure 17.** Consistency plot for Marginal gap evaluation using the silicon replica technique

**
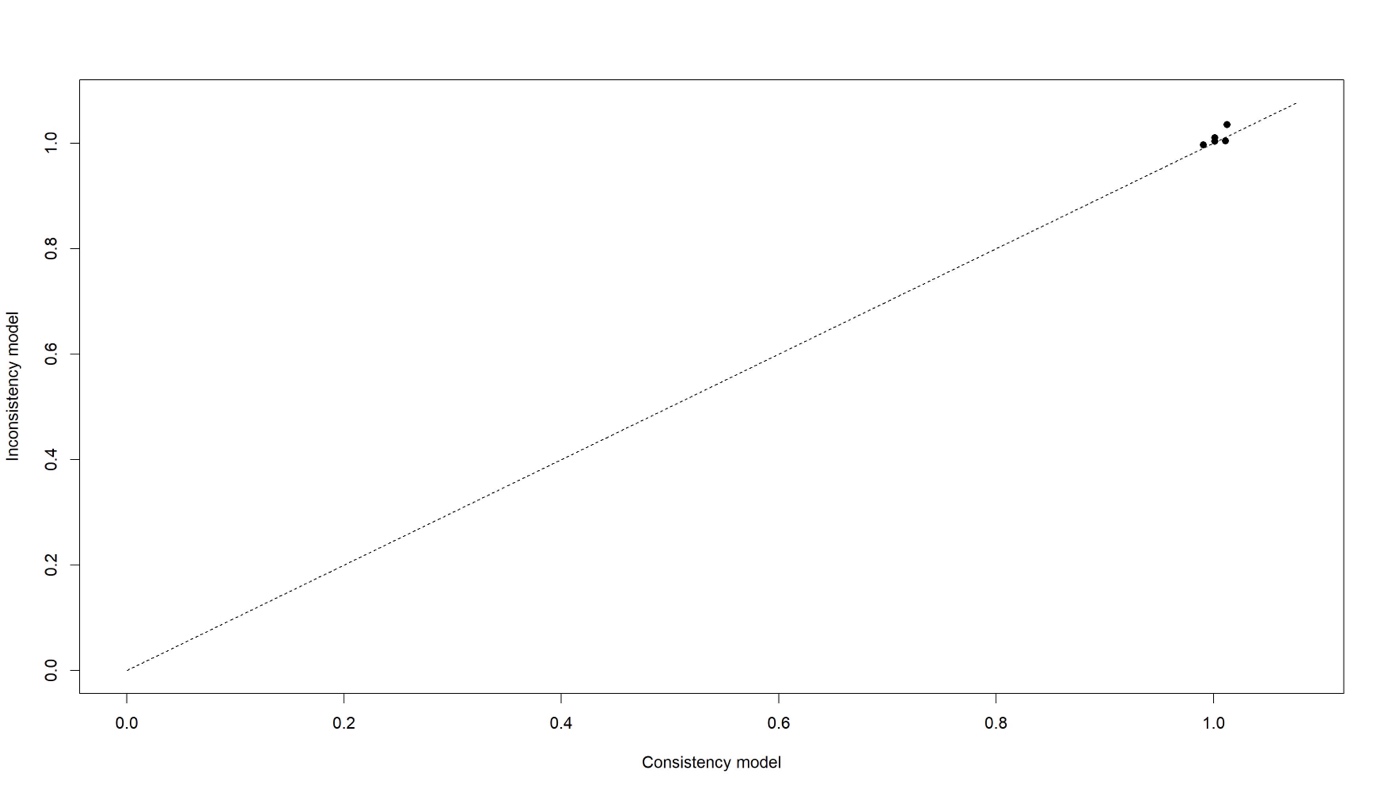
**

**Supplementary material 6. Figure 18.** Consistency plot for Marginal gap evaluation using the micro-CT technique

**
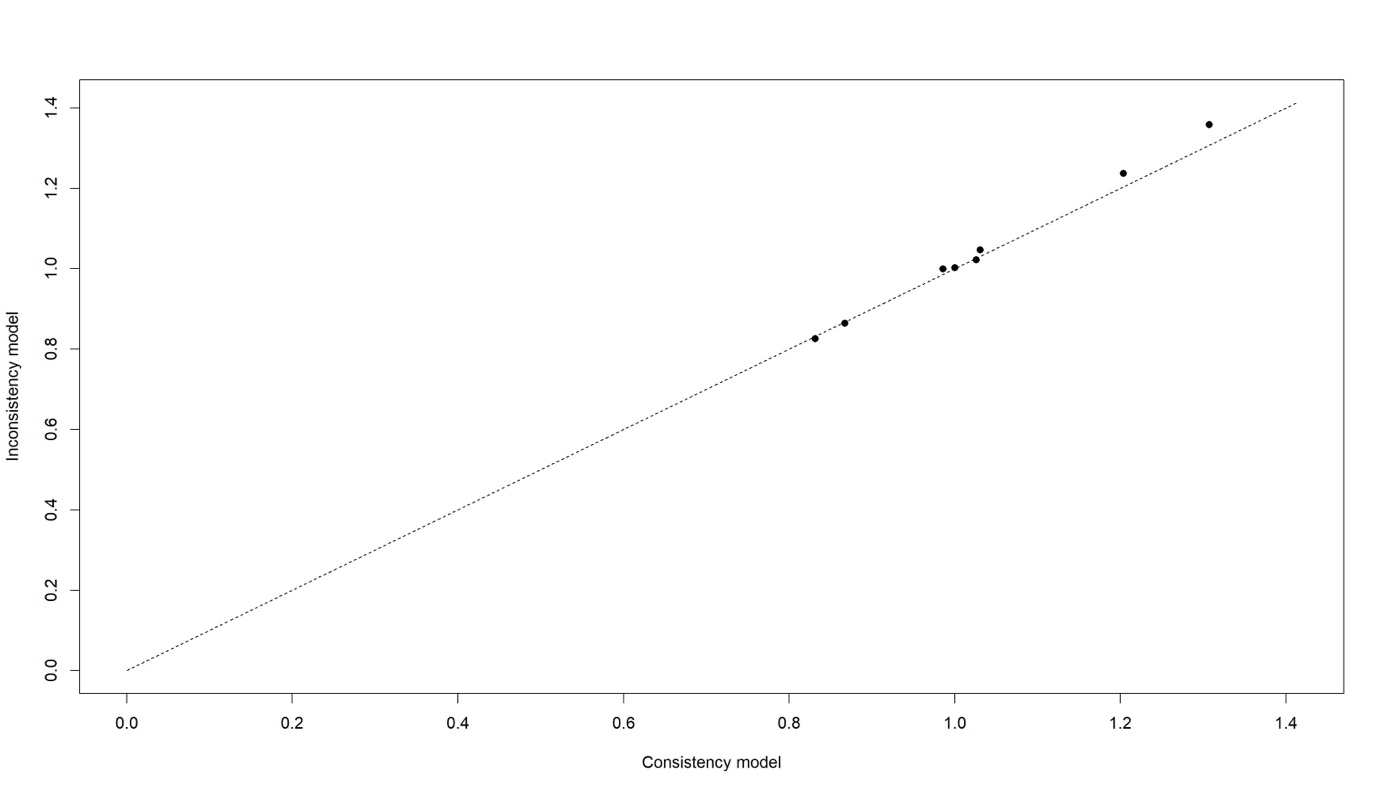
**

**Supplementary material 6. Figure 19.** Consistency plot for Absolute marginal discrepancy evaluation using the direct view technique

**
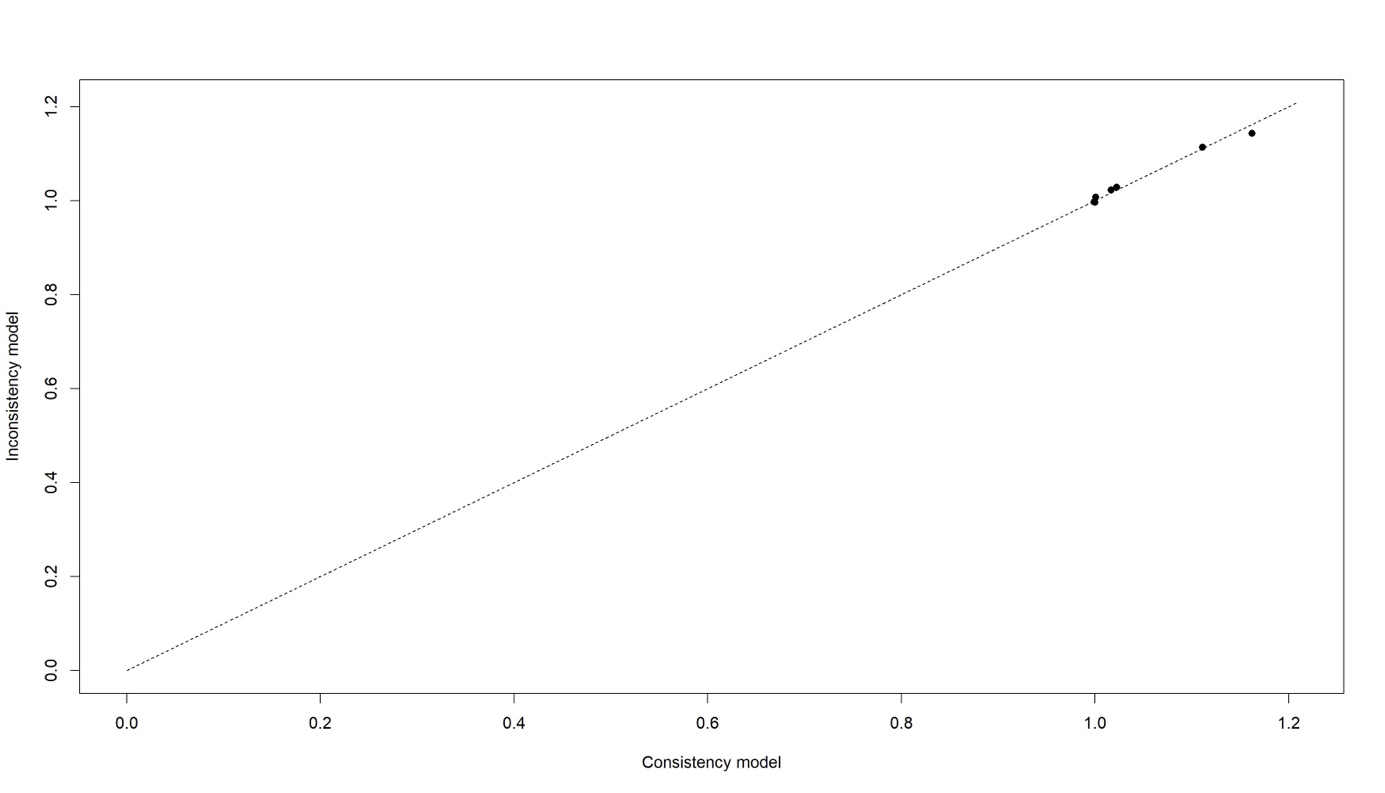
**

**Supplementary material 6. Figure 20.** Consistency plot for Absolute marginal discrepancy evaluation using the cross sectioned technique

**
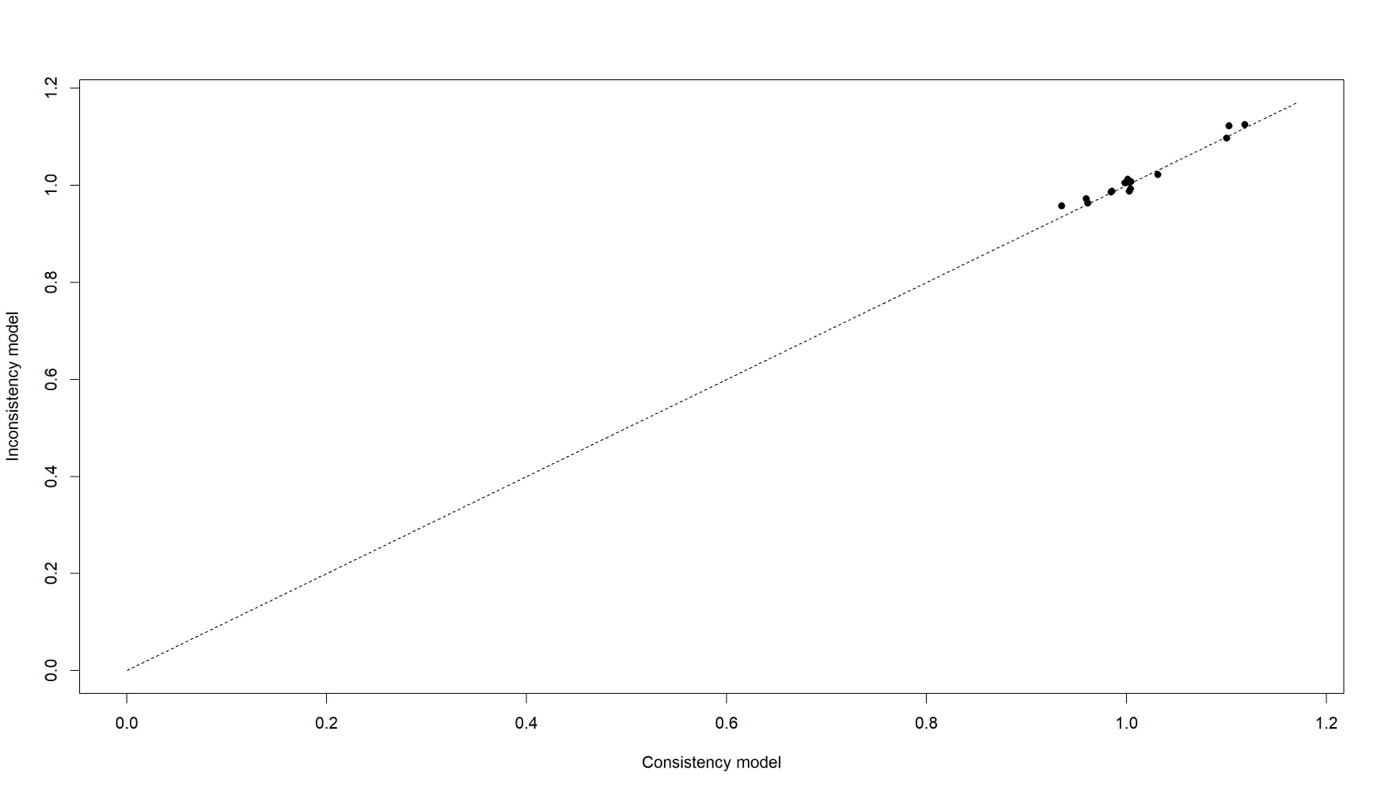
**

**Supplementary material 6. Figure 21.** Consistency plot for Internal gap evaluation using the cross sectioned technique

**
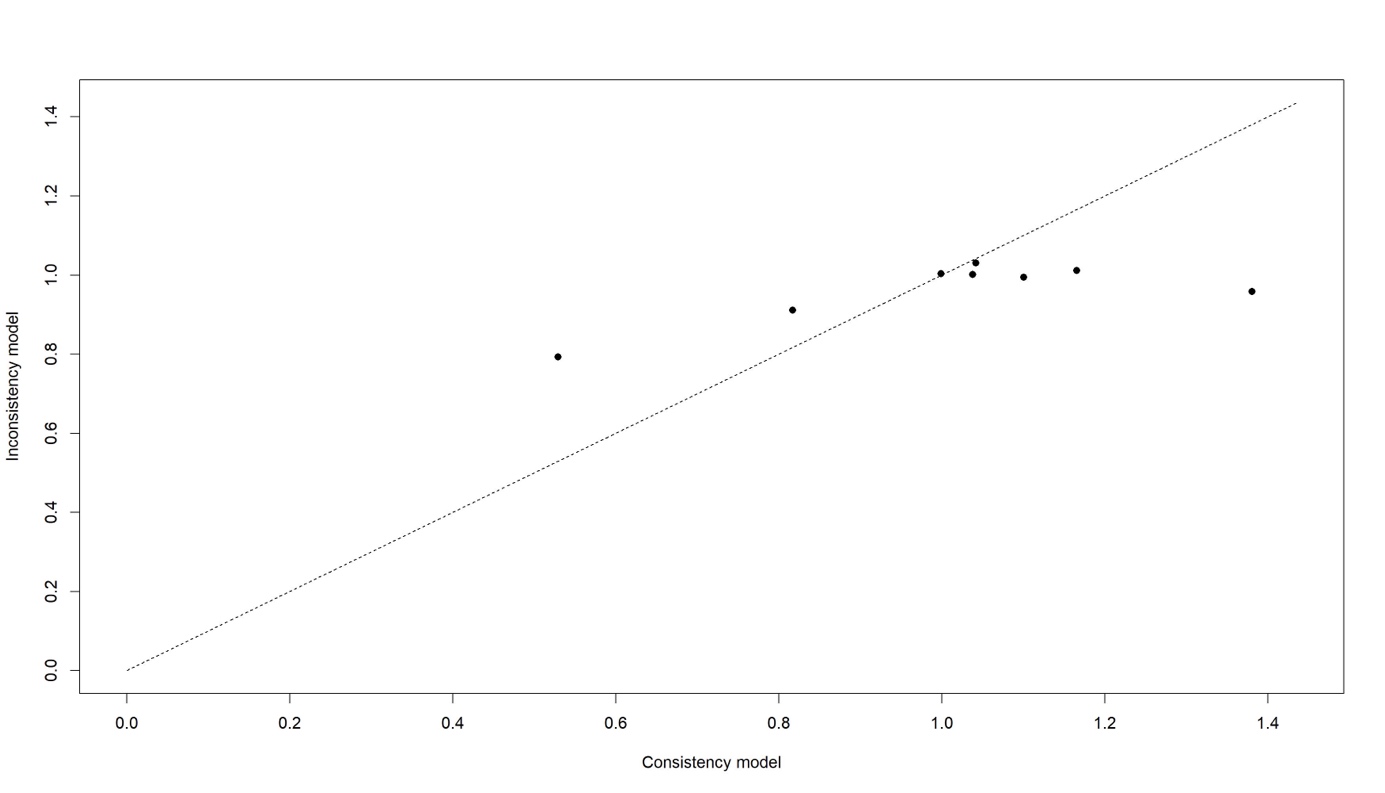
**

**Supplementary material 6. Figure 22.** Consistency plot for Internal gap evaluation using the silicon replica technique

**
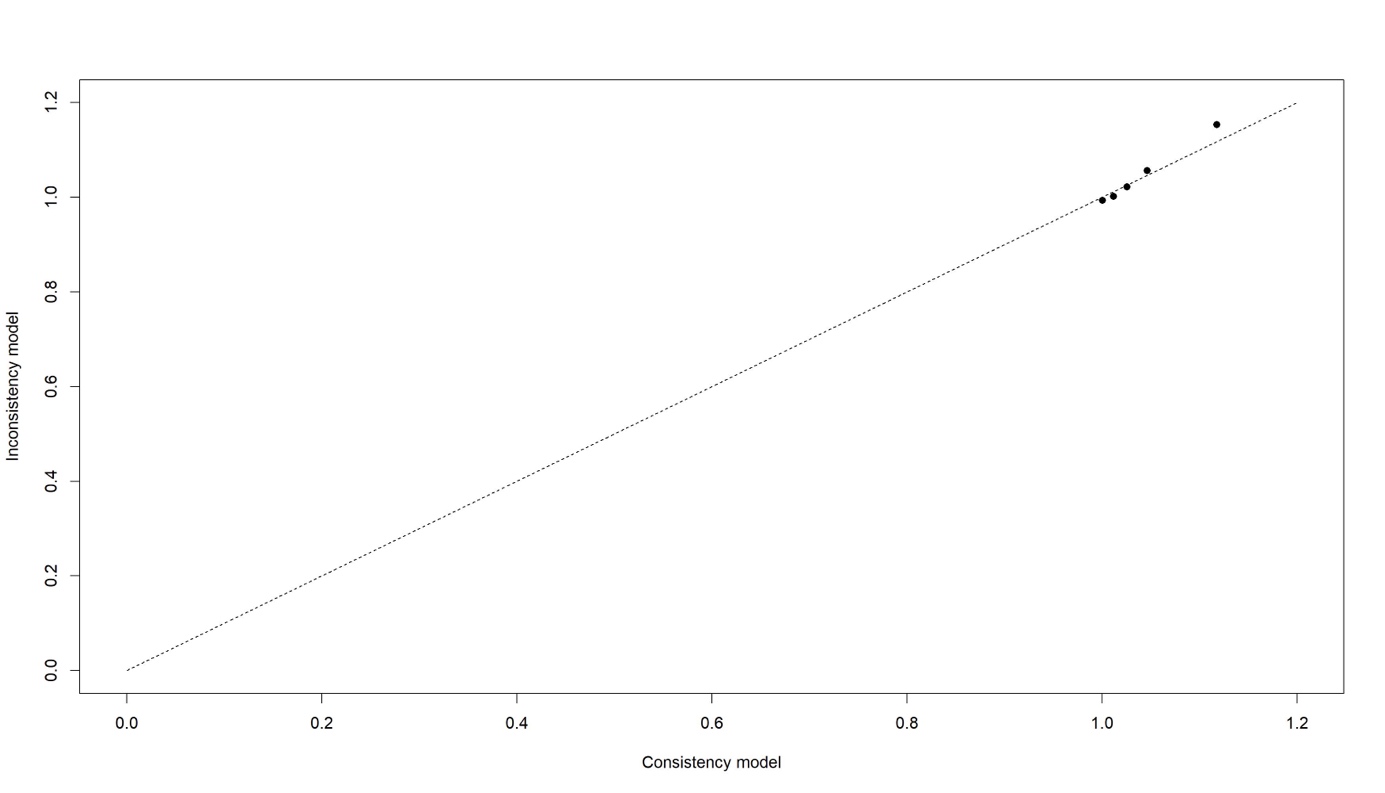
**

**Supplementary material 6. Figure 23.** Consistency plot for Internal gap evaluation using the micro-CT technique

**
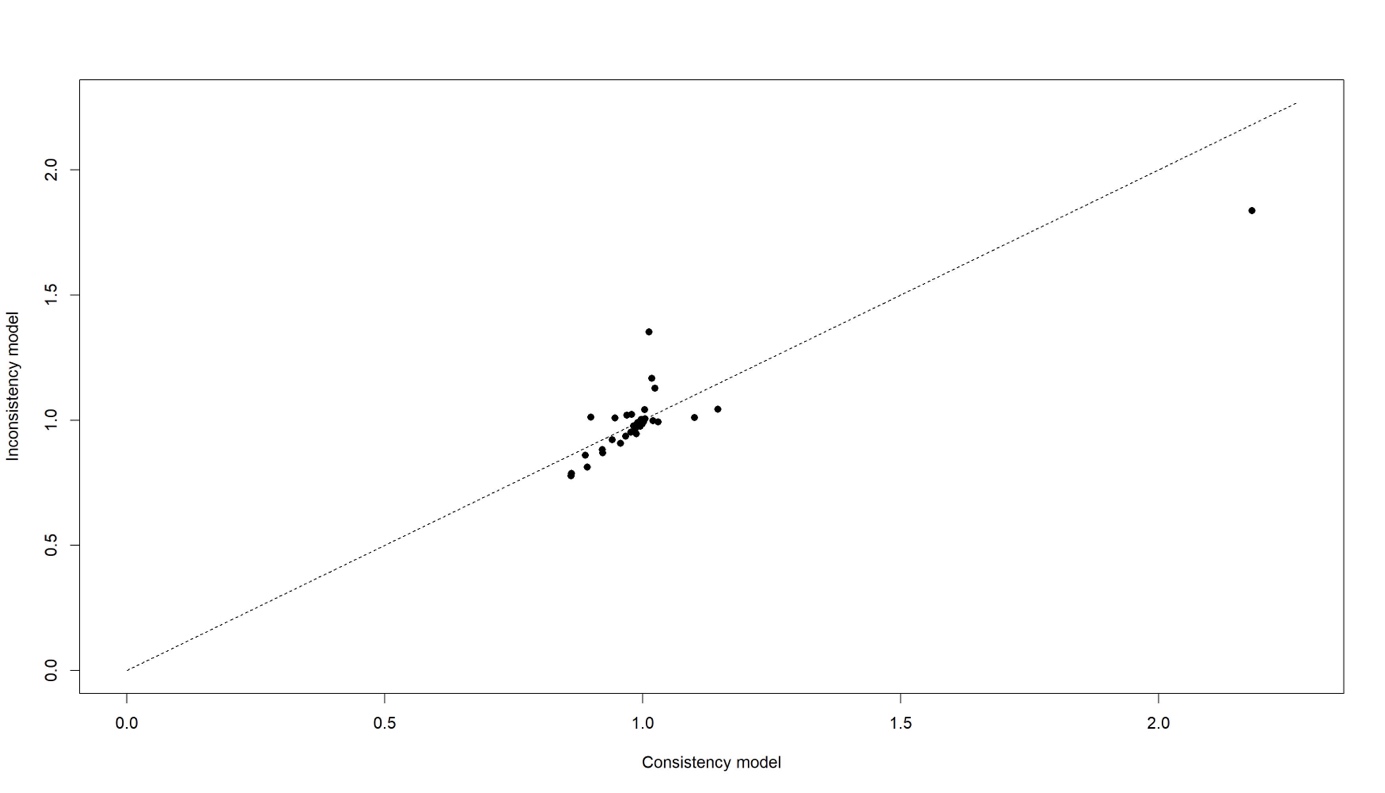
**

**Supplementary material 6. Figure 24.** Consistency plot for Marginal gap of crowns, endocrowns

**
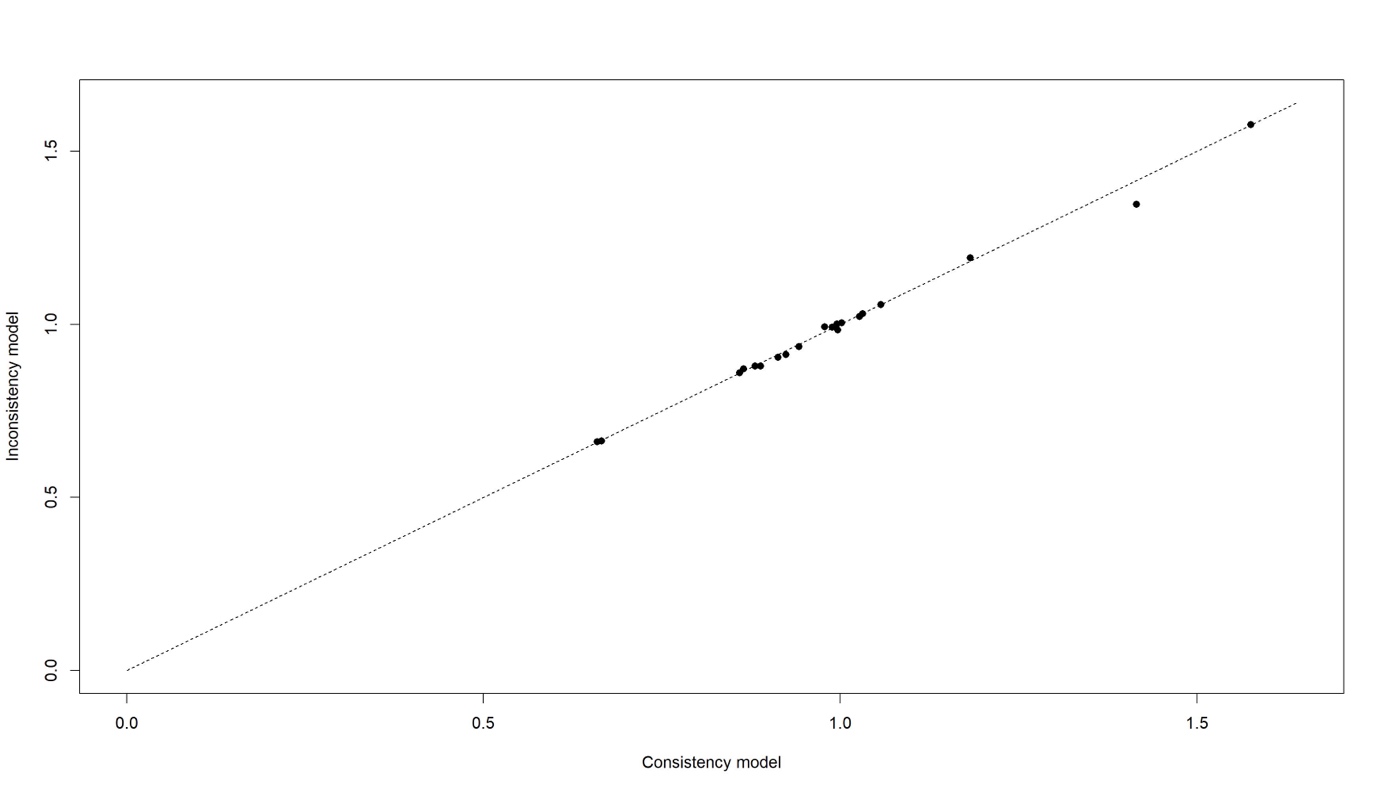
**

**Supplementary material 6. Figure 25.** Consistency plot for Marginal gap of copings

**
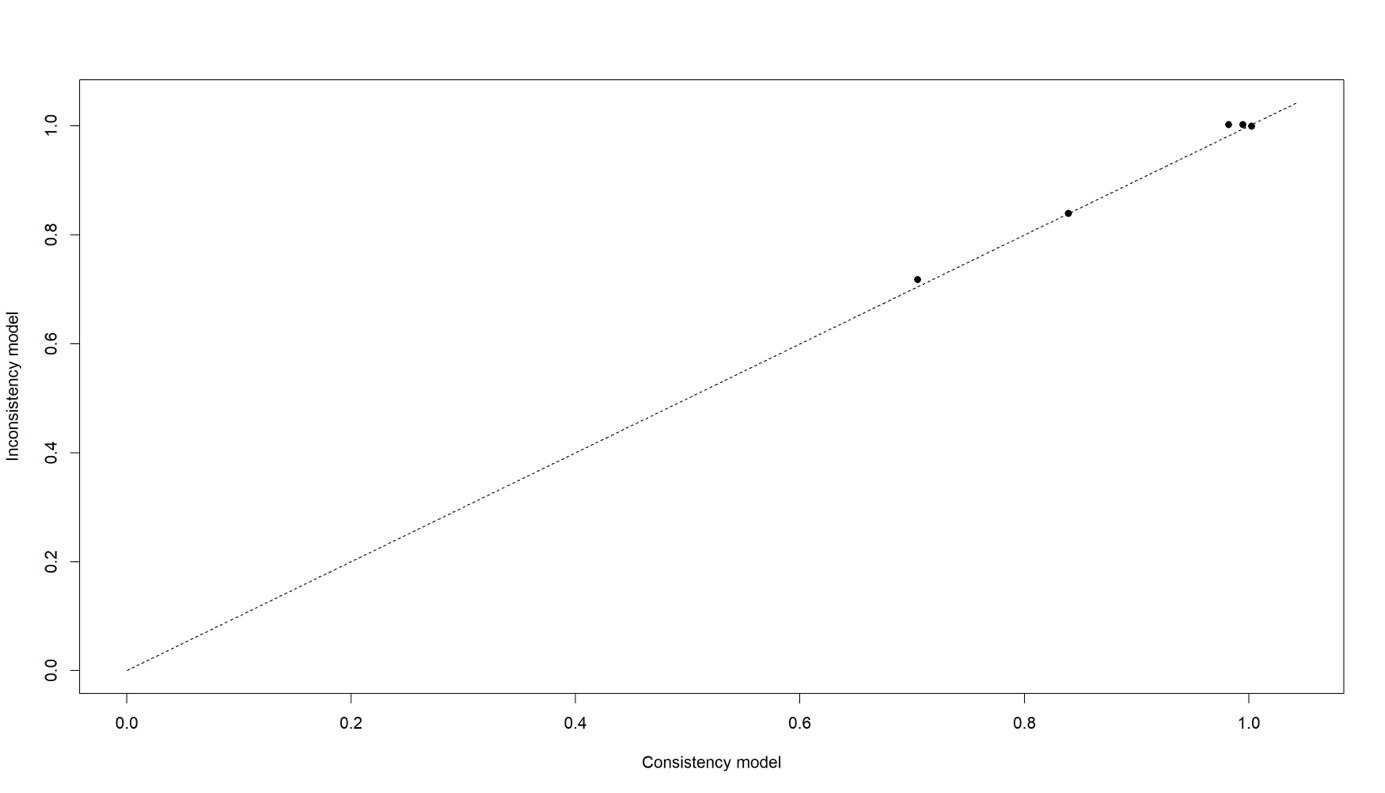
**

**Supplementary material 6. Figure 26.** Consistency plot for Marginal gap of veneers

**
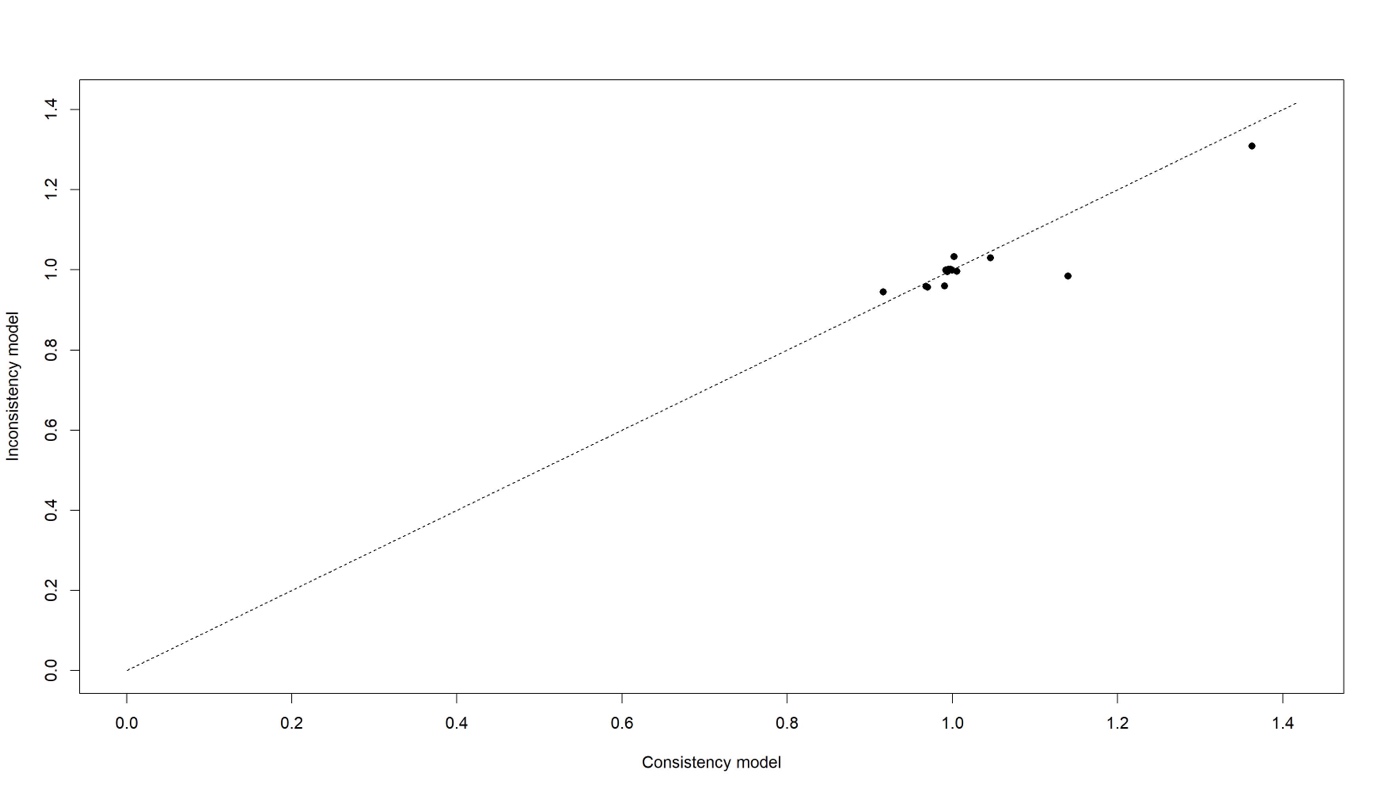
**

**Supplementary material 6. Figure 27.** Consistency plot for Absolute marginal discrepancy of crowns, endorowns

**
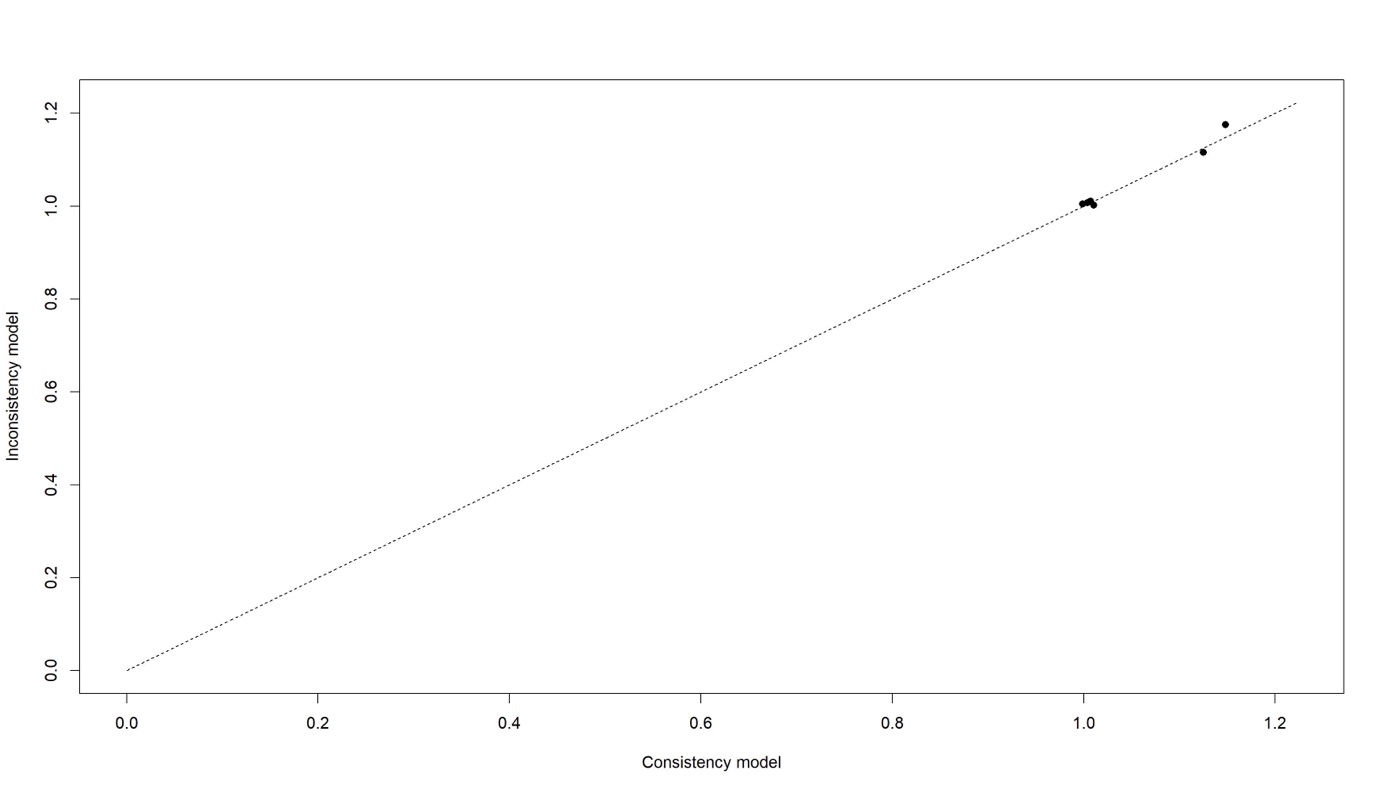
**

**Supplementary material 6. Figure 28.** Consistency plot for Absolute marginal discrepancy of copings

**
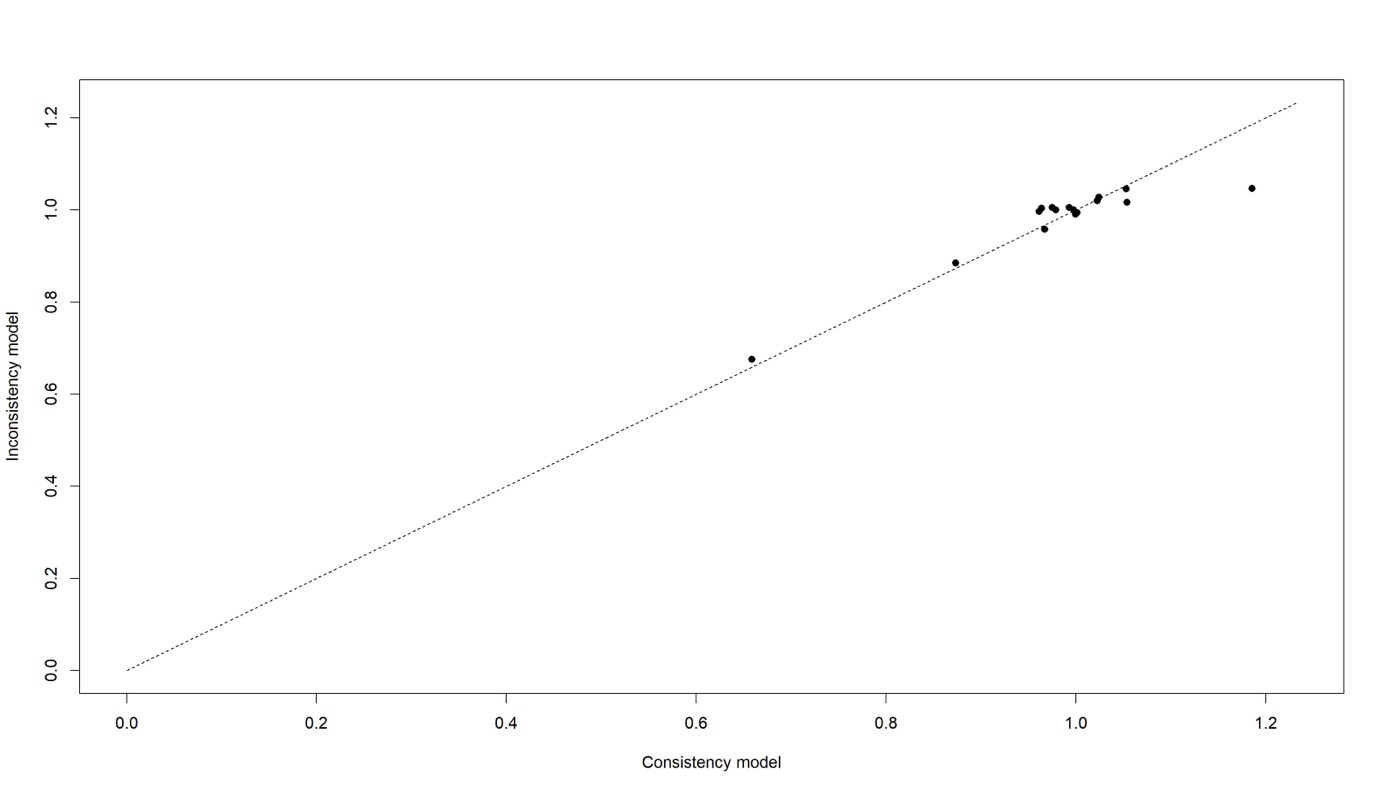
**

**Supplementary material 6. Figure 29.** Consistency plot for Internal gap of crowns, endocrowns**
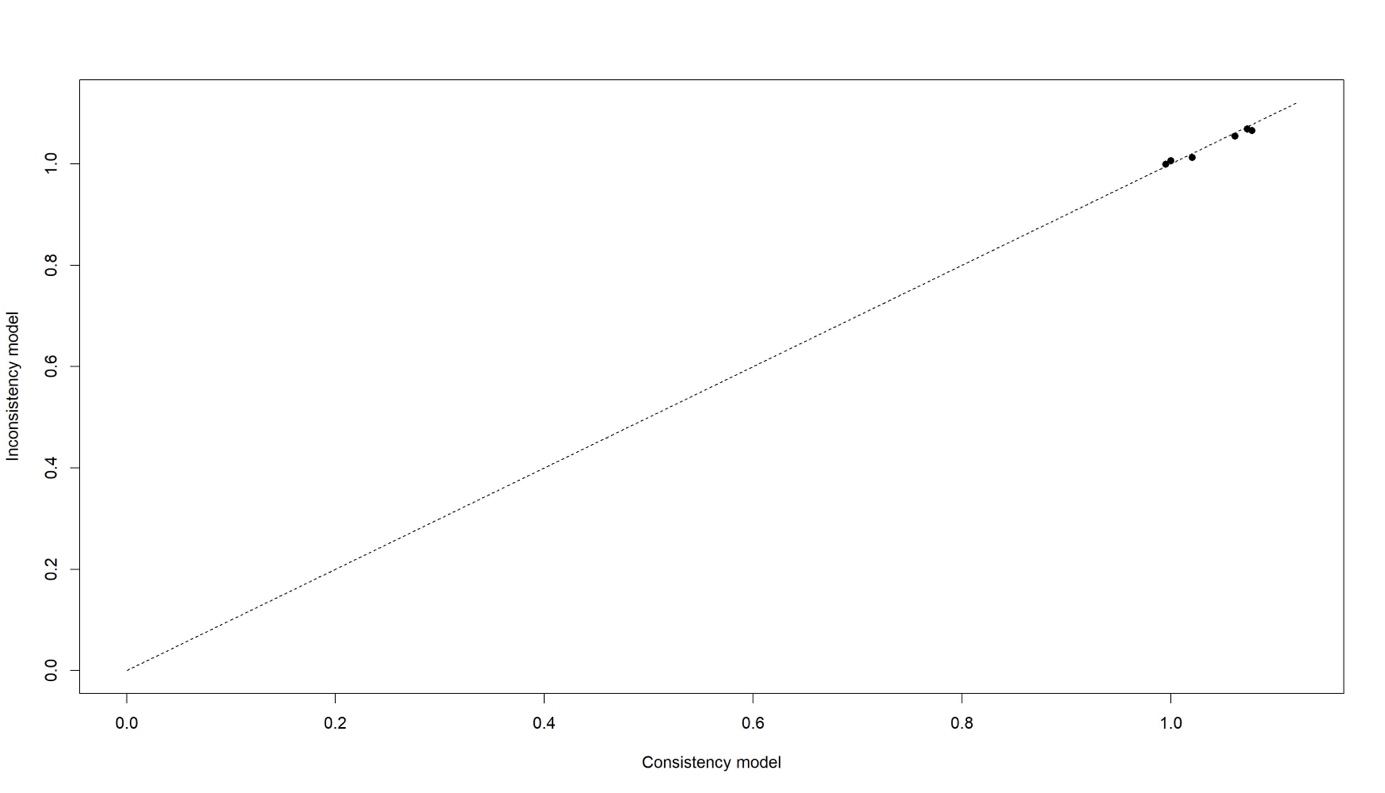
**

**Supplementary material 6. Figure 30.** Consistency plot for Internal gap of copings

**
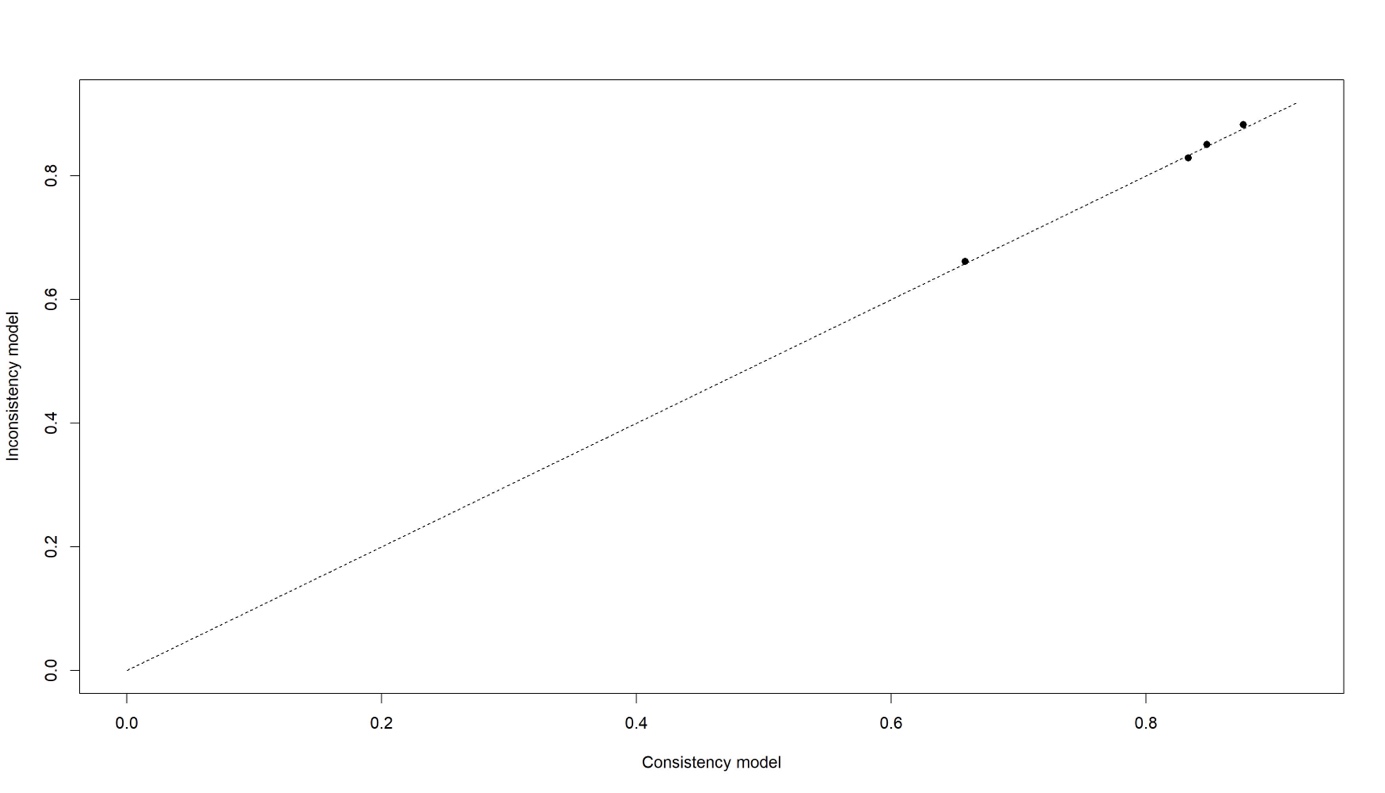
**

**Supplementary material 6. Figure 31.** Consistency plot for Internal gap of veneers
