# Supplementary material for: Impact of finish line designs on the adaptation of ceramic fixed dental prostheses: a systematic review and network meta-analysis
Source: BMC Oral Health. 2025 Jul 3;25:1085. doi: 10.1186/s12903-025-06433-0 (PMC12231902; doi:10.1186/s12903-025-06433-0)
Supplement: Supplementary file 7 — Supplementary Material 7 [file 12903_2025_6433_MOESM7_ESM.docx]

**Results of the subset analyses**

**Effect of cementation**

**Cemented marginal gap:**

The network (Suppl. Fig.1/A) included 14 in vitro studies, 11 two-arm studies and 3 multi-arm studies. The total number of examined ceramic restorations in the network was 511. SUCRA values (Suppl. Fig.1/B) indicated vertical preparation is likely to have the smallest marginal gap (SUCRA: 80.42 %), followed by rounded shoulder- (SUCRA: 50.38%), chamfer- (SUCRA: 36.68 %), and shoulder preparation (SUCRA: 32.5 %). League heat plot for the marginal gap (Suppl. Fig.1/C) represents the pairwise comparisons of different preparation techniques. When comparing the vertical preparation to the rounded shoulder- (MD: 11.51 µm CrI: -20.05, 42.30), chamfer- (MD: 15.47 µm CrI: -18.19, 47.61), and shoulder preparation technique (MD: 18.90 µm CrI: -30.18, 66.52), the vertical preparation was favored. No statistically significant differences were detected between the different preparation designs. The consistency analysis (Supplementary material 6. Figure 4.) showed that the comparisons in the network are consistent.


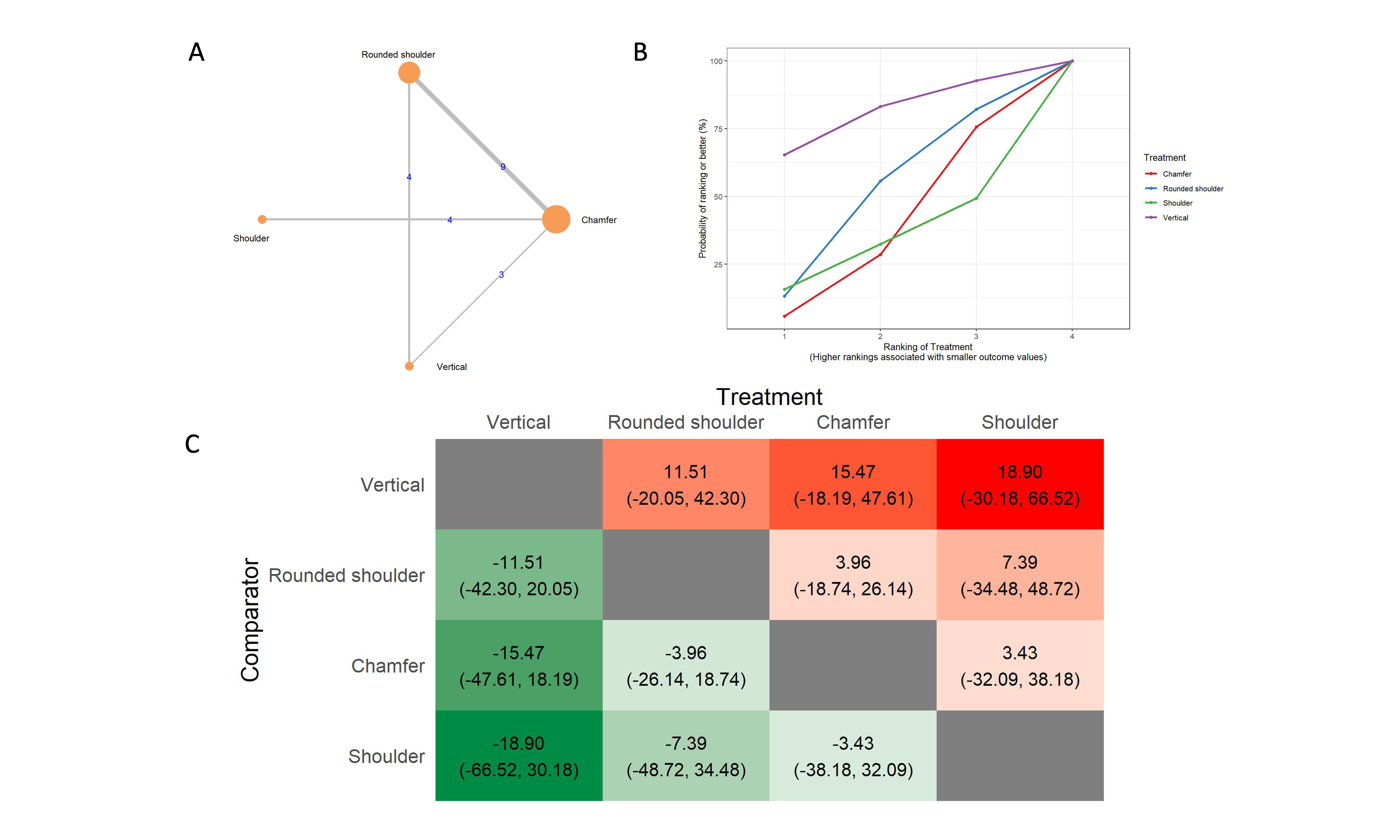


**Supplementary material 7. Figure 1. Cemented marginal gap:** A, Network geometry of the eligible comparisons of cemented marginal gap in case of rounded shoulder, shoulder, chamfer and vertical edge preparation designs. B, Surface under the cumulative ranking curves (SUCRA%) values of maginal gap values. C, The league heat plot shows the mean difference and 95% credible interval for all possible treatment pairs in µm.

**Not cemented marginal gap:**

The network (Suppl. Fig.2 /A) included 17 in vitro studies, 16 two-arm studies and 1 multi-arm study. The total number of examined ceramic restorations in the network was 741. SUCRA values (Suppl. Fig.2 /B) indicated that vertical preparation is likely to have the smallest marginal gap (SUCRA: 91.92%) followed by chamfer- (SUCRA: 41.27 %), rounded shoulder - (SUCRA: 34.58 %), and shoulder preparation (SUCRA: 32.25 %). League heat plot for the marginal gap (Suppl. Fig.2 /C) represents the pairwise comparisons of different preparation techniques. When comparing the vertical preparation to the chamfer- (MD: 18.73 µm CrI: -9.45, 45.63), rounded shoulder - (MD: 19.75 µm CrI: -8.63, 46.36), and shoulder preparation technique (MD: 20.16 µm CrI: -8.96, 48.19), the vertical preparation was favored. No statistically significant differences were detected between the different preparation designs. The consistency analysis (Supplementary material 6. Figure 5.*)* showed that the comparisons in the network are consistent.


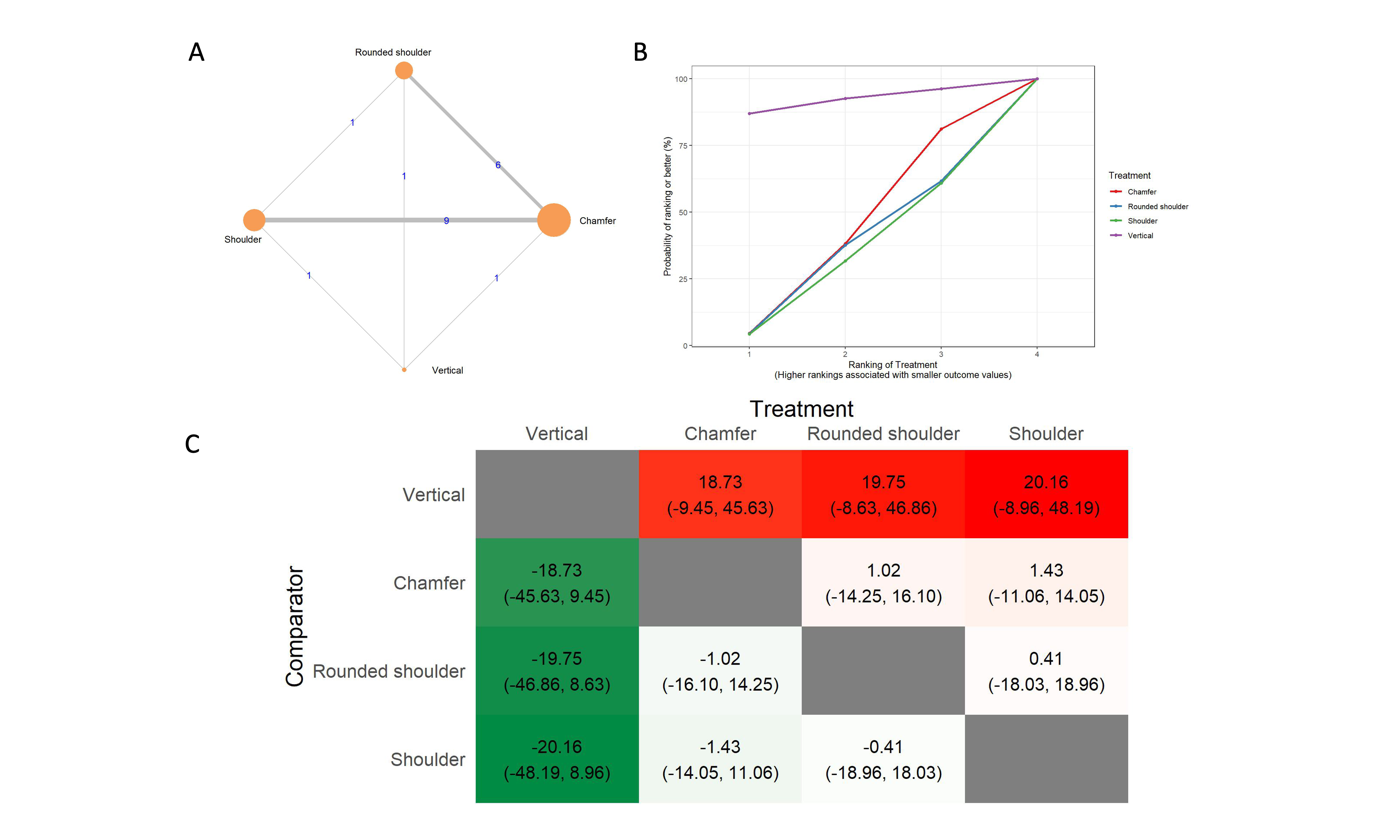


**Supplementary material 7. Figure 2. Not cemented marginal gap** A, Network geometry of the eligible comparisons of not cemented marginal gap in case of rounded shoulder, shoulder, chamfer and vertical edge preparation designs. B, Surface under the cumulative ranking curves (SUCRA%) values of maginal gap values. C, The league heat plot shows the mean difference and 95% credible interval for all possible treatment pairs.

**Cemented absolute marginal discrepancy:**

The network (Suppl. Fig.3/A) included 7 in vitro studies, 5 two-arm studies and 2 multi-arm studies. The total number of examined ceramic restorations in the network was 226. SUCRA values (Suppl. Fig.3/B) indicated that rounded shoulders are likely to have the smallest absolute marginal discrepancy (SUCRA: 81.37%), followed by vertical - (SUCRA: 48.72%), shoulder- (SUCRA: 46.25%), and chamfer- (SUCRA: 23.65 %). League heat plot for the absolute marginal discrepancy (Suppl. Fig.3/C) represents the pairwise comparisons of different preparation techniques. When comparing the rounded shoulder to the vertical- (MD: 17.94 µm CrI: -34.34, 74.37), shoulder- (MD: 19.69 µm CrI: -55.11, 94.29), chamfer preparation technique (MD: 30.08 µm CrI: -6.30, 68.21), the rounded shoulder was favored. The mean differences were not statistically significant. The consistency analysis (Supplementary material 6. Figure 6.) showed that the comparisons in the network are consistent.


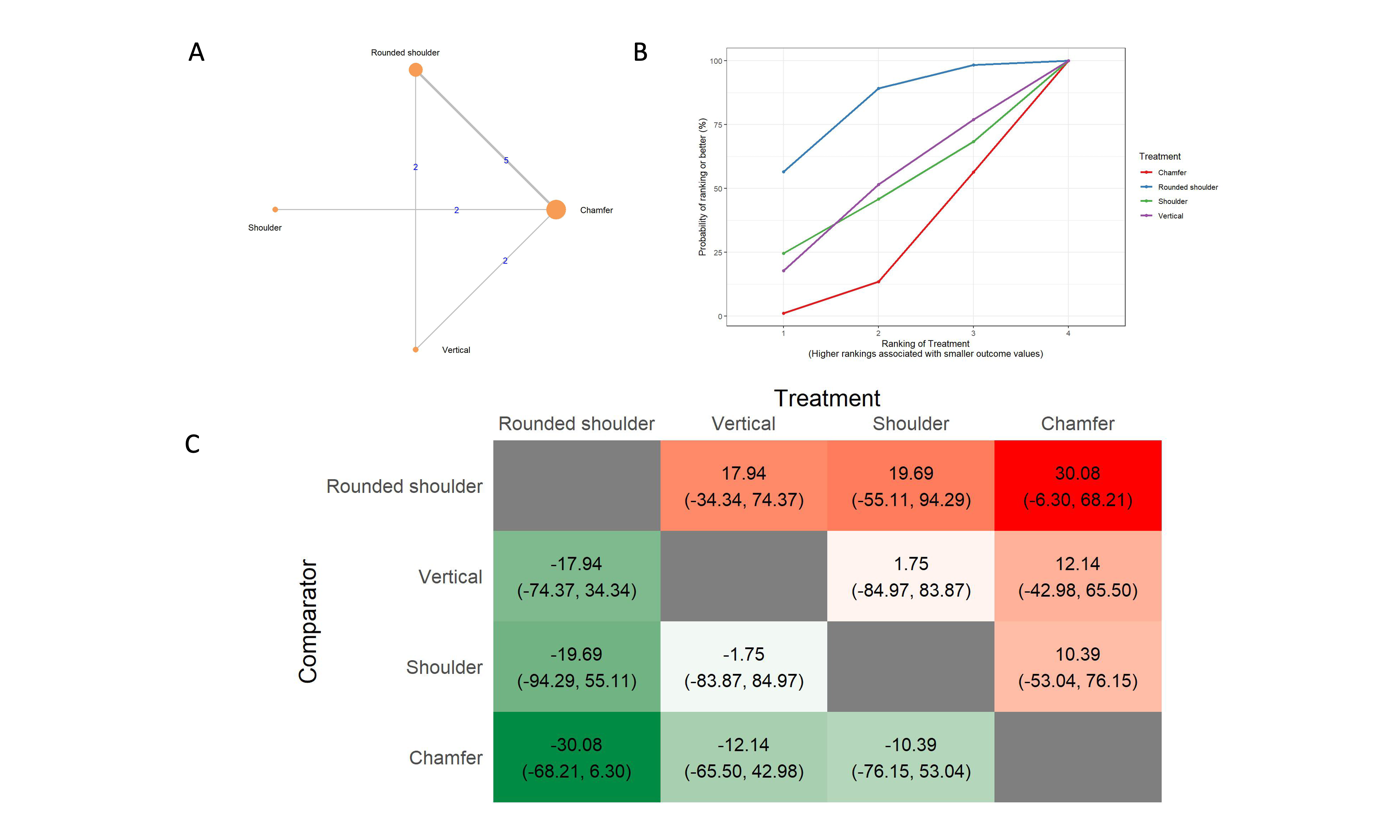


**Supplementary material 7. Figure 3. Cemented absolute marginal discrepancy** A, Network geometry of the eligible comparisons of cemeneted absolute marginal discrepancy in case of rounded shoulder, shoulder, chamfer and vertical edge preparation designs. B, Surface under the cumulative ranking curves (SUCRA%) values of maginal gap values. C, The league heat plot shows the mean difference and 95% credible interval for all possible treatment pairs.

**Not cemented absolute marginal discrepancy:**

The network (Suppl. Fig. 4/A) included 3 in vitro, two-arm studies. The total number of examined ceramic restorations in the network was 140. SUCRA values (Suppl. Fig.4/B) indicated that rounded shoulders are likely to have smaller absolute marginal discrepancy (SUCRA: 92.29%) than chamfer preparation (SUCRA: 7.71 %). League heat plot for the absolute marginal discrepancy (Suppl. Fig.4/C) represents the pairwise comparisons of rounded shoulder and chamfer preparation techniques. When comparing the rounded shoulder to the chamfer (MD: 9.85 µm CrI: -4.69, 23.65), the rounded shoulder was favored. The mean differences were not clinically relevant and not statistically significant. The consistency analysis (Supplementary material 6. Figure 7.) showed that the comparisons in the network are consistent.


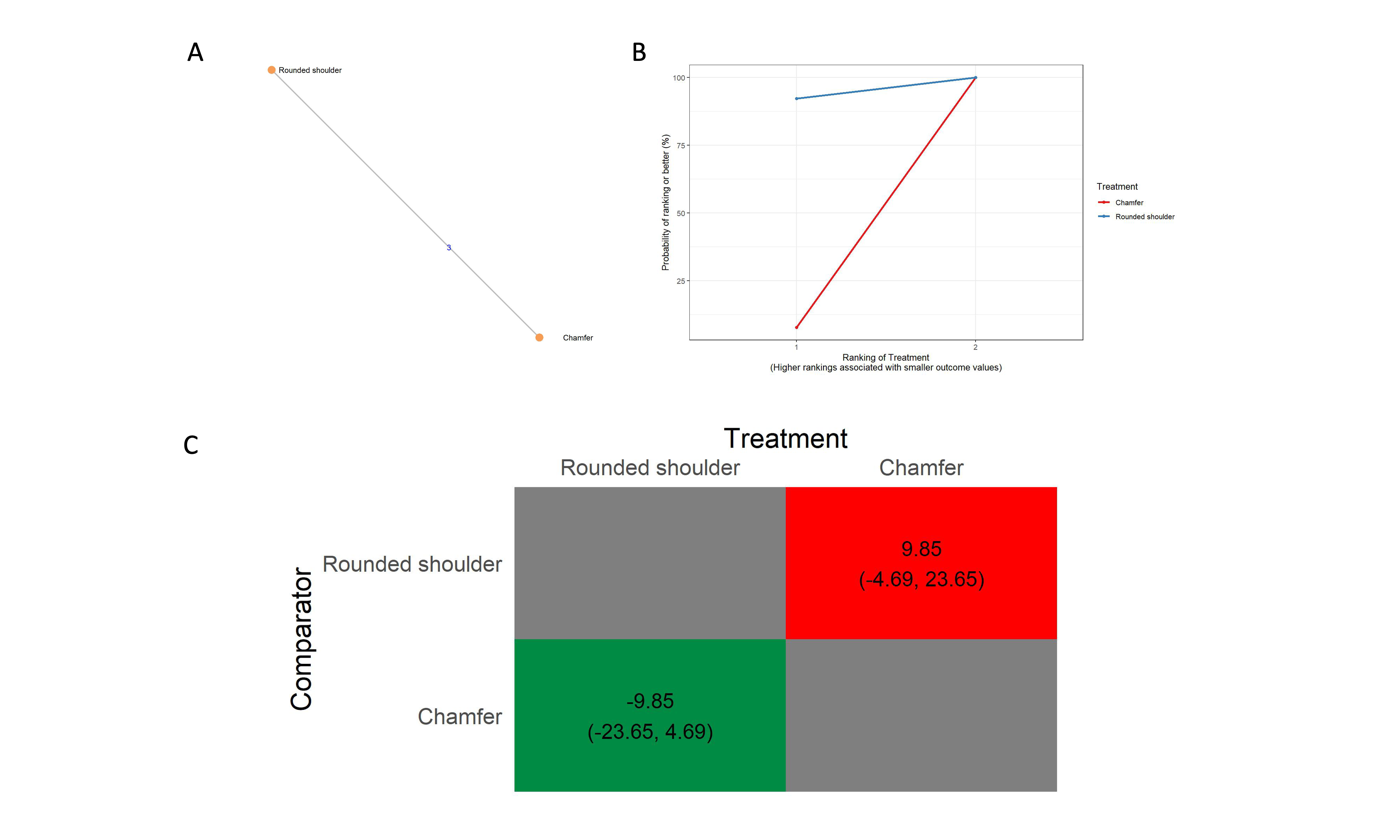


**Supplementary material 7. Figure 4. Not cemented absolute marginal discrepancy** A, Network geometry of the eligible comparisons of not cemented absolute marginal discrepancy in case of rounded shoulder and chamfer. B, Surface under the cumulative ranking curves (SUCRA%) values of absolute marginal discrepancy values. C, The league heat plot shows the mean difference and 95% credible interval for the treatment pairs.

**Cemented internal gap:**

The network (Suppl. Fig.5/A) included 8 in vitro studies, 7 two-arm studies and 1 multi-arm study. The total number of examined ceramic restorations in the network was 222. SUCRA values (Suppl. Fig.5/B) indicated that vertical preparation designs are likely to have the smallest internal gap values (SUCRA: 66.35%) followed by shoulder- (SUCRA: 61.49 %) chamfer- (SUCRA: 60.11 %) and the rounded shoulder preparation (SUCRA: 12.06 %). League heat plot for the cemented internal gap (Suppl. Fig.5/C) represents the pairwise comparisons of different preparation techniques. When comparing the vertical to the shoulder- (MD: 1.00 µm CrI:-111.59, 114.40), and the chamfer-(MD: 6.27 µm CrI:-55.87, 69.94), and rounded shoulder preparation techniques (MD: 38.15 µm CrI: -30.30, 106.14), the vertical preparation was favored. The mean differences were not not statistically significant. The consistency analysis (Supplementary material 6. Figure 8.) showed that the comparisons in the network are consistent.


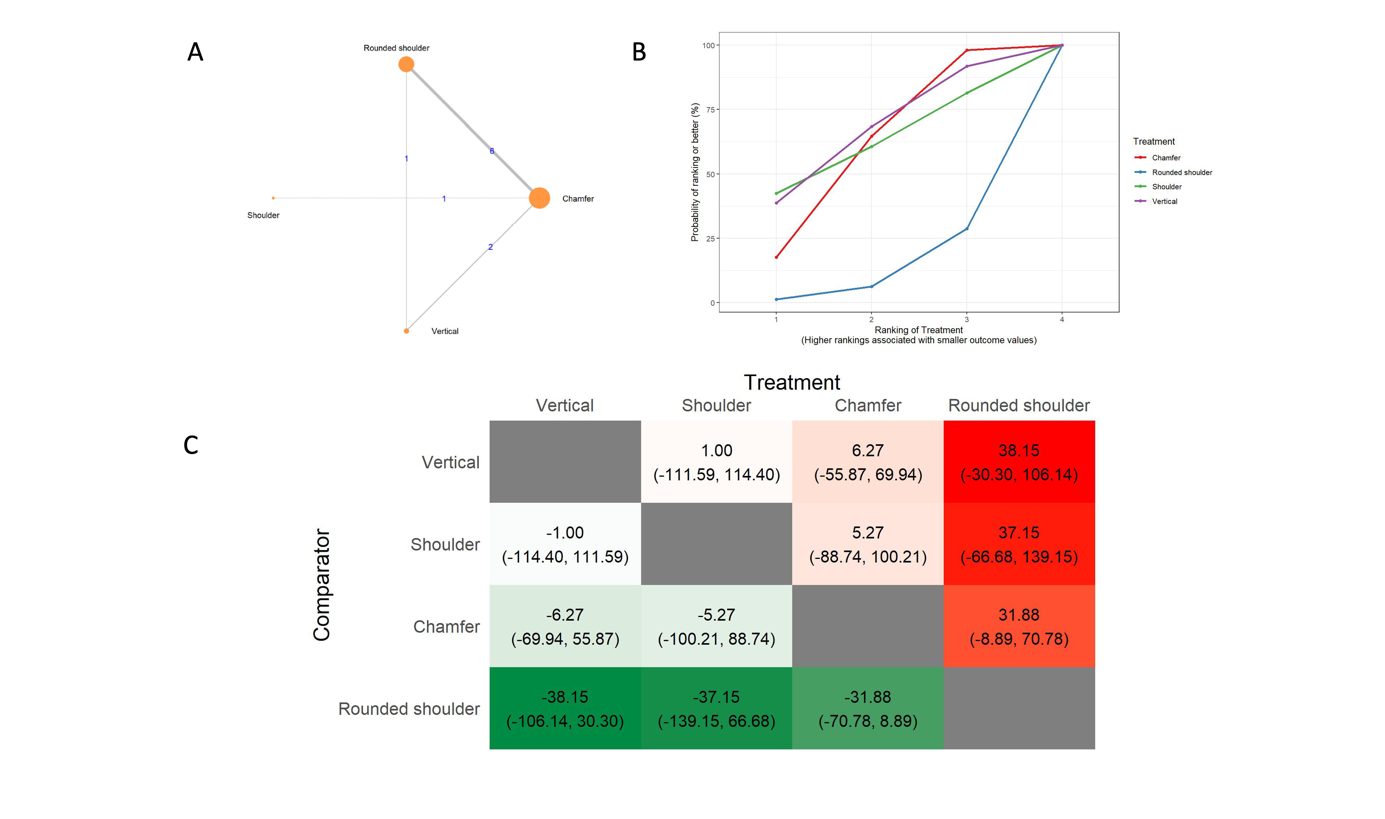


**Supplementary material 7. Figure 5. Cemented internal gap:** A, Network geometry of the eligible comparisons of cemented internal gap in case of rounded shoulder, shoulder, chamfer and vertical edge preparation designs. B, Surface under the cumulative ranking curves (SUCRA%) values of internal gap values. C, The league heat diagram shows the mean difference and 95% credible interval for all possible treatment pairs.

**Not cemented internal gap:**

The network (Suppl. Fig.6/A) included 5 in vitro studies, 4 two-arm studies and 1 multi-arm study. The total number of examined ceramic restorations in the network was 200. SUCRA values (Suppl. Fig.6/B) indicated that chamfer preparation designs are likely to have the smallest internal gap values (SUCRA: 79.36 %), followed by vertical- (SUCRA: 53.52 %), rounded shoulder- (SUCRA: 48.5%), and shoulder preparation (SUCRA: 18.63 %). League heat plot for the internal gap (Suppl. Fig.6/C) represents the pairwise comparisons of different preparation techniques. When comparing the chamfer to the vertical preparation- (MD: -11.97µm CrI: -52.78,75.40), rounded shoulder- (MD: 14.97µm CrI: -32.59,61.26), and shoulder preparation designs (MD: 30.21µm CrI: -12.06,72.59) the chamfer preparation was favored. The mean differences were not not statistically significant. The consistency analysis (Supplementary material 6. Figure 9*.*) showed that the comparisons in the network are consistent.


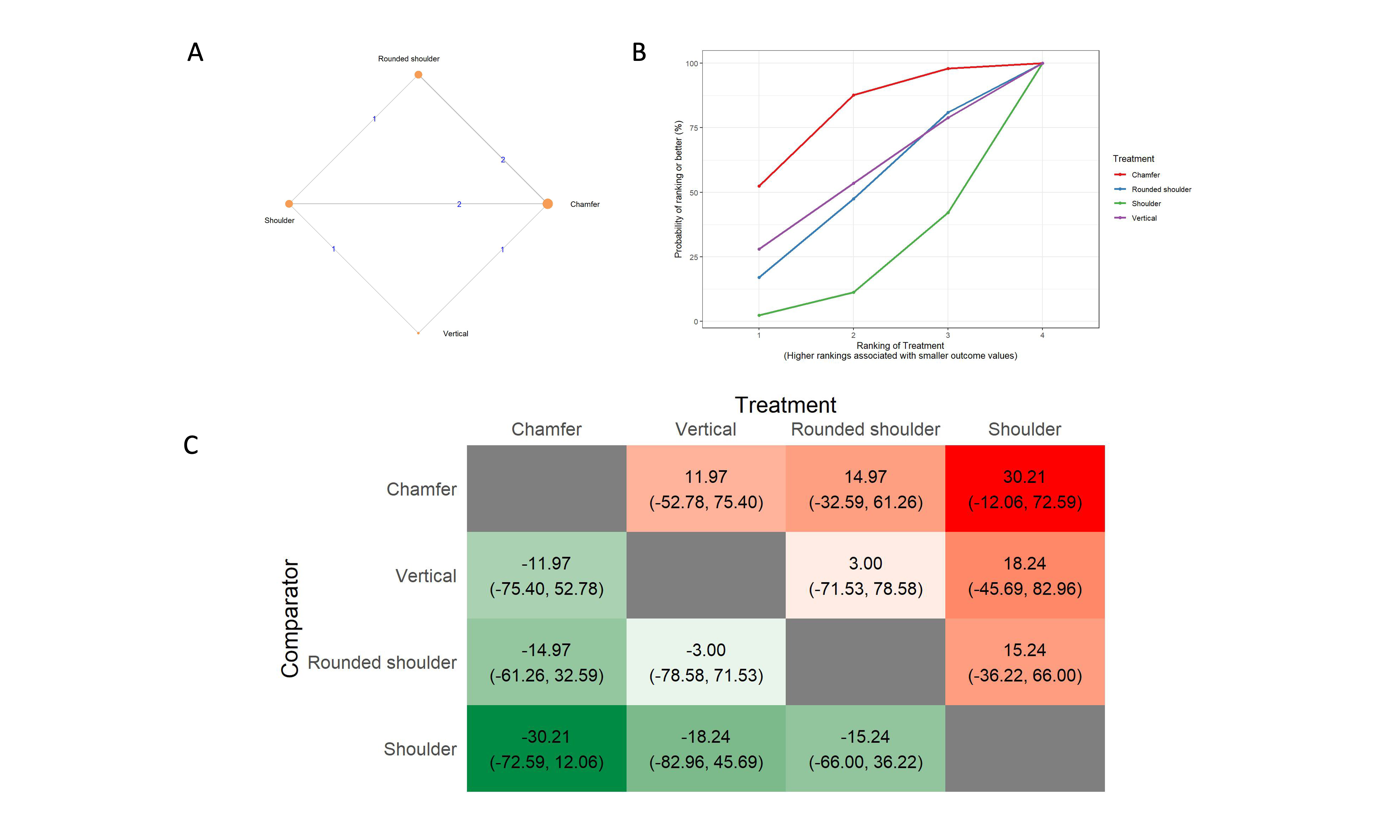


**Supplementary material 7. Figure 6. Not cemented internal gap:** A, Network geometry of the eligible comparisons of not cemented internal gap in case of rounded shoulder, shoulder, chamfer and vertical edge preparation designs. B, Surface under the cumulative ranking curves (SUCRA%) values of internal gap values. C, The league heat diagram shows the mean difference and 95% credible interval for all possible treatment pairs.

**2, Effect of the manufacturing techniques**

**Marginal gap of CAD/CAM all-ceramic system (CAD/CAM: computer-assisted design/computer-assisted manufacturing**):

The network (Suppl. Fig.7/A) included 23 in vitro studies, 20 two-arm studies and 3 multi-arm studies. The total number of examined ceramic restorations in the network was 858. SUCRA values (Suppl. Fig.7/B) indicated that rounded shoulders are likely to have the smallest marginal gap (SUCRA: 63.44 %), followed by vertical- (SUCRA: 54.24 %), chamfer- (SUCRA: 42.44%), and shoulder preparation (SUCRA: 39.88%). League heat plot for the marginal gap (Suppl. Fig.7/C) represents the pairwise comparisons of different preparation techniques. When comparing the rounded shoulder to the vertical- (MD: 1.35 µm CrI: -21.58, 25.05), chamfer- (MD: 3.39 µm CrI: -10.45, 16.92), and shoulder preparation (MD: 4.26 µm CrI: -15.6, 23.84) the rounded shoulder was favored. No statistically significant differences were detected between the different preparation designs. The consistency analysis (Supplementary material 6. Figure 10.) showed that the comparisons in the network are consistent.
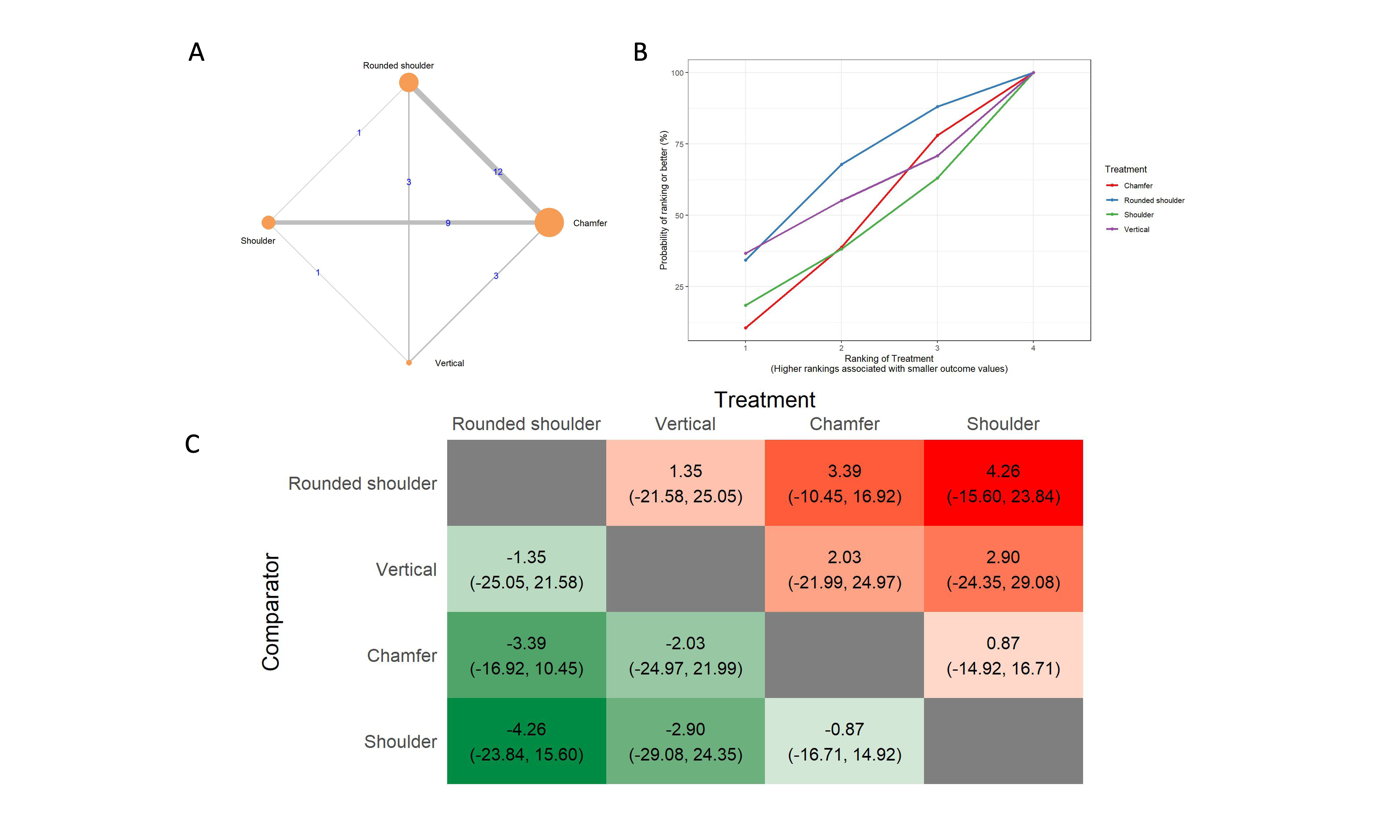


**Supplementary material 7. Figure 7. Marginal gap of CAD/CAM all-ceramic system (CAD/CAM: computer-assisted design/computer-assisted manufacturing**): A, Network geometry of the eligible comparisons of marginal gap in case of rounded shoulder, shoulder, chamfer and vertical edge preparation designs. B, Surface under the cumulative ranking curves (SUCRA%) values of maginal gap values. C, The league heat diagram shows the mean difference and 95% credible interval for all possible treatment pairs.

**Marginal gap of conventional all-ceramic systems:**

The network (Suppl. Fig.8/A) included 9 in vitro studies, 8 two-arm studies and 1 multi-arm study. The total number of examined ceramic restorations in the network was 374. SUCRA values (Suppl. Fig.8/B) indicated that vertical preparations are likely to have the smallest marginal gap (SUCRA: 97.46 %), followed by rounded shoulder- (SUCRA: 58.12 %), chamfer- (SUCRA: 36.76 %), and shoulder preparation (SUCRA: 7.65 %). League heat plot for the marginal gap (Suppl. Fig.8/C) represents the pairwise comparisons of different preparation techniques. When comparing the vertical preparation to the rounded shoulder- (MD: 31.06 µm CrI: -5.40, 67.65), chamfer- (MD: 38.06 µm CrI:1.72,75.00), and shoulder preparation technique (MD: 49.11 µm CrI: 7.01,91.97),the vertical preparation was favored.. There was a statistically significant difference between the vertical- and the chamfer preparation techniques and between the vertical- and the shoulder preparation. The vertical preparation was favored in both cases. The consistency analysis (Supplementary material 6. Figure 11.) showed that the comparisons in the network are consistent.


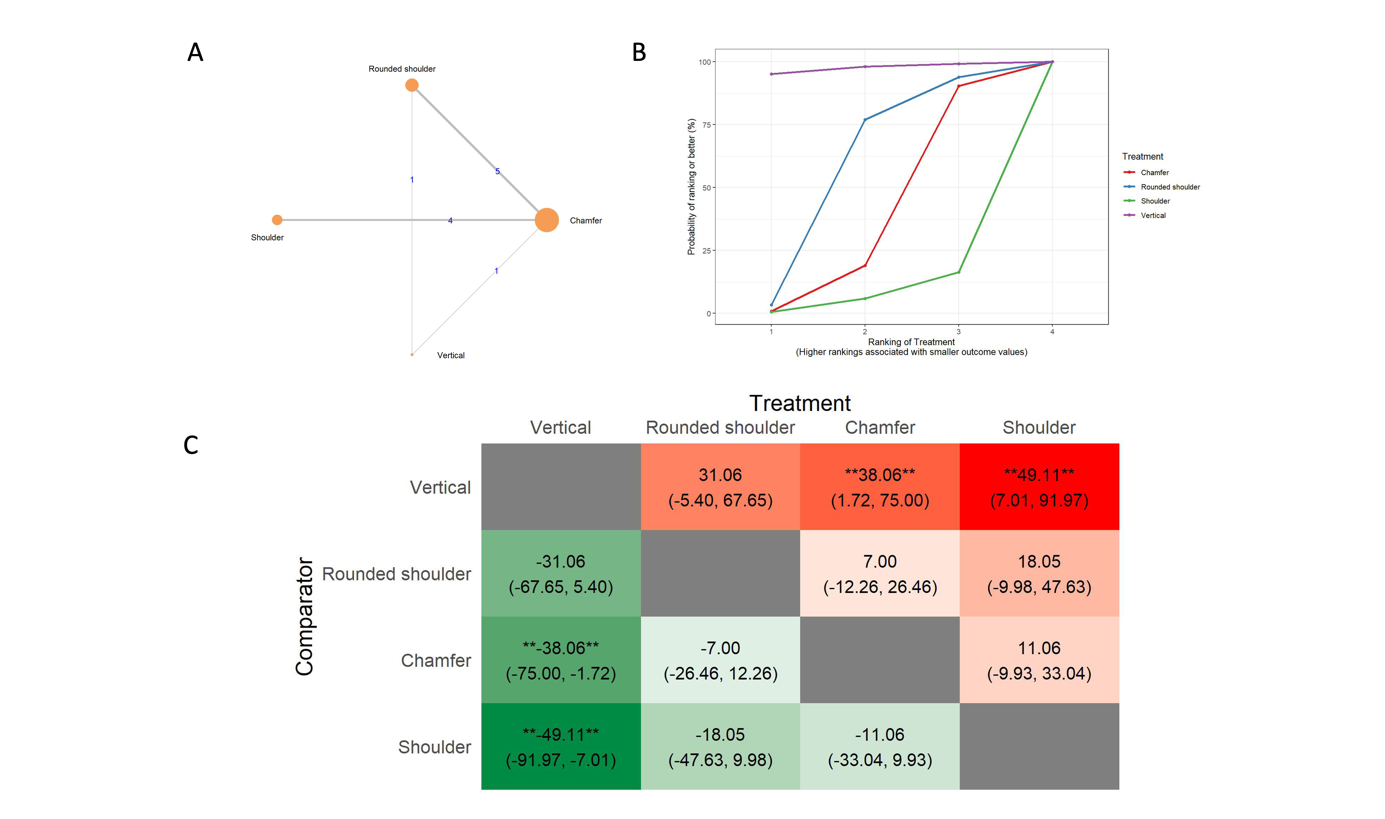


**Supplementary material 7. Figure 8. Marginal gap of conventional all-ceramic system**: A, Network geometry of the eligible comparisons of marginal gap of conventional all-ceramic systems in case of rounded shoulder, shoulder, chamfer and vertical edge preparation designs. B, Surface under the cumulative ranking curves (SUCRA%) values of maginal gap values. C, The league heat diagram shows the mean difference and 95% credible interval for all possible treatment pairs.

**Absolute marginal discrepancy of CAD/CAM all-ceramic system (CAD/CAM: computer-assisted design/computer-assisted manufacturing**):

The network (Suppl. Fig. 9/A) included 7 in vitro studies, 6 two-arm studies and 1 multi-arm study. The total number of examined ceramic restorations in the network was 282. SUCRA values (Suppl. Fig. 9/B) indicated that rounded shoulders are likely to have the smallest absolute marginal discrepancy (SUCRA: 91.29 %), followed by shoulder- (SUCRA: 62.14 %), chamfer- (SUCRA: 42.06 %), and vertical preparation design (SUCRA: 4.52 %). League heat plot for the absolute marginal discrepancy (Suppl. Fig.9/C) represents the pairwise comparisons of different preparation techniques. When comparing the rounded shoulder to the shoulder- (MD: 15.35 µm CrI: -42.87,78.21) ,-chamfer preparation (MD: 25.52 µm CrI: 7.61,50.39), and vertical- (MD: 60.02 µm CrI: 15.56,106.31) the rounded shoulder was favored. If we compare the rounded shoulder with the chamfer, and the rounded shoulder with the vertical preparation, the mean differences were statistically significant, favoring rounded shoulder*.* The consistency analysis (Supplementary material 6. Figure 12.) showed that the comparisons in the network are consistent.


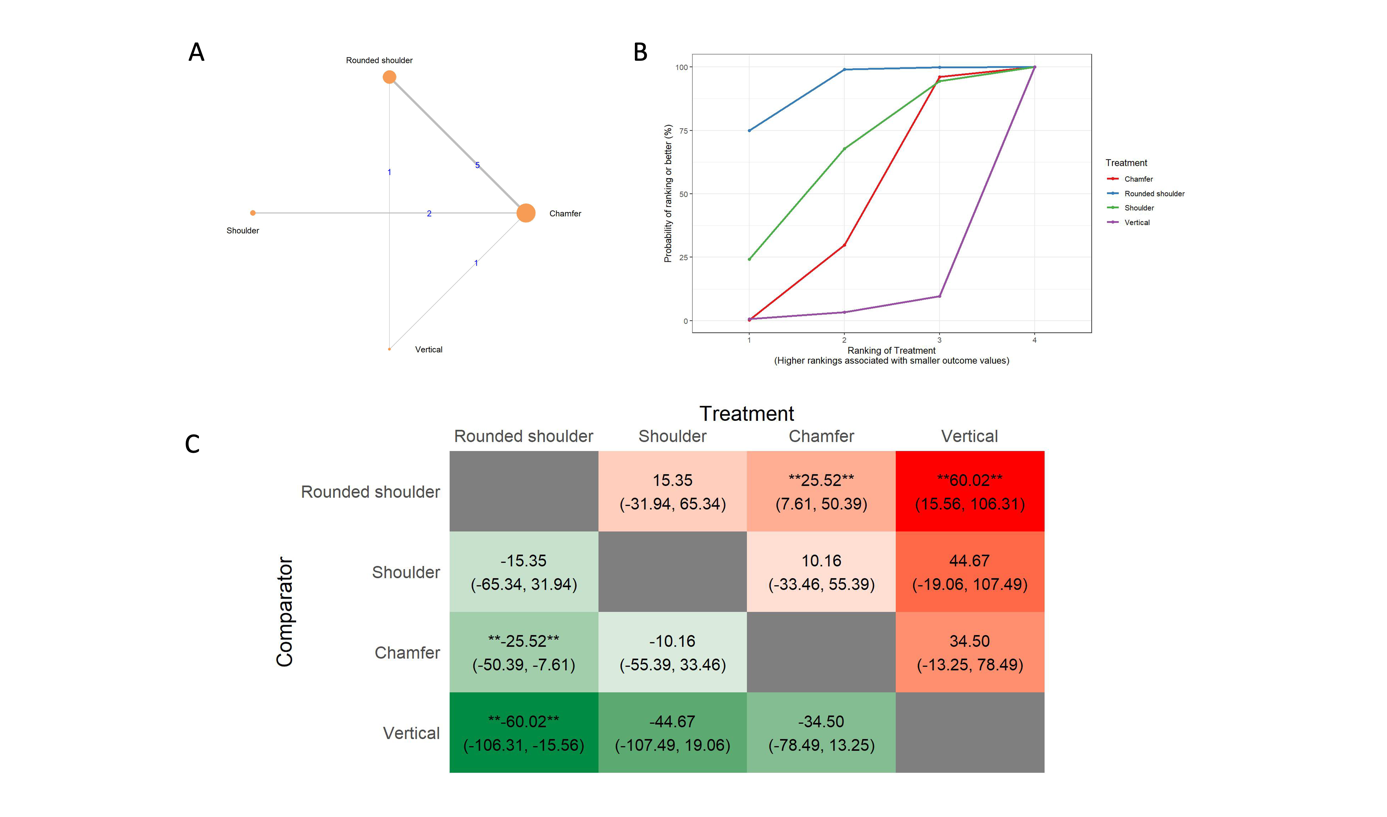


**Supplementary material 7. Figure 9. Absolute marginal discrepancy of CAD/CAM all-ceramic system (CAD/CAM: computer-assisted design/computer-assisted manufacturing**): A, Network geometry of the eligible comparisons of absolute marginal discrepancy of CAD/CAM all-ceramic system in case of rounded shoulder, shoulder, chamfer and vertical edge preparation designs. B, Surface under the cumulative ranking curves (SUCRA%) values of absolute marginal discrepancy values. C, The league heat diagram shows the mean difference and 95% credible interval for all possible treatment pairs.

**Absolute marginal discrepancy of conventional all-ceramic system:**

The network (Suppl. Fig. 10/A) included 3 in vitro studies, 2 two-arm studiesand 1 multi-arm study. The total number of examined ceramic restorations in the network was 84. SUCRA values (Suppl. Fig. 10/B) indicated that vertical preparation is likely to have the smallest absolute marginal discrepancy (SUCRA: 93.86 %), followed by rounded shoulder- (SUCRA: 47.85%), chamfer- (SUCRA: 33.97%) and shoulder preparation technique (SUCRA: 24.31%). League heat plot for the absolute marginal discrepancy (Suppl. Fig.10/C) represents the pairwise comparisons of different preparation techniques. When comparing the vertical tot he rounded shoulder (MD: 31.95µm CrI: -14.69, 77.79), chamfer- (MD: 37.29 µm CrI: -9.14,83.49), and shoulder preparation techniques (MD: 44.17µm CrI: -24.92, 113.53), the vertical preparation was favored. The mean differences were not statistically significant. The consistency analysis (Supplementary material 6. Figure 13.) showed that the comparisons in the network are consistent.


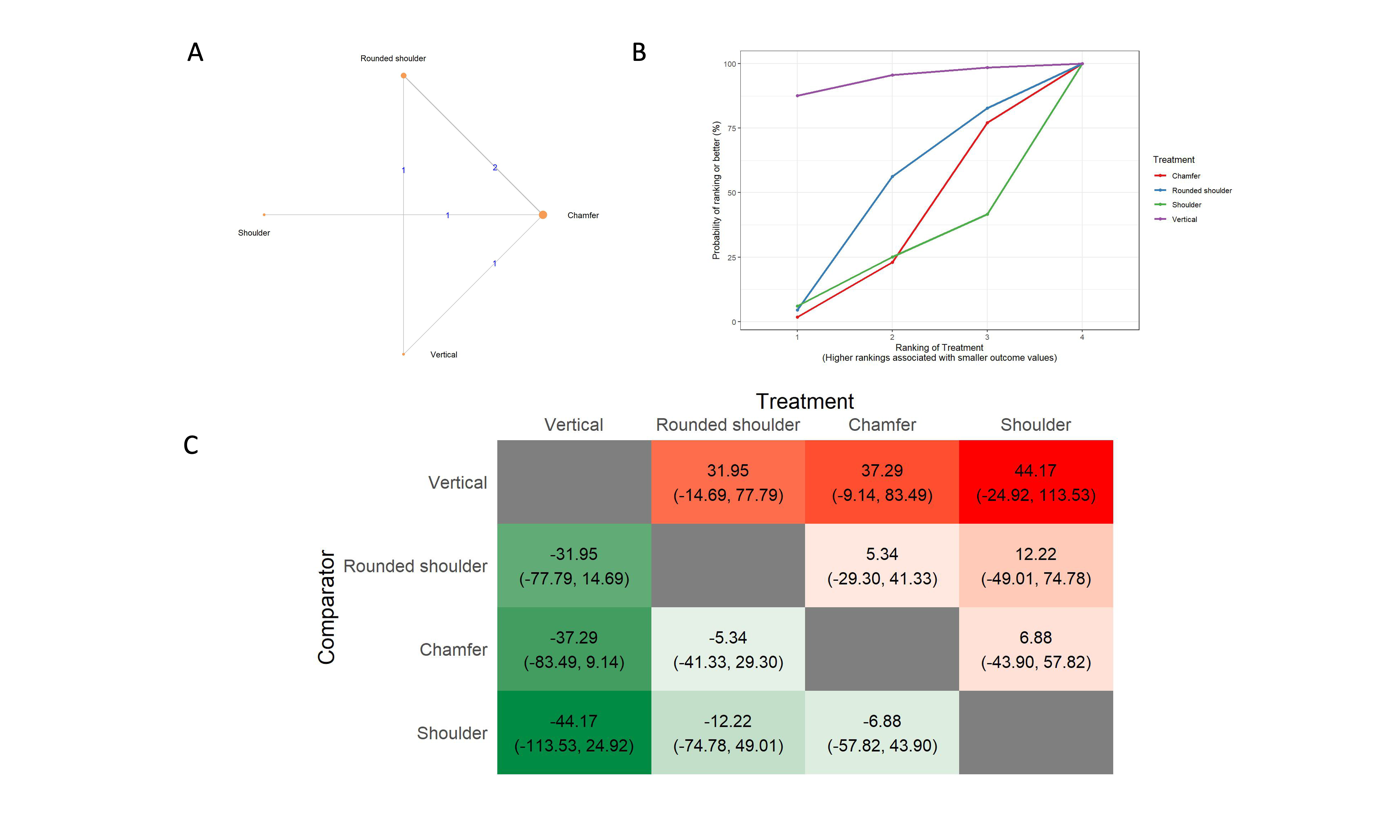


**Supplementary material 7. Figure 10. Absolute marginal discrepancy of conventional all-ceramic system:** A, Network geometry of the eligible comparisons of absolute marginal discrepancy of conventional all-ceramic system in case of rounded shoulder, shoulder, chamfer and vertical edge preparation designs. B, Surface under the cumulative ranking curves (SUCRA%) values of absolute marginal discrepancy values. C, The league heat diagram shows the mean difference and 95% credible interval for all possible treatment pairs.

**Internal gap of CAD/CAM all-ceramic system (CAD/CAM: computer-assisted design/computer-assisted manufacturing**):

The network (Suppl. Fig.11/A) included 12 in vitro studies, 10 two-arm studies and 2 multi-arm studies. The total number of examined ceramic restorations in the network was 390. SUCRA values (Suppl. Fig.11/B) indicated that chamfer preparation designs are likely to have the smallest internal gap values (SUCRA: 81.58 %), followed by vertical- (SUCRA: 77.35 %), shoulder- (SUCRA: 20.83 %), and the rounded shoulder preparation (SUCRA: 20.23 %). League heat plot for the internal gap (Suppl. Fig.11/C) represents the pairwise comparisons of different preparation techniques. If we compare the chamfer to the vertical preparation (MD: -0.57 µm CrI: -39.22, 39.19), to the shoulder (MD: 32.63 µm CrI: -9.23, 73.9) and the rounded shoulder (MD: 31.39 µm CrI: 4.44, 57.19), the chamfer was favored. The mean difference between the chamfer and the rounded shoulder was statistically significant. The consistency analysis (Supplementary material 6 Figure 14.) showed that the comparisons in the network are consistent.


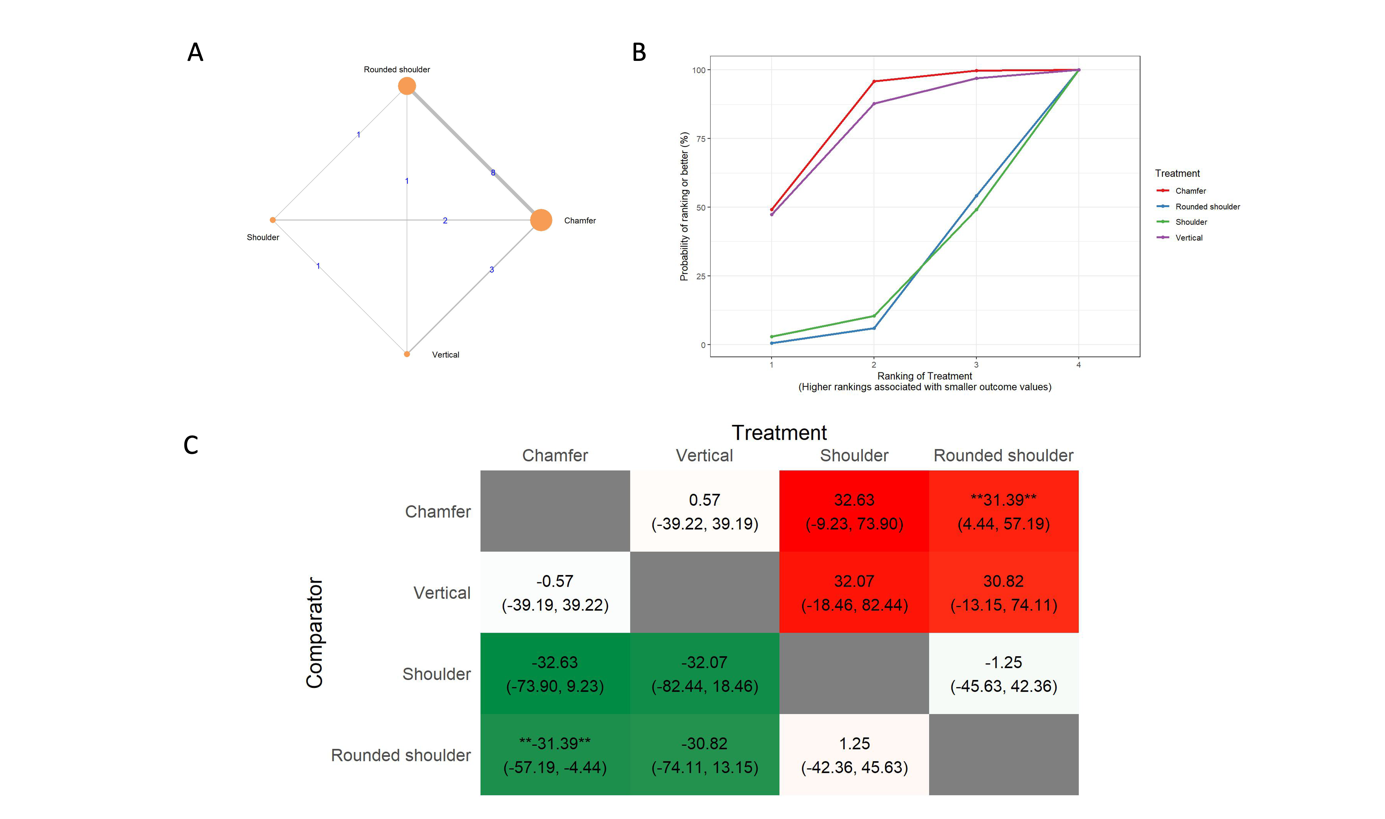


**Supplementary material 7. Figure 11. Internal gap of CAD/CAM all-ceramic system (CAD/CAM: computer-assisted design/computer-assisted manufacturing**): A, Network geometry of the eligible comparisons of internal gap of CAD/CAM all-ceramic system in case of rounded shoulder, shoulder, chamfer and vertical edge preparation designs. B, Surface under the cumulative ranking curves (SUCRA%) values of internal gap values. C, The league heat diagram shows the mean difference and 95% credible interval for all possible treatment pairs.

**Evaluation techniques**

**Marginal gap evaluation using the direct view technique:**

The network (Supplementary material 6. Figure 12./A) included 18 in vitro studies, 17 two-arm studies and 1 multi-arm studies. The total number of examined ceramic restorations in the network was 832. SUCRA values (Supplementary material 6. Figure 12/B) indicated that vertical preparation designs are likely to have the smallest marginal gap values (SUCRA: 99.35 %), followed by rounded shoulder- (SUCRA: 51.07 %), chamfer- (SUCRA: 32.57 %), and shoulder preparation (SUCRA: 17.02 %). League heat plot for the marginal gap (Supplementary material 6. Figure 12/C) represents the pairwise comparisons of different preparation techniques. If we compare the vertical to the rounded shoulder (MD: 29.45 µm CrI: 6.54, 52.91), to the chamfer (MD: 32.96 µm CrI: 8.81, 57.48) and shoulder preparation (MD: 36.38 µm CrI: 9.11, 64.04), the vertical preparation was favored. The mean differences were statistically significant. The consistency analysis (Supplementary material 6 Figure 15.) showed that the comparisons in the network are consistent.

**
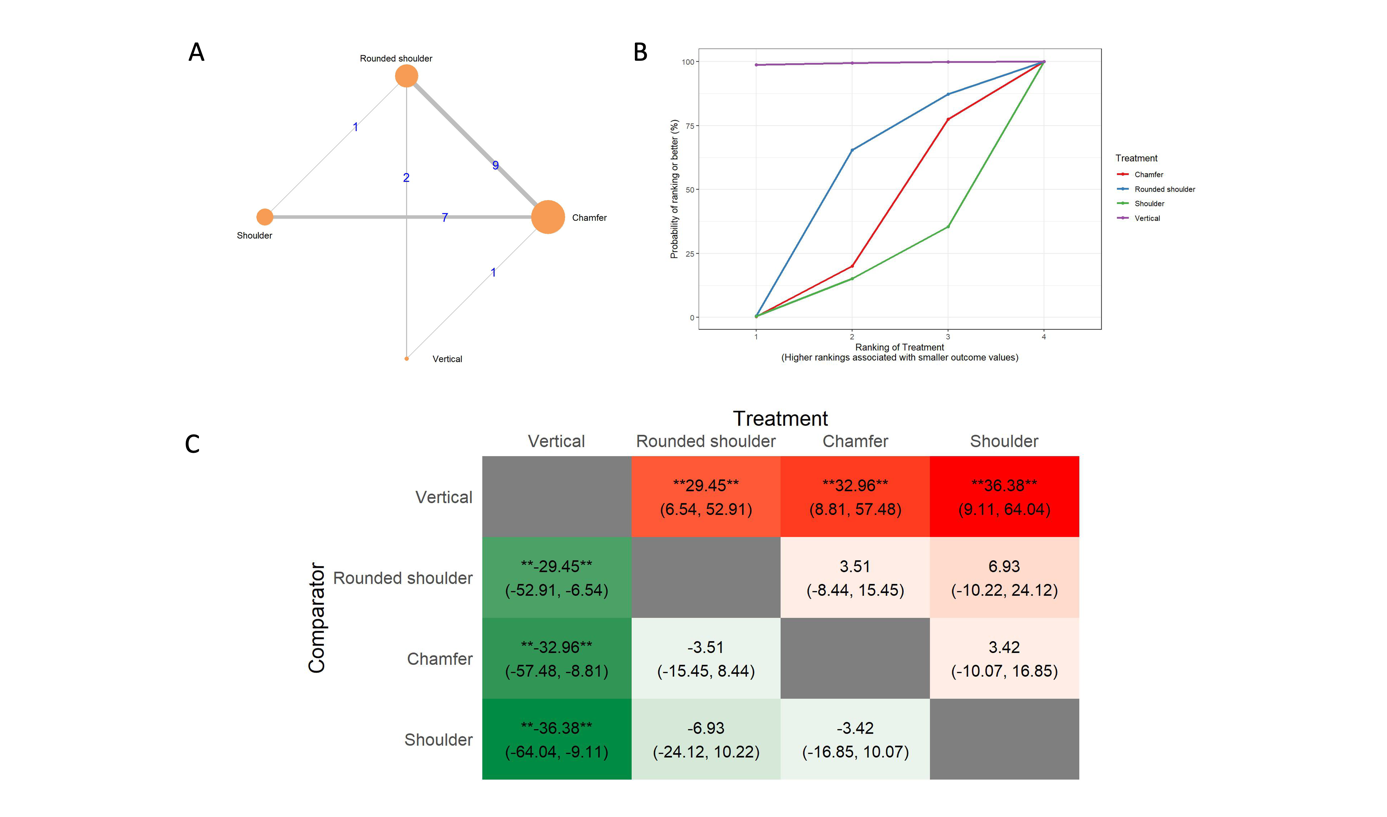
**

**Supplementary material 7. Figure 12. Marginal gap evaluation using the direct view technique:** A, Network geometry of the eligible comparisons of marginal gap evaluation using the direct view technique in case of rounded shoulder, shoulder, chamfer and vertical edge preparation designs. B, Surface under the cumulative ranking curves (SUCRA%) values of marginal gap values. C, The league heat diagram shows the mean difference and 95% credible interval for all possible treatment pairs.

**Marginal gap evaluation using the cross sectioned technique:**

The network (Supplementary material 6. Figure 13/A) included 6 in vitro studies, 4 two-arm studies and 2 multi-arm studies. The total number of examined ceramic restorations in the network was 210. SUCRA values (Supplementary material 6. Figure 13/B) indicated that vertical preparation designs are likely to have the smallest marginal gap values (SUCRA: 72.59 %), followed by shoulder- (SUCRA: 57.86 %), chamfer- (SUCRA: 35.51 %), and the rounded shoulder preparation (SUCRA: 34.03 %). League heat plot for the marginal gap (Supplementary material 6. Figure 13/ C) represents the pairwise comparisons of different preparation techniques. If we compare the vertical to the shoulder (MD: 5.39 µm CrI: -43.90, 54.21), to the chamfer (MD: 14.27 µm CrI: -24.52, 52.48) and the rounded shoulder (MD: 15.47 µm CrI: -27.80, 59.28), the vertical preparation was favored. The consistency analysis (Supplementary material 6 Figure 16.) showed that the comparisons in the network are consistent.

**
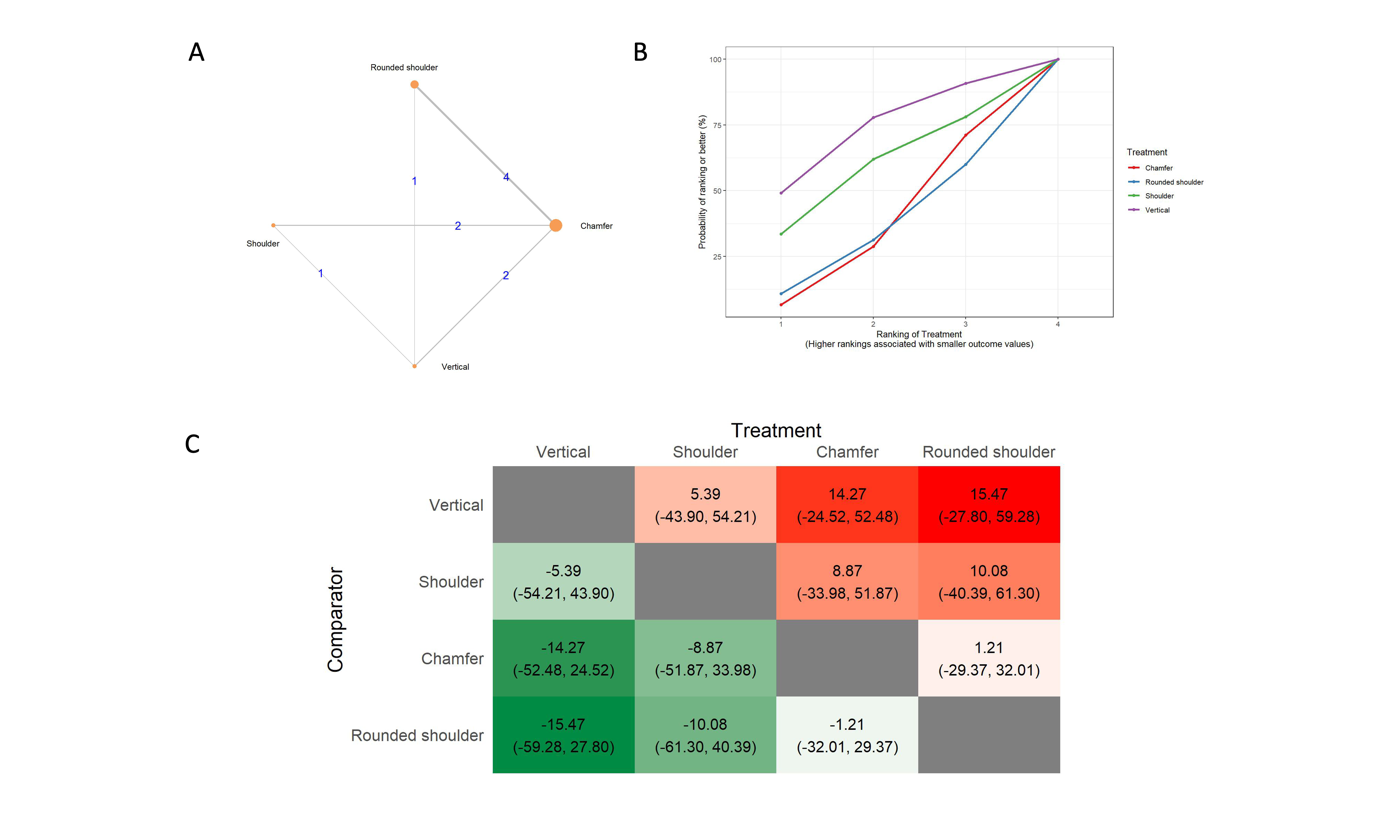
**

**Supplementary material 7. Figure 13. Marginal gap evaluation using the cross sectioned technique:** A, Network geometry of the eligible comparisons of marginal gap evaluation using the cross sectioned technique in case of rounded shoulder, shoulder, chamfer and vertical edge preparation designs. B, Surface under the cumulative ranking curves (SUCRA%) values of marginal gap values. C, The league heat diagram shows the mean difference and 95% credible interval for all possible treatment pairs.

**Marginal gap evaluation using the silicon replica technique:**

The network (Supplementary material 6. Figure 14./A) included 2 in vitro studies, 2 two-arm studies and 0 multi-arm studies. The total number of examined ceramic restorations in the network was 120. SUCRA values (Supplementary material 6. Figure 14/B) indicated that chamfer preparation designs are likely to have smaller marginal gap values (SUCRA: 59.6 %), than shoulder- (SUCRA: 40.4%). League heat plot for the marginal gap (Supplementary material 6. Figure 14./C) represents the pairwise comparisons of different preparation techniques. If we compare the chamfer to the shulder preparation (MD: 0.66 µm CrI: -5.28,6.54), the chamfer was favored. The consistency analysis (Supplementary material 6 Figure 17.) showed that the comparisons in the network are consistent.

**
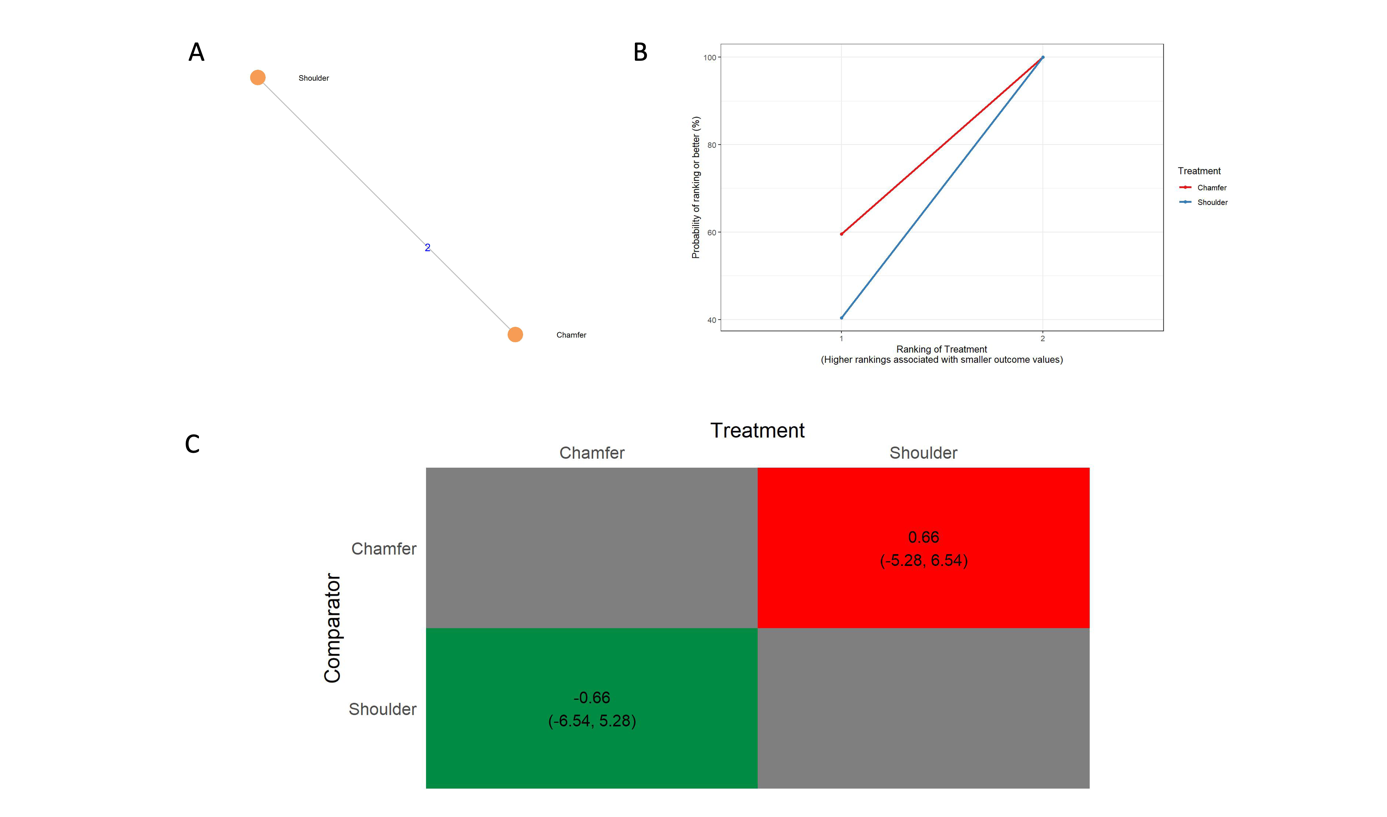
**

**Supplementary material 7. Figure 14. Marginal gap evaluation using the silicon replica technique:** A, Network geometry of the eligible comparisons of marginal gap evaluation using the silicon replica technique in case of shoulder and chamfer preparation designs. B, Surface under the cumulative ranking curves (SUCRA%) values of marginal gap values. C, The league heat diagram shows the mean difference and 95% credible interval for all possible treatment pairs.

**Marginal gap evaluation using the micro-CT technique:**

The network (Supplementary material 6. Figure 15/A) included 2 in vitro studies, 1 two-arm studies and 1 multi-arm studies. The total number of examined ceramic restorations in the network was 70. SUCRA values (Supplementary material 6. Figure 15/B) indicated that rounded shoulder preparation designs are likely to have the smallest marginal gap values (SUCRA: 91.47 %), followed by chamfer- (SUCRA: 53.16 %), and vertical preparation (SUCRA: 5.37 %). League heat plot for the marginal gap (Supplementary material 6. Figure 15/C) represents the pairwise comparisons of different preparation techniques. If we compare the rounded shoulder to the chamfer (MD: 21.29 µm CrI: -33.12, 76.84), and the vertical preparation (MD: 68.81 µm CrI: -7.63, 143.67), the rounded shoulder was favored. The consistency analysis (Supplementary material 6 Figure 18.) showed that the comparisons in the network are consistent.

**
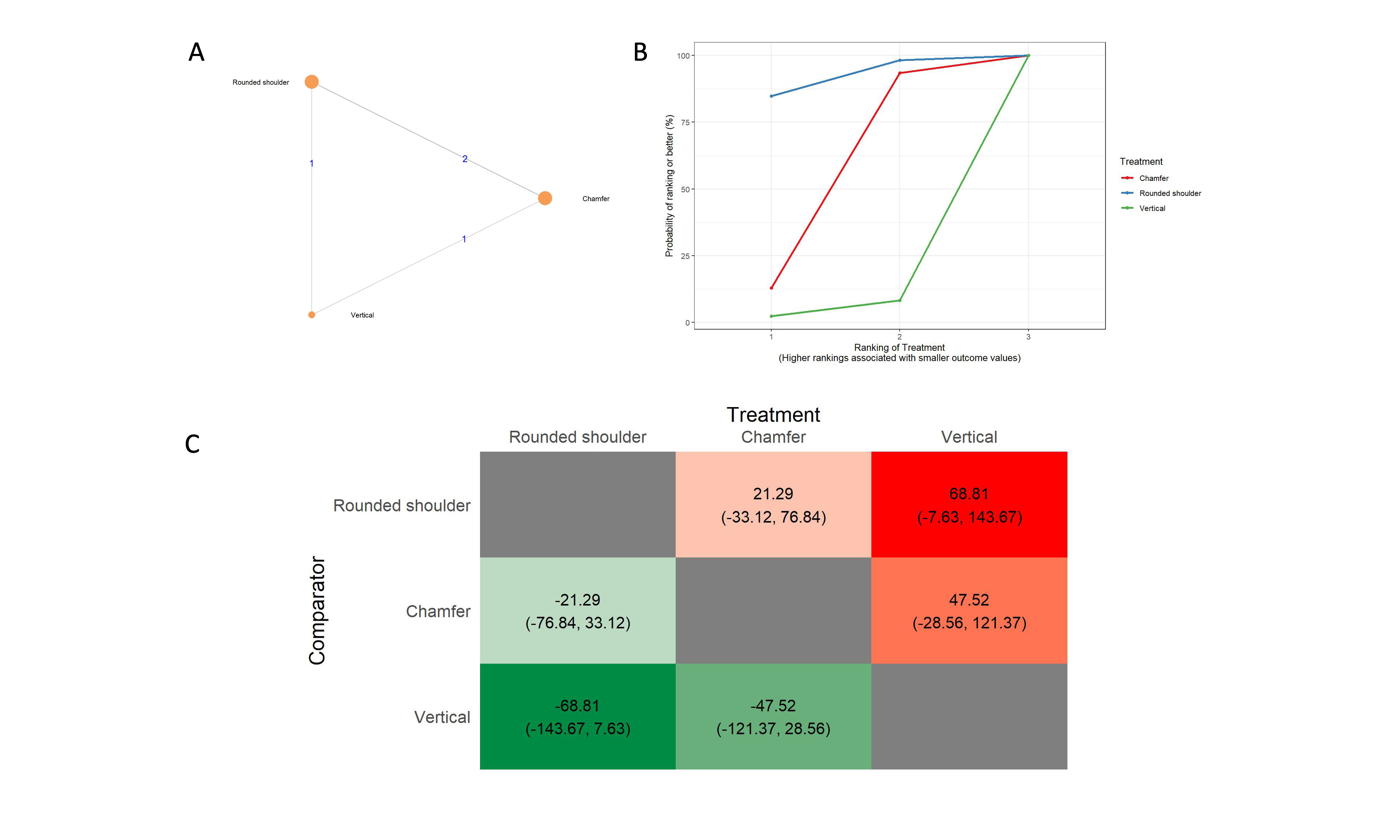
**

**Supplementary material 7. Figure 15. Marginal gap evaluation using the micro-CT technique:** A, Network geometry of the eligible comparisons of marginal gap evaluation using the micro-CT technique in case of rounded shoulder, chamfer and vertical edge preparation designs. B, Surface under the cumulative ranking curves (SUCRA%) values of marginal gap values. C, The league heat diagram shows the mean difference and 95% credible interval for all possible treatment pairs.

**Absolute marginal discrepancy evaluation using the direct view technique:**

The network (Supplementary material 6. Figure 16/A) included 4 in vitro studies, 4 two-arm studies and 0 multi-arm studies. The total number of examined ceramic restorations in the network was 208. SUCRA values (Supplementary material 6. Figure 16/B) indicated that rounded shoulder preparation designs are likely to have the smallest AMD values (SUCRA: 83.3 %), followed by shoulder- (SUCRA: 35.59 %), and chamfer preparation (SUCRA: 31.12 %). League heat plot for the marginal gap (Supplementary material 6. Figure 16/C) represents the pairwise comparisons of different preparation techniques. If we compare the rounded shoulder to the shoulder (MD: 11.77 µm CrI: -24.88, 48.04), and the chamfer preparation (MD: 9.95 µm CrI: -4.69, 23.80), the rounded shoulder was favored. The consistency analysis (Supplementary material 6 Figure 19.) showed that the comparisons in the network are consistent.

**
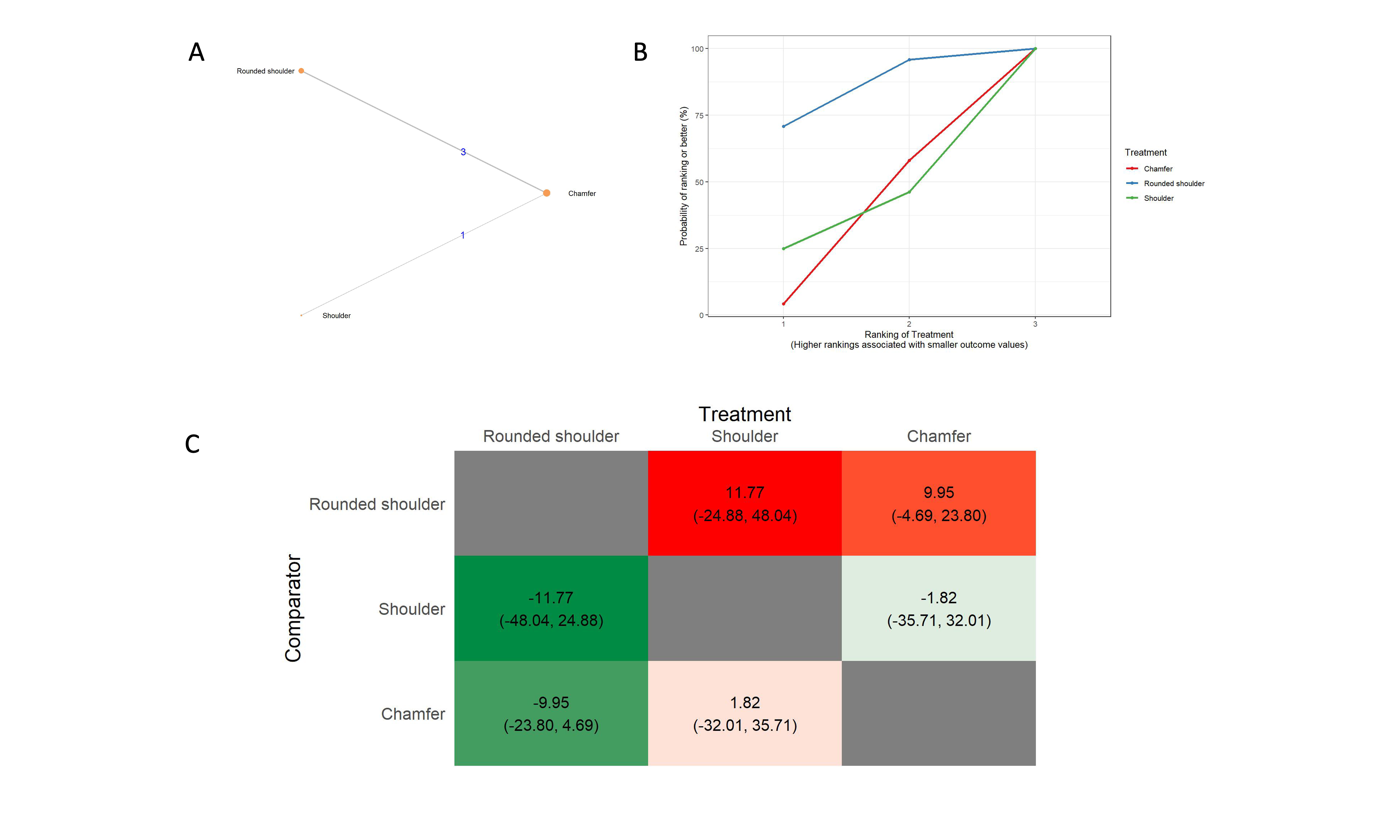
**

**Supplementary material 7. Figure 16. Absolute marginal discrepancy evaluation using the direct view technique:** A, Network geometry of the eligible comparisons of absolute marginal discrepancy evaluation using the direct view technique in case of rounded shoulder, shoulder and chamfer preparation designs. B, Surface under the cumulative ranking curves (SUCRA%) values of marginal gap values. C, The league heat diagram shows the mean difference and 95% credible interval for all possible treatment pairs.

**Absolute marginal discrepancy evaluation using the cross sectioned technique:**

The network (Supplementary material 6. Figure 17/A) included 3 in vitro studies, 2 two-arm studies and 1 multi-arm studies. The total number of examined ceramic restorations in the network was 88. SUCRA values (Supplementary material 6. Figure 17/B) indicated that vertical preparation designs are likely to have the smallest AMD values (SUCRA: 74.36 %), followed by rounded shoulder- (SUCRA: 59.26 %), shoulder (SUCRA: 51.2 %) and chamfer preparation (SUCRA: 15.19 %). League heat plot for the AMD (Supplementary material 6. Figure 17/C) represents the pairwise comparisons of different preparation techniques. If we compare the vertical preparation to the rounded shoulder (MD: 14.57 µm CrI: -75.62, 102.95), the shoulder (MD: 22.91 µm CrI: -118.25, 165.55), and chamfer (MD: 53.74 µm CrI: -34.56, 143.74 )the vertical preparation was favored. The consistency analysis (Supplementary material 6 Figure 20.) showed that the comparisons in the network are consistent.

**
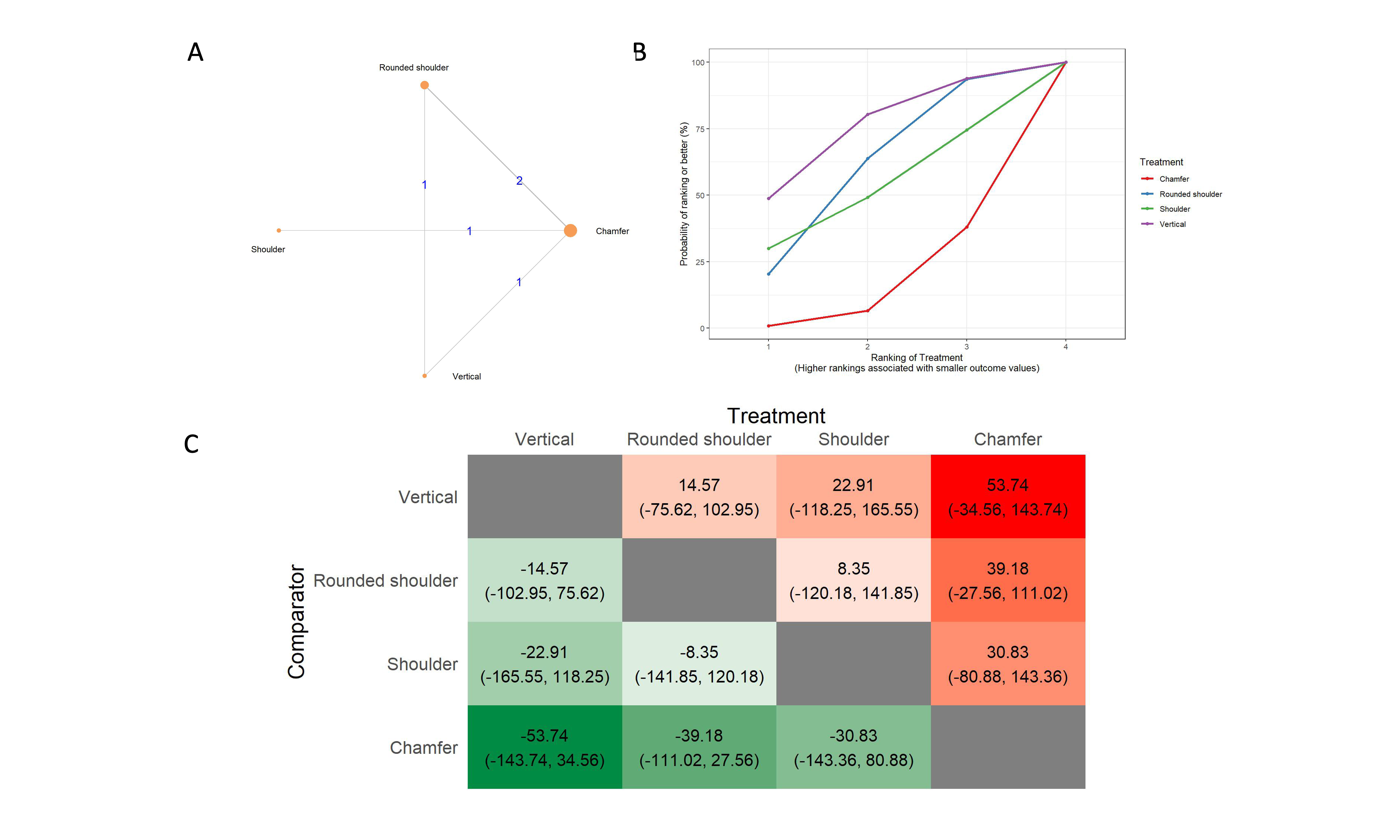
**

**Supplementary material 7. Figure 17. Absolute marginal discrepancy evaluation using the cross sectioned technique:** A, Network geometry of the eligible comparisons of absolute marginal discrepancy evaluation using the cross sectioned technique in case of rounded shoulder, shoulder and chamfer and vertical preparation designs. B, Surface under the cumulative ranking curves (SUCRA%) values of marginal gap values. C, The league heat diagram shows the mean difference and 95% credible interval for all possible treatment pairs.

**Internal gap evaluation using the cross sectioned technique:**

The network (Supplementary material 6. Figure 18/A) included 7 in vitro studies, 6 two-arm studies and 1 multi-arm studies. The total number of examined ceramic restorations in the network was 212. SUCRA values (Supplementary material 6. Figure 18/B) indicated that chamfer preparation designs are likely to have the smallest internal gap values (SUCRA: 80.24 %), followed by vertical (SUCRA: 56.98 %), shoulder (SUCRA: 38.92 %) and rounded shoulder preparation (SUCRA: 23.87 %). League heat plot for the internal gap (Supplementary material 6. Figure 18/C) represents the pairwise comparisons of different preparation techniques. If we compare the chamfer to the vertical (MD: 13.06 µm CrI: -56.99, 82.63), the shoulder (MD: 26.15 µm CrI: -44.25, 96.18), and rounded shoulder (MD: 36.90 µm CrI: -16.95, 89.04) the chamfer was favored. The consistency analysis (Supplementary material 6 Figure 21.) showed that the comparisons in the network are consistent.

**
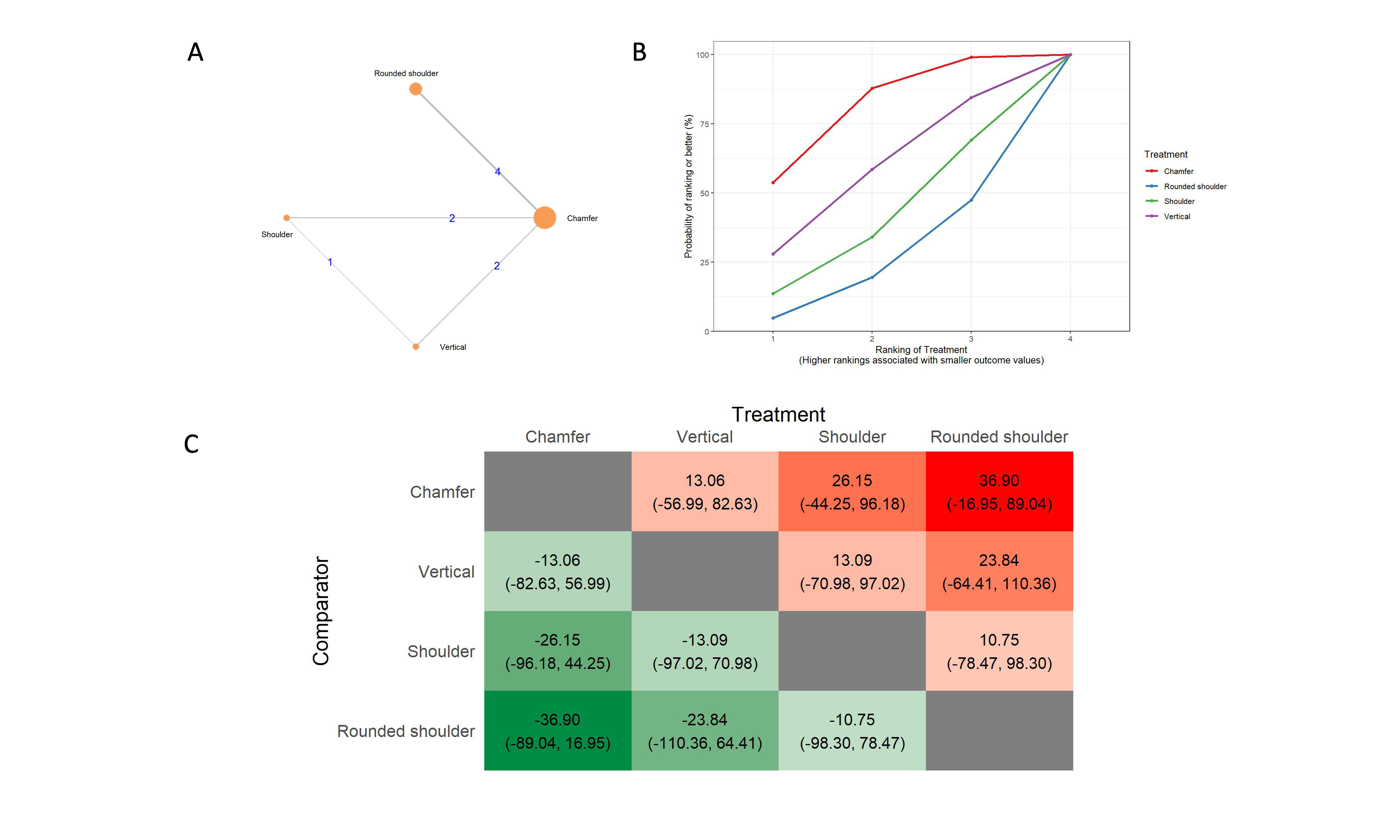
**

**Supplementary material 7. Figure 18. Internal gap evaluation using the cross sectioned technique:** A, Network geometry of the eligible comparisons of internal gap evaluation using the cross sectioned technique in case of rounded shoulder, shoulder and chamfer and vertical preparation designs. B, Surface under the cumulative ranking curves (SUCRA%) values of marginal gap values. C, The league heat diagram shows the mean difference and 95% credible interval for all possible treatment pairs.

**Internal gap evaluation using the silicon replica technique:**

The network (Supplementary material 6. Figure 19/A) included 4 in vitro studies, 4 two-arm studies and 0 multi-arm studies. The total number of examined ceramic restorations in the network was 140. SUCRA values (Supplementary material 6. Figure 19/B) indicated that chamfer preparation designs are likely to have the smallest internal gap values (SUCRA: 82.88 %), followed by rounded shoulder (SUCRA: 47.57 %), shoulder (SUCRA: 19.56 %). League heat plot for the internal gap (Supplementary material 6. Figure 19/C) represents the pairwise comparisons of different preparation techniques. If we compare the chamfer to the rounded shoulder (MD: 9.21 µm CrI: -19.79, 36.25), the shoulder (MD: 16.45 µm CrI: -12.64, 42.02, the chamfer was favored. The consistency analysis (Supplementary material 6 Figure 22.) showed that the comparisons in the network are consistent.

**
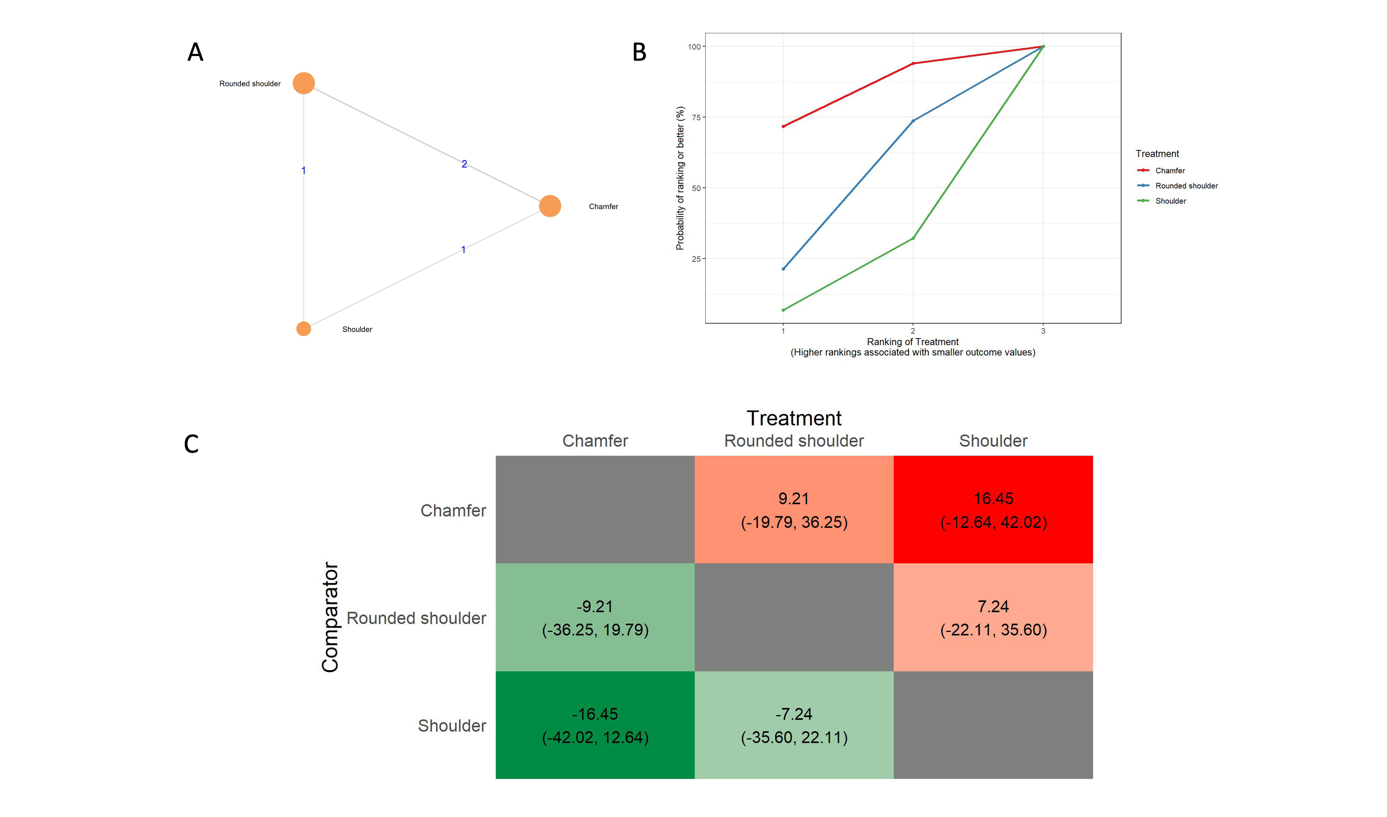
**

**Supplementary material 7. Figure 18. Internal gap evaluation using the silicon replica technique:** A, Network geometry of the eligible comparisons of internal gap evaluation using the silicon replica technique in case of rounded shoulder, chamfer and vertical preparation designs. B, Surface under the cumulative ranking curves (SUCRA%) values of marginal gap values. C, The league heat diagram shows the mean difference and 95% credible interval for all possible treatment pairs.

**Internal gap evaluation using the micro-CT technique:**

The network (Supplementary material 6. Figure 19/A) included 2 in vitro studies, 1 two-arm studies and 1 multi-arm studies. The total number of examined ceramic restorations in the network was 70. SUCRA values (Supplementary material 6. Figure 19/B) indicated that vertical preparations are likely to have the smallest internal gap values (SUCRA: 91.27 %), followed by chamfer (SUCRA: 44.75 %), and rounded shoulder (SUCRA: 13.99 %). League heat plot for the internal gap (Supplementary material 6. Figure 19/C) represents the pairwise comparisons of different preparation techniques. If we compare the vertical to the chamfer (MD: 32.42 µm CrI: -32.59, 100.51), the rounded shoulder (MD: 49.19 µm CrI: -19.07, 115.15, the vertical was favored. The consistency analysis (Supplementary material 6 Figure 23.) showed that the comparisons in the network are consistent.

**
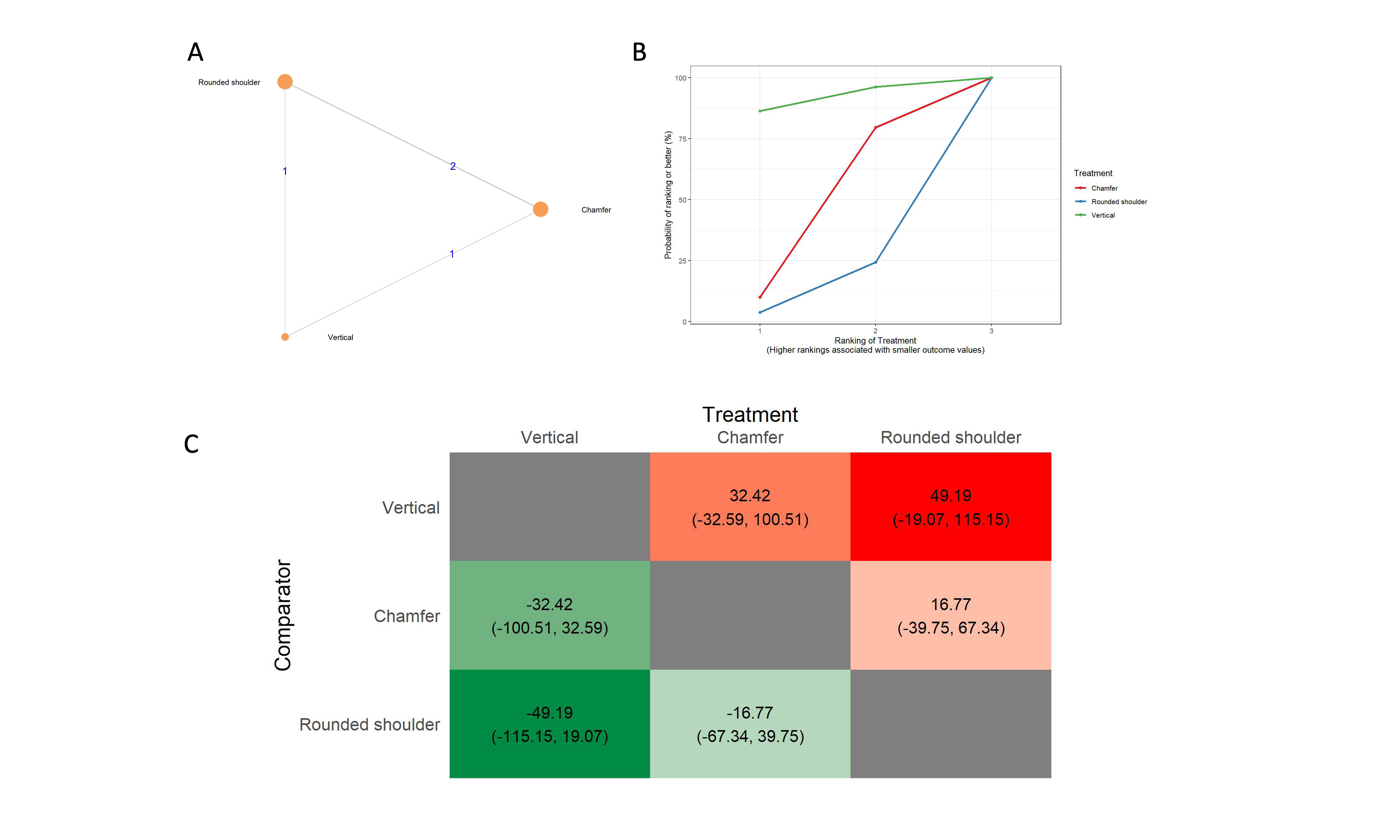
**

**Supplementary material 7. Figure 19. Internal gap evaluation using the micro-CT technique:** A, Network geometry of the eligible comparisons of internal gap evaluation using the micro-CT technique in case of rounded shoulder, chamfer and shoulder preparation designs. B, Surface under the cumulative ranking curves (SUCRA%) values of marginal gap values. C, The league heat diagram shows the mean difference and 95% credible interval for all possible treatment pairs.

**Restoration types:**

**Marginal gap of crowns, endocrowns:**

The network (Supplementary material 6. Figure 20/A) included 17 in vitro studies, 14 two-arm studies and 3 multi-arm studies. The total number of examined ceramic crowns in the network was 675. SUCRA values (Supplementary material 6. Figure 20/B) indicated that vertical preparation designs are likely to have the smallest marginal gap values (SUCRA: 71.57%), followed by rounded shoulder (SUCRA: 59.97%), chamfer (SUCRA: 44.23 %) and shoulder preparation (SUCRA: 24.25 %). League heat plot for the marginal gap (Supplementary material 6. Figure 20/C) represents the pairwise comparisons of different preparation techniques. If we compare the vertical to rounded shoulder (MD: 4.03 µm CrI: -25.26,31.74), the chamfer (MD: 8.00 µm CrI: -21.11, 35.80), and shoulder (MD: 13.10 µm CrI: -19.46, 44.95) the vertical was favored. The consistency analysis (Supplementary material 6 Figure 24.) showed that the comparisons in the network are consistent.

**
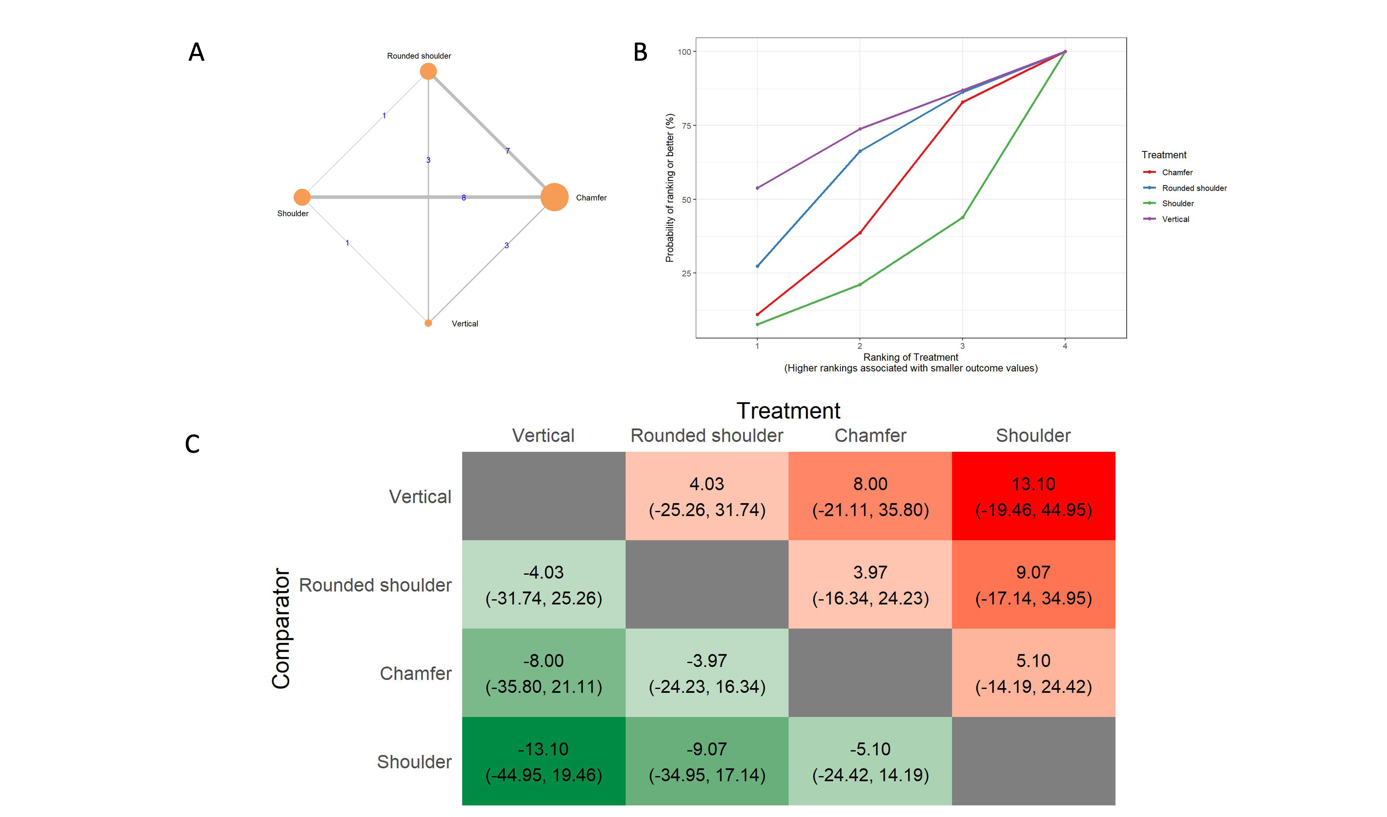
**

**Supplementary material 7. Figure 20. Marginal gap of crowns, endocrowns:** A, Network geometry of the eligible comparisons of marginal gap of crowns, endocrowns in case of rounded shoulder, shoulder and chamfer and vertical preparation designs. B, Surface under the cumulative ranking curves (SUCRA%) values of marginal gap values. C, The league heat diagram shows the mean difference and 95% credible interval for all possible treatment pairs.

**Marginal gap of copings:**

The network (Supplementary material 6. Figure 21/A) included 10 in vitro studies, 10 two-arm studies and 0 multi-arm studies. The total number of examined ceramic copings in the network was 430. SUCRA values (Supplementary material 6. Figure 21/B) indicated that shoulder preparation designs are likely to have the smallest marginal gap values (SUCRA: 72.09 %), followed by rounded shoulder (SUCRA: 51.52 %), chamfer (SUCRA: 26.39 %). League heat plot for the marginal gap (Supplementary material 6. Figure 21/C) represents the pairwise comparisons of different preparation techniques. If we compare the shoulder to rounded shoulder (MD: 3.70 µm CrI: -14.57,25.24), the chamfer (MD: 5.91 µm CrI: -8.52, 21.75), the shoulder was favored. The consistency analysis (Supplementary material 6 Figure 25.) showed that the comparisons in the network are consistent.

**
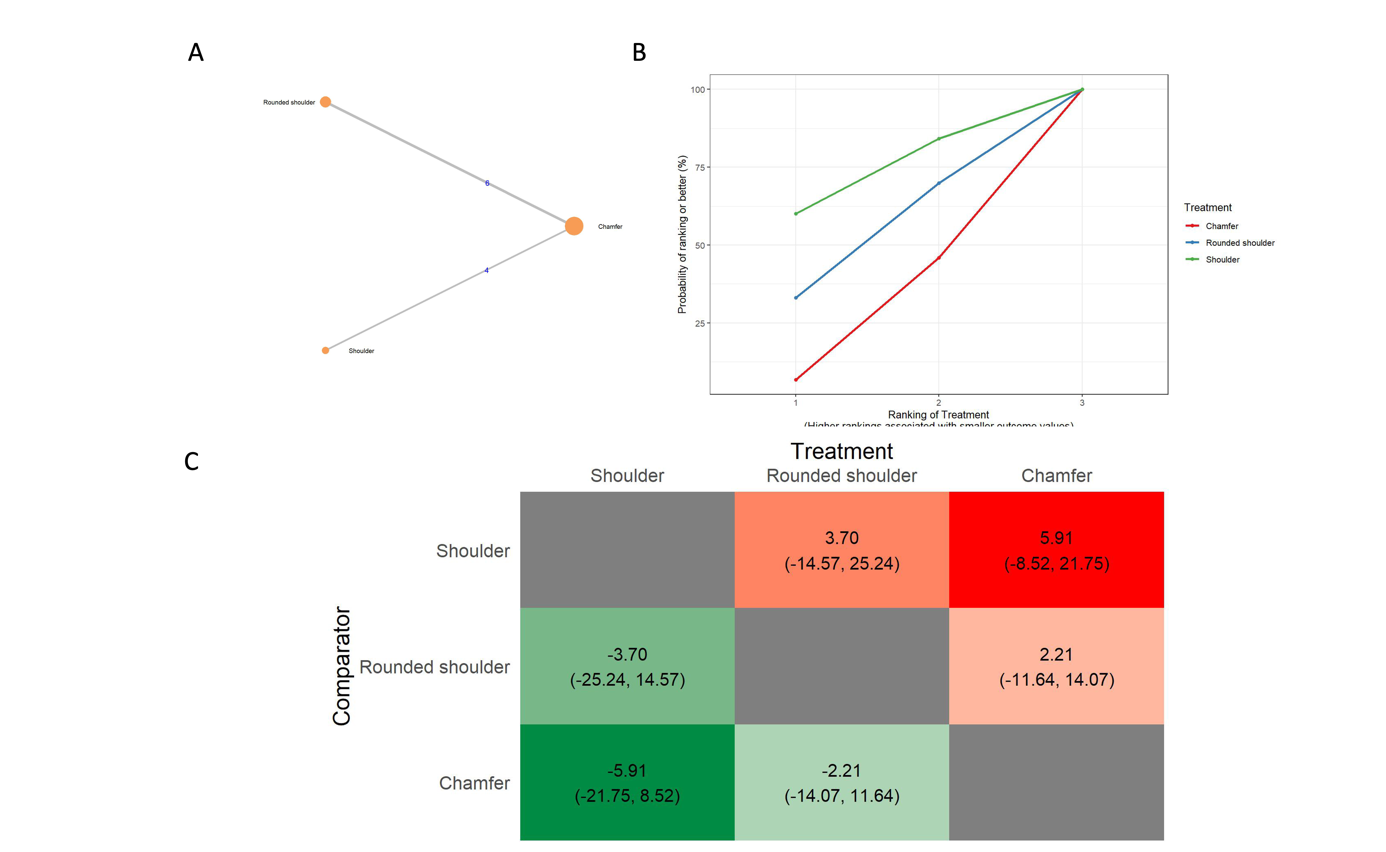
**

**Supplementary material 7. Figure 21. Marginal gap of copings:** A, Network geometry of the eligible comparisons of marginal gap of copings in case of rounded shoulder, shoulder and chamfer preparation designs. B, Surface under the cumulative ranking curves (SUCRA%) values of marginal gap values. C, The league heat diagram shows the mean difference and 95% credible interval for all possible treatment pairs.

**Marginal gap of veneers:**

The network (Supplementary material 6. Figure 22/A) included 2 in vitro studies, 1 two-arm studies and 1 multi-arm studies. The total number of examined ceramic veneers in the network was 73. SUCRA values (Supplementary material 6. Figure 22/B) indicated that vertical preparation designs are likely to have the smallest marginal gap values (SUCRA: 90.17 %), followed by rounded shoulder (SUCRA: 56.27 %), and chamfer (SUCRA: 3.56 %). League heat plot for the marginal gap (Supplementary material 6. Figure 22/C) represents the pairwise comparisons of different preparation techniques. If we compare the vertical to rounded shoulder (MD: 4.72 µm CrI: -9.79,18.77), and the chamfer (MD: 13.85 µm CrI: -0.23,28.68), the vertical was favored. The consistency analysis (Supplementary material 6 Figure 26.) showed that the comparisons in the network are consistent.

**
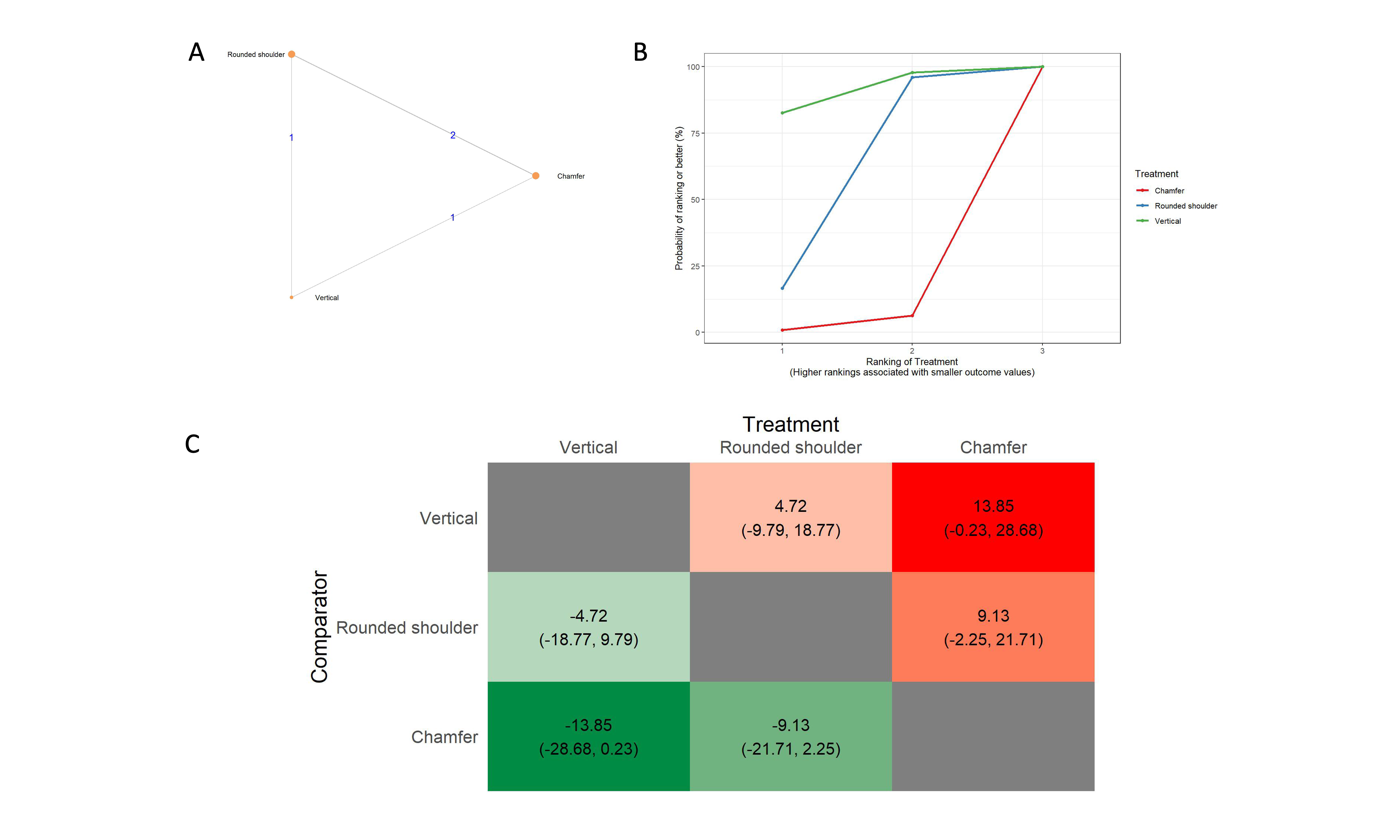
**

**Supplementary material 7. Figure 22. Marginal gap of veneers:** A, Network geometry of the eligible comparisons of marginal gap of veneers in case of rounded shoulder, vertical and chamfer preparation designs. B, Surface under the cumulative ranking curves (SUCRA%) values of marginal gap values. C, The league heat diagram shows the mean difference and 95% credible interval for all possible treatment pairs.

**Absolute marginal discrepancy of crowns, endorowns:**

The network (Supplementary material 6. Figure 23/A) included 6 in vitro studies, 4 two-arm studies and 2 multi-arm studies. The total number of examined ceramic crowns in the network was 266. SUCRA values (Supplementary material 6. Figure 23/B) indicated that rounded shoulder preparation designs are likely to have the smallest AMD values (SUCRA: 71.66%), followed by vertical (SUCRA: 50.4 %), shoulder (SUCRA: 41.17 %), and chamfer (SUCRA: 36.76 %) League heat plot for the AMD (Supplementary material 6. Figure 23/C) represents the pairwise comparisons of different preparation techniques. If we compare the rounded shoulder to vertical (MD: 9.52 µm CrI: -38.33,61.10), the shoulder (MD: 16.35 µm CrI: -67.88, 101.85), and chamfer (MD: 14.23 µm CrI: -19.46,48.34), the rounded shoulder was favored. The consistency analysis (Supplementary material 6 Figure 27.) showed that the comparisons in the network are consistent.

**
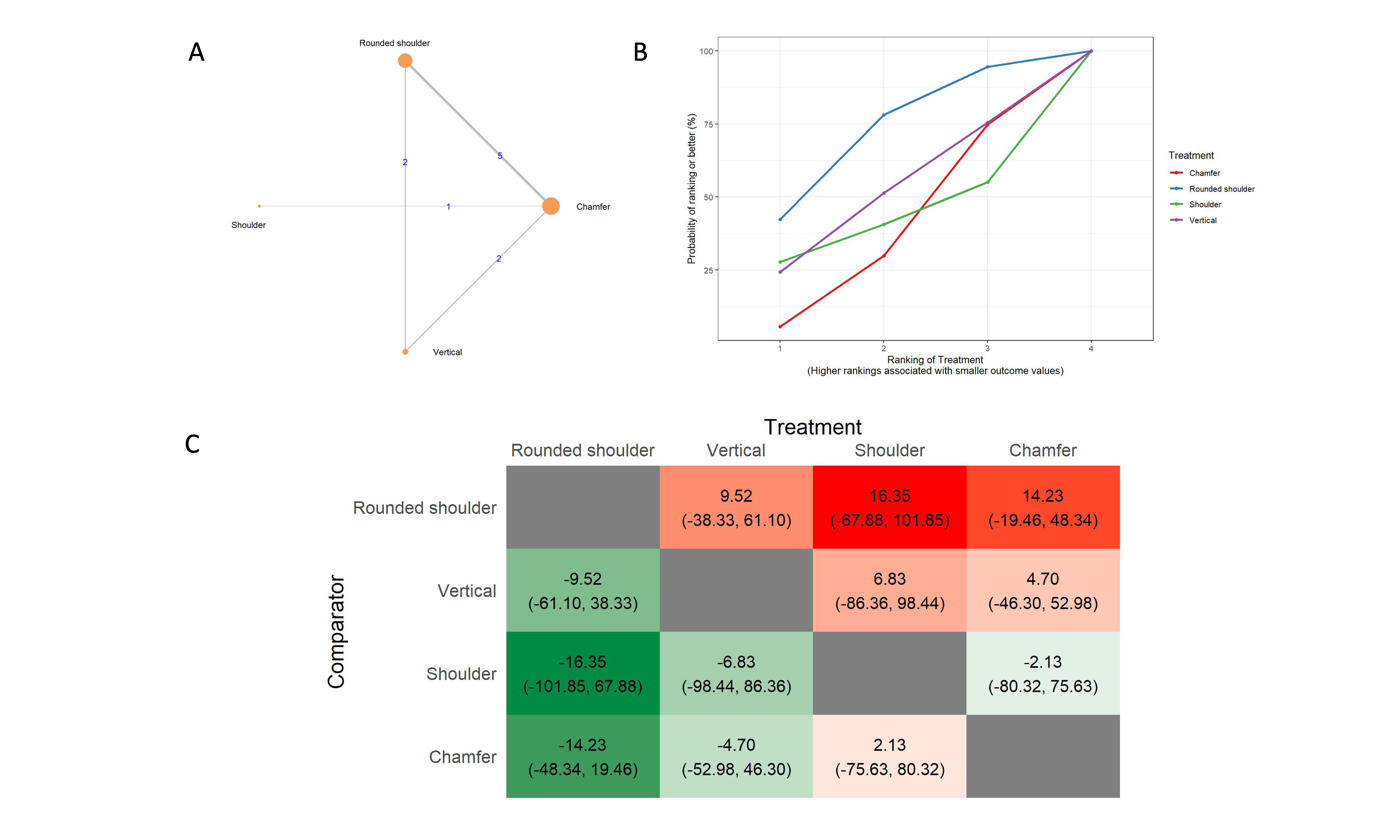
**

**Supplementary material 7. Figure 23. Absolute marginal discrepancy of crowns, endorowns:** A, Network geometry of the eligible comparisons of absolute marginal discrepancy of crowns, endorowns in case of rounded shoulder, shoulder, vertical and chamfer preparation designs. B, Surface under the cumulative ranking curves (SUCRA%) values of AMD values. C, The league heat diagram shows the mean difference and 95% credible interval for all possible treatment pairs.

**Absolute marginal discrepancy of copings:**

The network (Supplementary material 6. Figure 24/A) included 3 in vitro studies, 3 two-arm studies and 0 multi-arm studies. The total number of examined ceramic copings in the network was 100. SUCRA values (Supplementary material 6. Figure 24/B) indicated that rounded shoulder preparation designs are likely to have the smallest AMD values (SUCRA: 72.03 %), followed by shoulder (SUCRA: 59.07 %), and chamfer (SUCRA: 18.91 %). League heat plot for the AMD (Supplementary material 6. Figure 24/C) represents the pairwise comparisons of different preparation techniques. If we compare the rounded shoulder to shoulder (MD: 7.44 µm CrI: -120.54,138.70), and chamfer (MD: 38.01 µm CrI: -28.70,110.05), the rounded shoulder was favored. The consistency analysis (Supplementary material 6 Figure 28.) showed that the comparisons in the network are consistent.

**
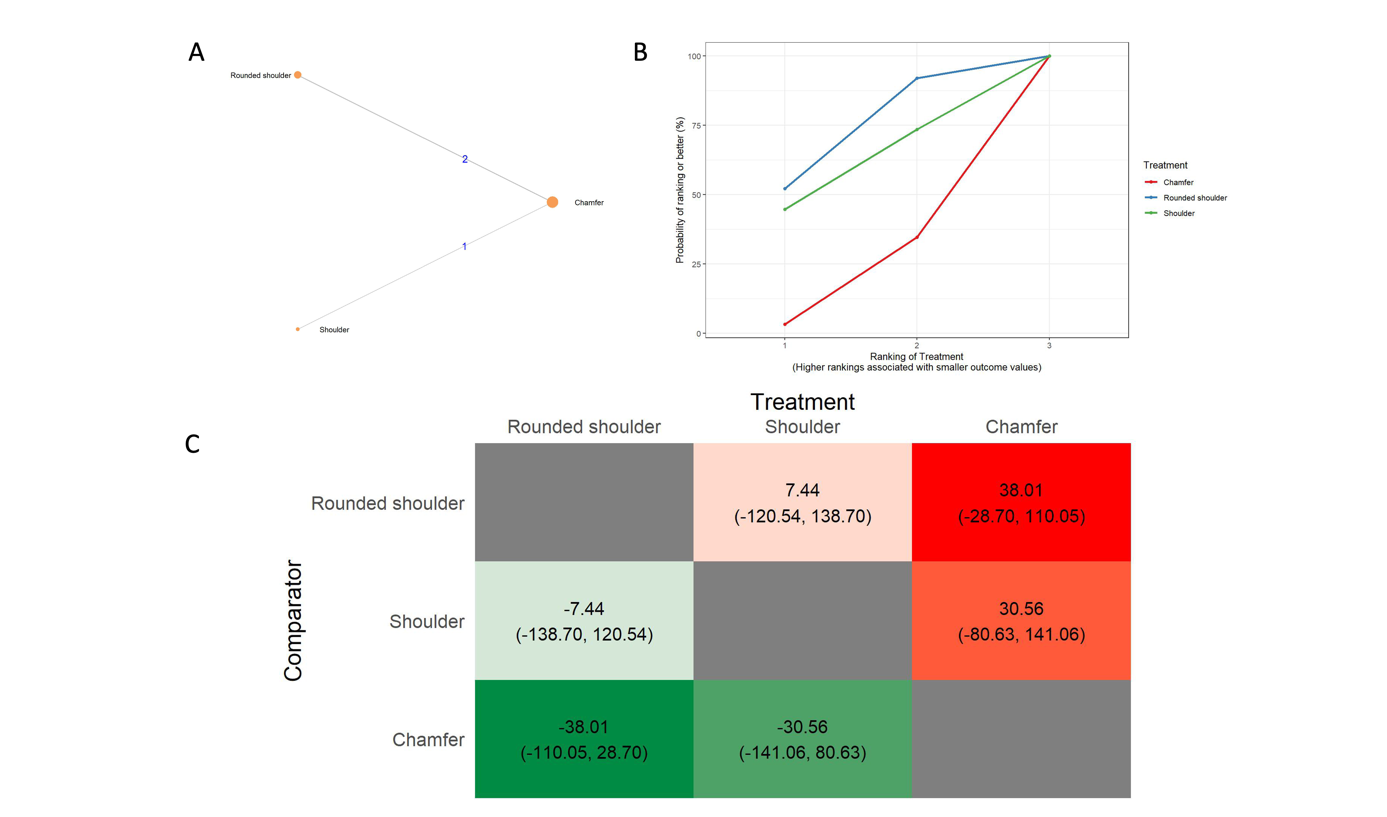
**

**Supplementary material 7. Figure 24. Absolute marginal discrepancy of copings:** A, Network geometry of the eligible comparisons of absolute marginal discrepancy of copings in case of rounded shoulder, shoulder, and chamfer preparation designs. B, Surface under the cumulative ranking curves (SUCRA%) values of AMD values. C, The league heat diagram shows the mean difference and 95% credible interval for all possible treatment pairs.

**Internal gap of crowns, endocrowns:**

The network (Supplementary material 6. Figure 25/A) included 7 in vitro studies, 5 two-arm studies and 2 multi-arm studies. The total number of examined ceramic crowns in the network was 300. SUCRA values (Supplementary material 6. Figure 25/B) indicated that chamfer preparation designs are likely to have the smallest internal gap values (SUCRA: 79.1 %), followed by vertical (SUCRA: 77.89 %), rounded shoulder (SUCRA: 24.85 %), and shoulder (SUCRA: 18.16 %). League heat plot for the internal gap (Supplementary material 6. Figure 25/C) represents the pairwise comparisons of different preparation techniques. If we compare the chamfer to the vertical (MD: -0.48 µm CrI: -36.46,34.49), rounded shoulder (MD: -26.34 µm CrI: -10.08,61.61) and shoulder (MD: 30.37 µm CrI: -7.86,68.07), the chamfer was favored. The consistency analysis (Supplementary material 6 Figure 29.) showed that the comparisons in the network are consistent.

**
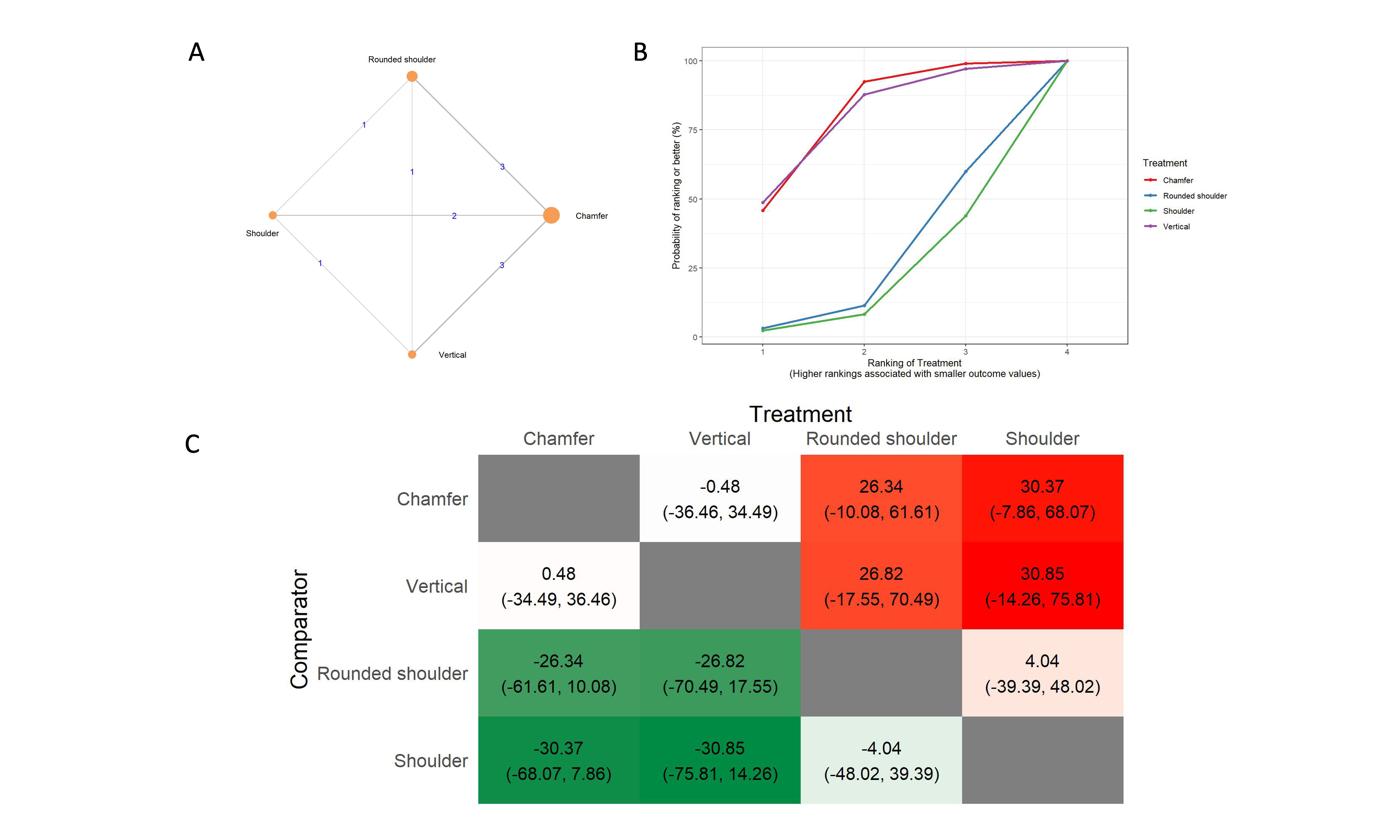
**

**Supplementary material 7. Figure 25. Internal gap of crowns, endocrowns:** A, Network geometry of the eligible comparisons of internal gap of crowns, endocrowns in case of rounded shoulder, shoulder,vertical and chamfer preparation designs. B, Surface under the cumulative ranking curves (SUCRA%) values of AMD values. C, The league heat diagram shows the mean difference and 95% credible interval for all possible treatment pairs.

**Internal gap of copings:**

The network (Supplementary material 6. Figure 26/A) included 3 in vitro studies, 3 two-arm studies and 0 multi-arm studies. The total number of examined ceramic copings in the network was 100. SUCRA values (Supplementary material 6. Figure 26/B) indicated that chamfer preparation designs are likely to have the smallest internal gap values (SUCRA: 90.66 %), followed by shoulder (SUCRA: 33.07 %), rounded shoulder (SUCRA: 26.27 %). League heat plot for the internal gap (Supplementary material 6. Figure 26/C) represents the pairwise comparisons of different preparation techniques. If we compare the chamfer to the shoulder (MD: -64.74 µm CrI: -80.20,67.07), and to rounded shoulder (MD: 67.07 µm CrI: -16.79,149.71), the chamfer was favored. The consistency analysis (Supplementary material 6 Figure 30.) showed that the comparisons in the network are consistent.

**
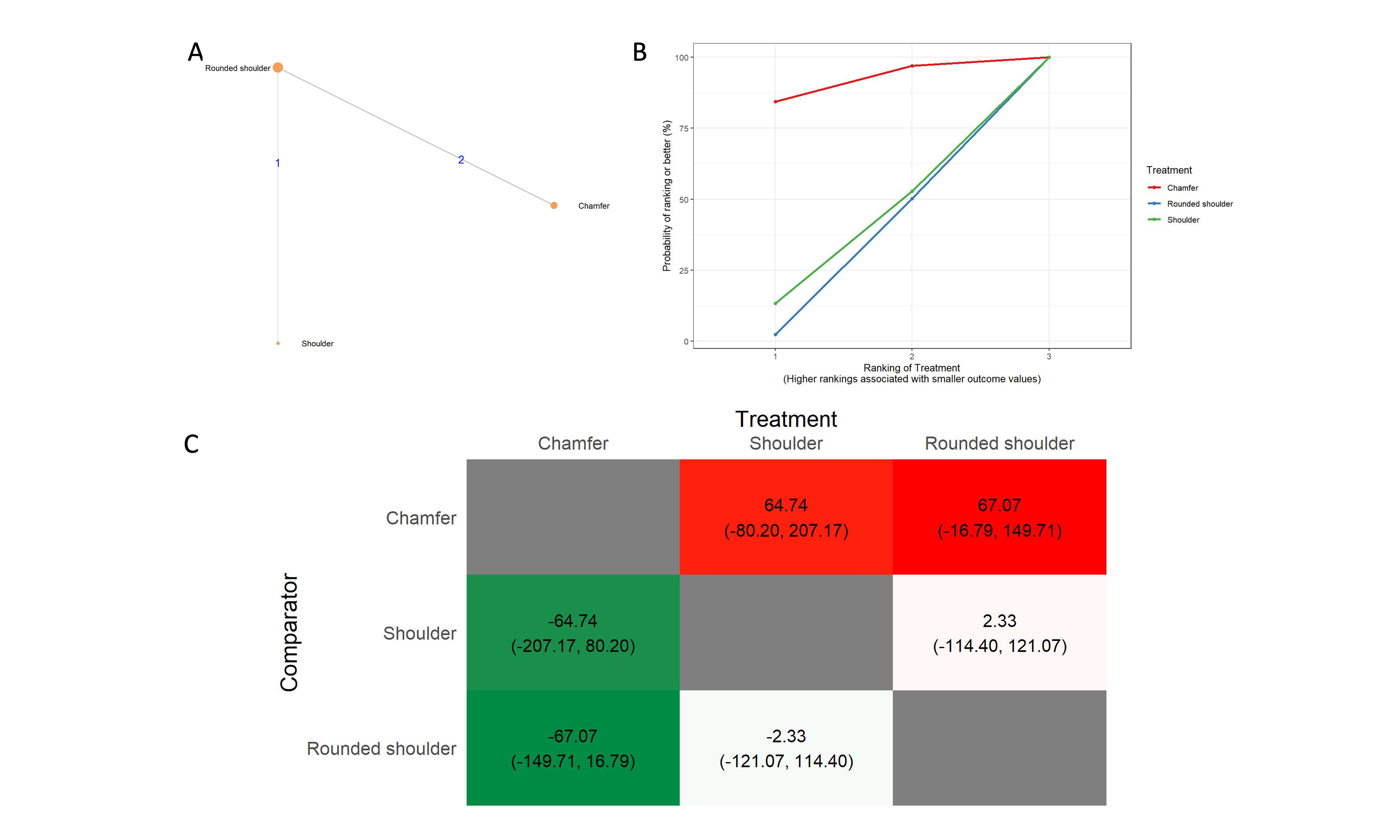
**

**Supplementary material 7. Figure 26. Internal gap of copings:** A, Network geometry of the eligible comparisons of internal gap of copings in case of rounded shoulder, shoulder and chamfer preparation designs. B, Surface under the cumulative ranking curves (SUCRA%) values of AMD values. C, The league heat diagram shows the mean difference and 95% credible interval for all possible treatment pairs.

**Internal gap of veneers:**

The comparison (Supplementary material 6. Figure 27/A) included 2 in vitro studies, 2 two-arm studies and 0 multi-arm studies. The total number of examined ceramic copings in the network was 56. SUCRA values (Supplementary material 6. Figure 27/B) indicated that rounded shoulder preparation designs are likely to have smaller internal gap values (SUCRA: 79.45 %), than chamfer (SUCRA: 20.55 %). League heat plot for the internal gap (Supplementary material 6. Figure 27/C) represents the pairwise comparisons of rounded shoulder to the chamfer (MD: 13.47 µm CrI: -19.21,46.17). The consistency analysis (Supplementary material 6 Figure 31.) showed that the comparisons in the network are consistent.


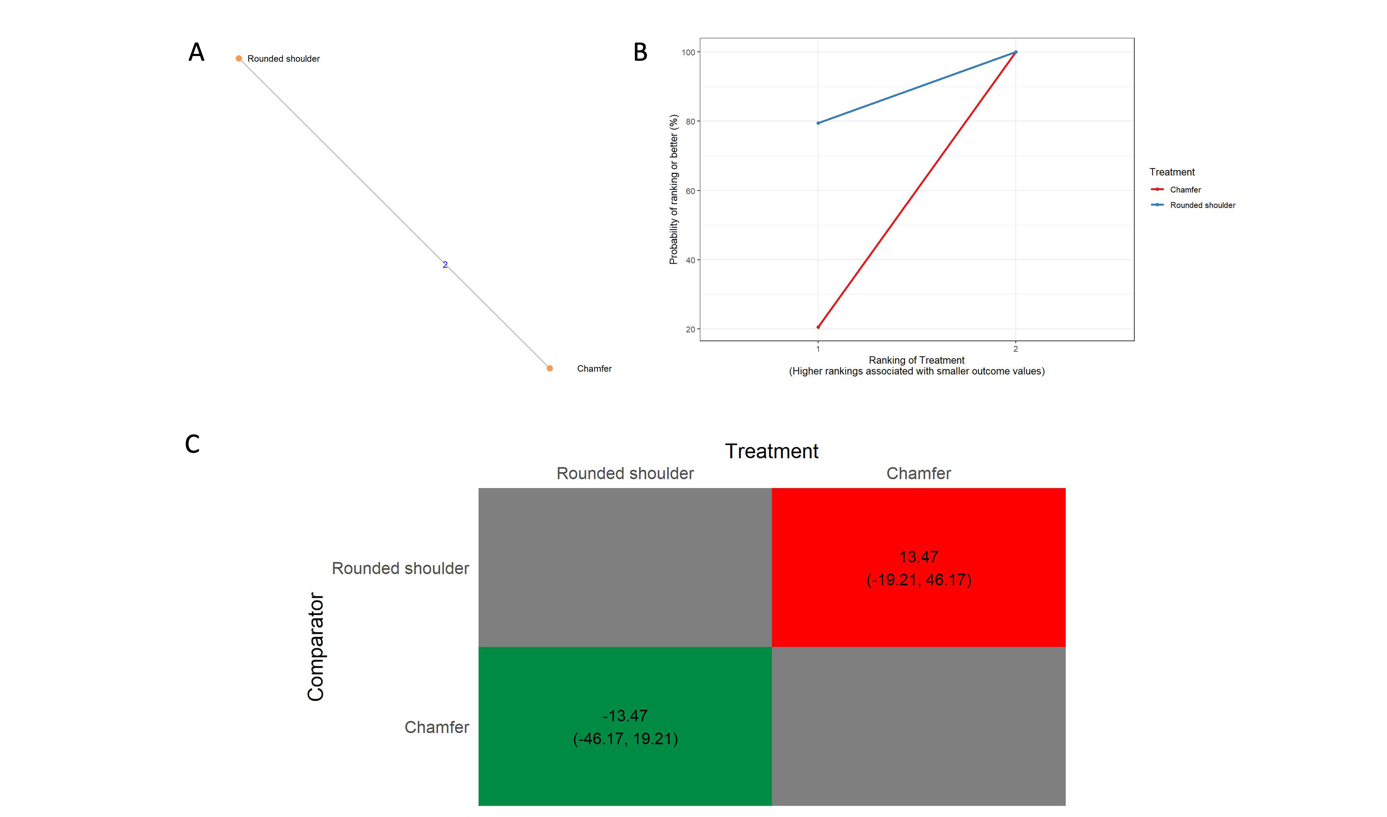


**Supplementary material 7. Figure 26. Internal gap of veneers:** A, Network geometry of the eligible comparisons of internal gap of veneers in case of rounded shoulder, and chamfer preparation designs. B, Surface under the cumulative ranking curves (SUCRA%) values of AMD values. C, The league heat diagram shows the mean difference and 95% credible interval for all possible treatment pairs.
